# The Role of Placental Hormones in Mediating Maternal Adaptations to Support Pregnancy and Lactation

**DOI:** 10.3389/fphys.2018.01091

**Published:** 2018-08-17

**Authors:** Tina Napso, Hannah E. J. Yong, Jorge Lopez-Tello, Amanda N. Sferruzzi-Perri

**Affiliations:** Department of Physiology, Development and Neuroscience, Centre for Trophoblast Research, University of Cambridge, Cambridge, United Kingdom

**Keywords:** pregnancy, placenta, hormones, maternal adaptations, metabolism, fetal growth, endocrine, cardiovascular

## Abstract

During pregnancy, the mother must adapt her body systems to support nutrient and oxygen supply for growth of the baby *in utero* and during the subsequent lactation. These include changes in the cardiovascular, pulmonary, immune and metabolic systems of the mother. Failure to appropriately adjust maternal physiology to the pregnant state may result in pregnancy complications, including gestational diabetes and abnormal birth weight, which can further lead to a range of medically significant complications for the mother and baby. The placenta, which forms the functional interface separating the maternal and fetal circulations, is important for mediating adaptations in maternal physiology. It secretes a plethora of hormones into the maternal circulation which modulate her physiology and transfers the oxygen and nutrients available to the fetus for growth. Among these placental hormones, the prolactin-growth hormone family, steroids and neuropeptides play critical roles in driving maternal physiological adaptations during pregnancy. This review examines the changes that occur in maternal physiology in response to pregnancy and the significance of placental hormone production in mediating such changes.

## Introduction

Pregnancy is a dynamic and precisely coordinated process involving systemic and local changes in the mother that support the supply of nutrients and oxygen to the baby for growth *in utero* and in the subsequent lactation. Inappropriate adaptation of maternal physiology may lead to complications of pregnancy, such as gestational diabetes, preeclampsia, fetal growth restriction, fetal overgrowth and pre-term birth; which can have immediate consequences for fetal and maternal health. Furthermore, these pregnancy complications can also lead to long-term health consequences for the mother and infant. Altered fetal growth is associated with an increased risk of the offspring developing obesity, type-2 diabetes and cardiovascular disease in adulthood (Hales and Barker, 2001; Barker, 2004; Fowden et al., 2006). Moreover, women who develop gestational diabetes or preeclampsia are more likely to develop type-2 diabetes or cardiovascular disease in later life (Kim et al., 2002; Petry et al., 2007). Maternal adaptations to pregnancy are largely mediated by the placenta; the functional interface between the mother and fetus that secretes hormones and growth factors into the mother with physiological effects. This review aims to provide an overview of the physiological changes that occur in the mother in response to pregnancy and to discuss the role of key placental hormones in mediating such adaptations. In particular, this review focuses on the importance of the prolactin-growth hormone family (e.g., prolactin, placental lactogen and growth hormone), steroids (estrogens and progesterone) and neuropeptides (serotonin, melatonin and oxytocin) in adaptations of maternal physiology during pregnancy. Where possible, this review draws upon findings in women and animal models, including rodents and sheep. However, differences exist between species in the specific hormones produced by the placenta, the access of these hormones to the maternal circulation, and the relative proportion of conceptus mass to maternal size (hence constraint on the mother to provide resources for fetal growth; Haig, 2008; Carter, 2012; Fowden and Moore, 2012). Where such differences between species exist, these have been highlighted and discussed as necessary in the relevant sections. Nevertheless, although some effects described may not be applicable to all species, the different animal models of pregnancy still provide novel insight into the fundamental mechanisms of maternal adaptation during gestation.

## Adaptations in maternal physiology during pregnancy and lactation

**Figure 1:**
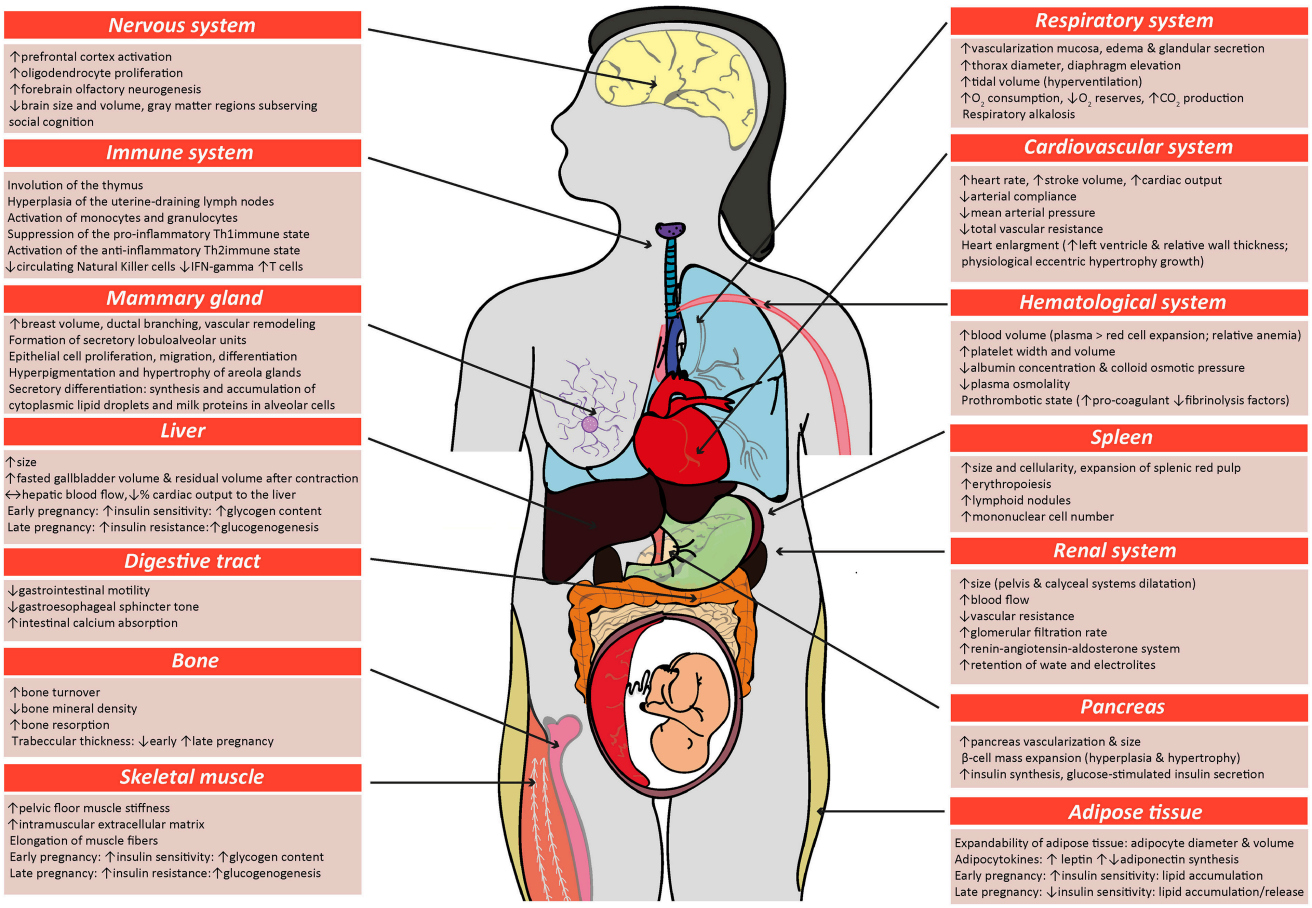
Schematic diagram highlighting the main physiological modifications in the maternal physiology in response to pregnancy. Many of the changes described in the figure for women during pregnancy also occur in other species, including mice. Respiratory system (Macrae and Palavradji, 1967; Weinberger et al., 1980; Contreras et al., 1991; Hegewald and Crapo, 2011; Frise et al., 2013; Lomauro and Aliverti, 2015; Soma-Pillay et al., 2016); cardiovascular system (Adamova et al., 2009; Li et al., 2012; Pieper, 2015; Soma-Pillay et al., 2016); hematological system (Shakhmatova et al., 2000; Chang and Streitman, 2012; Rodger et al., 2015; Soma-Pillay et al., 2016); spleen (Maroni and De Sousa, 1973; Sasaki et al., 1981; Norton et al., 2009); renal system (Davison and Dunlop, 1980; Atherton et al., 1982; Krutzén et al., 1992; Elsheikh et al., 2001; Cheung and Lafayette, 2013; Lumbers and Pringle, 2014; Pieper, 2015; Soma-Pillay et al., 2016); pancreas (Ziegler et al., 1985; Ernst et al., 2011; Ohara-Imaizumi et al., 2013; Baeyens et al., 2016); adipose tissue (Catalano et al., 2006; Hauguel-De Mouzon et al., 2006; Lain and Catalano, 2007; Nien et al., 2007; Hadden and Mclaughlin, 2009; Valsamakis et al., 2010; Musial et al., 2016); skeletal muscle (Alperin et al., 2015, 2016; Musial et al., 2016); bone (Shahtaheri et al., 1999; Ulrich et al., 2003; Hellmeyer et al., 2006; Salles, 2016); digestive tract (Everson, 1992; Fudge and Kovacs, 2010; Pieper, 2015); liver (Munnell and Taylor, 1947; Van Bodegraven et al., 1998; Lain and Catalano, 2007; Bacq, 2013); mammary tissue (Elling and Powell, 1997; Neville et al., 2002; Sternlicht, 2006; Pang and Hartmann, 2007); immune system (Clarke and Kendall, 1994; Kendall and Clarke, 2000; Veenstra Van Nieuwenhoven et al., 2002; Norton et al., 2009; Mor and Cardenas, 2010; Saito et al., 2010; Racicot et al., 2014; Groen et al., 2015; Zöllner et al., 2017; Edey et al., 2018); nervous system (Shingo et al., 2003; Gregg, 2009; Roos et al., 2011; Hoekzema et al., 2017).

Most tissues and organs in the mother respond to the pregnant state. Changes include alterations in size, morphology, function and responsiveness of tissues and organs to hormonal and metabolic cues. These changes arise in the cardiovascular, pulmonary, immune, and metabolic systems of the mother (Figure 1). Some of these changes are seen from very early in pregnancy, prior to the establishment of a fully functional placenta, highlighting that non-placental factors may also be important (Paller et al., 1989; Drynda et al., 2015). The specific nature of changes in maternal physiology depends on the stage of the pregnancy and appears to track with alterations in the metabolic requirements of the mother versus the developing fetus.

Alterations in the maternal cardiovascular system begin very early in gestation (Chapman et al., 1998) and ultimately lead to systemic vasodilation and increased blood perfusion of maternal organs, including the gravid uterus. Systemic vascular resistance is reduced by 25–30% and accompanied by a 40% increase in cardiac output during human pregnancy; while in mice, blood pressure decreases by 15% and cardiac output is increased by 48% (Bader et al., 1955; Kulandavelu et al., 2006; Soma-Pillay et al., 2016). Renal blood flow and glomerular filtration rates are also increased (Davison and Dunlop, 1980; Soma-Pillay et al., 2016). The renin-angiotensin-aldosterone system (RAAS) which is a major determinant for sodium balance during gestation, is progressively upregulated toward term with associated plasma volume expansion (Elsheikh et al., 2001; Tkachenko et al., 2014). This rise in blood volume, which is required to cope with the oxygen requirements of the maternal organs and the conceptus growth, plateaus by the late gestation, resulting in an increase in total blood volume by approximately 30% at the end of pregnancy (Chang and Streitman, 2012). There is also an increase in the numbers of red blood cells in the mother during pregnancy, due to proliferation of erythroid progenitors in the spleen (Bustamante et al., 2008). Pulmonary function is also altered and encompasses changes in ventilation rates and blood gases. For instance, lung tidal volume and minute ventilation increases by 30–50% (Hegewald and Crapo, 2011). As a result of increased oxygen consumption during hyperventilation, there is greater carbon dioxide production, which leads to chronic respiratory alkalosis that is compensated by an increased renal excretion of bicarbonate (Weinberger et al., 1980). Overall, these adaptations ensure the well-being of the mother, while also providing an adequate blood flow to the placenta for fetal nutrition, oxygenation and maturation.

There are also alterations in maternal metabolic and endocrine state during gestation. In early pregnancy, the maternal pancreatic β-cell mass expands due to both hyperplasia and hypertrophy of islets, which for example in rats, results in a >50% increase (Ackermann and Gannon, 2007; Rieck and Kaestner, 2010). The threshold for glucose-stimulated insulin production is also lowered and maternal circulating insulin concentration is greater compared to the non-pregnant state. In early pregnancy, when fetal demands are relatively low, whole body maternal insulin sensitivity is unchanged or increased and there is accumulation of energy reserves in the mother. In particular, early pregnancy is associated with adipocyte hypertrophy, increased lipogenesis and lipid storage and relates to improved insulin sensitivity of white adipose tissue in the mother (Hadden and Mclaughlin, 2009; Mcilvride et al., 2017). Interestingly, in pregnant mice, brown adipose stores of the dam also switch to a white adipose tissue-like phenotype in early gestation (Mcilvride et al., 2017). Additionally, glycogen accumulates in the liver, which also increases in size from early gestation (Bustamante et al., 2010). In contrast, late pregnancy is associated with diminished maternal tissue insulin sensitivity and a concomitant increase in lipolysis and hepatic gluconeogenesis (Freemark et al., 2002; Lain and Catalano, 2007; Musial et al., 2016). Despite the pregnancy-related rise in leptin and insulin concentrations, maternal appetite increases in pregnancy (Villar et al., 1992; Douglas et al., 2007; Hadden and Mclaughlin, 2009; Díaz et al., 2014). Together, these metabolic and endocrine alterations increase lipid and glucose availability for the rapidly growing fetus in late gestation. Intriguingly in rodents, whole body responsiveness to insulin starts to improve near term, which may be important for conserving nutrients for maternal use, as parturition and lactation approach (Musial et al., 2016). There are also notable changes in maternal bone metabolism during pregnancy. In particular, intestinal calcium absorption is enhanced in the mother during pregnancy via upregulation of 1,25-dihydroxyvitamin D levels, improved renal conservation and increased calcium mobilization from the maternal skeleton (Hellmeyer et al., 2006). These processes support the supply of calcium for the formation, growth and mineralization of the fetal skeleton (King, 2000; Kalkwarf and Specker, 2002).

The immune system of the mother during pregnancy is tightly regulated to prevent an unwanted immune response against the paternal antigens present in the developing conceptus (Racicot et al., 2014; Groen et al., 2015; Zöllner et al., 2017). As gestation progresses, there is suppression of the pro-inflammatory Th1 type of immunity and a shift toward a more anti-inflammatory, Th2 immune state in the mother (Saito et al., 2010), which supports fetal growth and maternal well-being (Mor and Cardenas, 2010). In particular, the total abundance of circulating leukocytes, monocytes, granulocytes and T lymphocytes increase in the mother in response to pregnancy (Groen et al., 2015). However, expression of major histocompatibility complex class II by circulating monocytes is reduced in the mother, which would decrease antigen presentation and stimulation of T cells during pregnancy and prevent the maternal immune system from mounting an unwanted response against fetal antigens (Groen et al., 2015). The total number of circulating natural killer cells and secretion of pro-inflammatory cytokines (IFN-gamma) is also reduced in the pregnant state (Veenstra Van Nieuwenhoven et al., 2002). However, close to parturition, the maternal immune system shifts to a pro-inflammatory state, particularly locally within the uterus, to promote labor (Mor and Cardenas, 2010; Edey et al., 2018). There are also specific changes in the numbers of different leukocyte populations in the maternal thymus and spleen during pregnancy (Clarke and Kendall, 1994; Kendall and Clarke, 2000; Norton et al., 2009). The spleen, which also has functions in hematopoiesis, enlarges due to an expansion of the splenic red pulp during pregnancy (Maroni and De Sousa, 1973; Norton et al., 2009). Neurological changes must also occur during pregnancy to increase maternal nursing behavior and enable the mother to properly care for her newborn infant (Bridges et al., 1997; Bridges, 2015; Kim, 2016; Kim et al., 2016). For instance, there is increased activation of the prefrontal cortex and neurogenesis of the forebrain olfactory bulb (Shingo et al., 2003), which are important in regulating behavior. In addition, formation of lobulo-alveolar units in the mammary gland commences during pregnancy, in preparation for lactational support of the neonate.

## Placental hormones that mediate maternal adaptations to pregnancy, parturition and lactation

The placenta is a highly active endocrine organ during gestation; secreting a variety of hormones with physiological effects in the mother. Placental hormones include members of the prolactin and growth hormone family, steroid hormones and neuroactive hormones. The function of these hormones in driving physiological changes during pregnancy has been assessed in two main ways. First, the expression and activity of the hormones have been manipulated *in vivo* by either exogenously administering or genetically manipulating the expression of hormones and hormone receptors to study the physiological consequences for the animal. Secondly, hormones have been manipulated similarly in cultured cells and tissue explants to inform on the cellular and molecular mechanisms by which they modulate function. The effects of hormones in non-pregnant animals have been included as they provide information on the baseline of physiological changes that occur in the absence of hormone expression/activity, which is especially important in the case of some placental-derived hormones, where analyses in the pregnant state have not been conducted.

### Prolactin (PRL)-growth hormone (GH) family

The PRL-GH family is one of the main families of hormones secreted by the placenta during gestation. Members of this family consist of prolactin (PRL) (Handwerger et al., 1992), placental lactogens (PLs) (Wiemers et al., 2003), PRL-like hormones (Wiemers et al., 2003), proliferins (PLF) (Lee et al., 1988), proliferin-related proteins (PRP) (Jackson et al., 1994) and growth hormone (GH). Between mammalian species, there are differences in the number and type of family members expressed by the placenta [reviewed elsewhere (Linzer and Fisher, 1999; Soares, 2004; Soares et al., 2007)]. For instance, in the mouse and rat, the placenta expresses all these members except for PRL and GH whereas the human placenta only expresses GH and PL genes. In mice and rats, expression of the individual PRL-GH family members vary spatially and temporally in the placenta (Dai et al., 2002; Simmons et al., 2008; Urbanek et al., 2015). The anterior pituitary also produces PRL and GH; however this is diminished by mid-pregnancy, when placental hormone production predominates (Bridges, 2015). In several species including rodents and humans, PRL is additionally produced by the decidua during pregnancy. The family members share structural similarity to one another and may bind, with varying affinity to PRL and GH receptors (PRLR and GHR, respectively), which are widely expressed by tissues in the body (Haig, 2008; Trott et al., 2008; Ben-Jonathan and Hugo, 2015). As the PRL-GH members also exert similar functions, these have been presented in a grouped fashion in the text and tables (Tables 1, 2). However, where possible, the roles of individual family members of the PRL-GH in physiological changes have been described.

**Table 1:** Effects of the prolactin-growth hormone family *in vivo*.

**Hormone**	**Expression level**		***In vivo* effects**	**References**
Prolactin, Placental lactogen, Prl-like hormones, Growth hormone	Low	Non-pregnant	**Prl knockout Prl**^−/−^**(mouse):** ↓ fertility; blood prolactin; mammary gland development (ductal branching, alveolar budding); oocyte maturation ↔ weight; body composition; blood lipids, adiponectin, leptin, glucose tolerance	Horseman et al., 1997; Gallego et al., 2001; Lapensee et al., 2006
			**Prl receptor knockout Prlr**^−/−^**(mouse):** ↓ fertility; weight; abdominal fat content; glucose tolerance; pancreatic β cell mass; GSIS; blood leptin, progesterone ↑ blood glucose and prolactin	Freemark et al., 2001, 2002; Rawn et al., 2015
			**Heterozygous Prl receptor knockout Prlr**^+/−^**(mouse):** ↔ body weight; glucose and insulin tolerance; GSIS; blood insulin and glucose	Binart et al., 2000
			**GH receptor knockout GHR/BP**^−/−^**(human/mouse):** ↓ body size (weight and height); postnatal growth rate; blood glucose and IGFI; sexual maturation ↑ proportional dwarfism (human), abdominal adiposity; blood GH	Zhou et al., 1997
			**Injection with GHRH antisera (rat):** ↓ growth rate; blood growth hormone	Vaccarello et al., 1993
			**GHRH knockout GHRH** ^−/−^**(mouse):** ↓weight, blood and liver IGF1, pituitary growth hormone; pituitary size; adipose tissue expression of adiponectin and visfatin; hypothalamic expression of CRH, norepinephrine; anxiety and depression related behavior ↑adiposity; food intake, blood adiponectin, ghrelin, hypothalamic expression of AgRP, NPY; exploratory activity ↔ blood leptin	Farmer et al., 1991, 1992
		Pregnancy and lactation	**Heterozygous PRL receptor knockout Prlr**^+/−^**(mouse):** ↓ pup-induced maternal behavior; post-partum nurturing behavior (pup retrieval); glucose tolerance; blood insulin; GSIS, pancreatic β cell proliferation and mass; olfactory bulb interneuron proliferation; mammary gland differentiation; milk protein expression (β-casein, whey acidic protein) ↑ blood glucose, serum metabolites ↔ body weight; insulin tolerance; blood pressure; fertility; pup weight	Horseman et al., 1997; Lucas et al., 1998; Huang et al., 2009; Rawn et al., 2015
			**Bromocriptine inhibition of Prl secretion (mouse):** ↓ milk production (↓pSTAT5) ↑ Cldn3 and Cldn4	Weinhaus et al., 1996
			**GH knockout GHR/BP**^−/−^**(mouse):** ↓ lactation (mouse)	Zhou et al., 1997
	High	Non-pregnant	**PRL overexpression (mouse):** ↑ IGF1	Wennbo et al., 1997
			**Exogenous PRL (rats):** ↓ GSIS ↑ food intake (↓ability of leptin to suppress food intake), fat deposition, blood insulin, β cell coupling	Sorenson et al., 1987; Ladyman et al., 2010
			**Exogenous GH (human):** ↓ insulin sensitivity ↑ protein synthesis; lipolytic effect of catecholamines ↔ proteolysis	Horber and Haymond, 1990
			**Exogenous PRL (mouse):** ↑ mammary gland lymphocytes	Dill and Walker, 2017
			**PLP-E overexpression (mouse):** ↑ thrombocytopenia recovery; neutropenia recovery ↔ platelet, erythrocyte, total white blood cell levels	Zhou et al., 2005
			**Pancreatic islet-specific PL-I overexpression (mouse):** ↓ blood glucose ↑ pancreatic β cell mass (↑islet proliferation and size) and insulin content; blood insulin ↔ GSIS	Vasavada et al., 2000
			**Exogenous PRL (ovariectomized rat):** ↑ induction of maternal behavior (nurturing, retrieval, nursing and crouching)	Sairenji et al., 2017
			**Transgenic human GH expression (mouse):** ↓ adiposity; insulin sensitivity; blood FFA ↑ body weight; bone density; GSIS; blood insulin and IGF1 ↔ blood glucose (fasting and glucose post challenge)	Boparai et al., 2010
			**Exogenous GH pulsatile variant (mouse):** ↓ insulin sensitivity; blood IGF1 ↑ body weight (liver, kidney, spleen); blood insulin; hepatic Ghr/Ghbp ↔ food intake, adiposity, heart weight, blood glucose	Liao et al., 2016a
			**Exogenous GH (ovariectomized rat):** ↑ induction of maternal behavior (pup retrieval)	Bridges and Millard, 1988
		Pregnancy and lactation	**Prl overexpression (mouse):** ↓ development of corpus luteum ↑ B and T cells, neutrophils and macrophage recruitment to the mammary gland (↑ chemokines; CCL2 and CXCL1); leakiness of mammary epithelial tight junctions	Galosy and Talamantes, 1995
			**Exogenous PL (hysterectomized mouse):** ↑ progesterone	Galosy and Talamantes, 1995
			**Exogenous GH (mouse):** ↓ insulin sensitivity, hepatic Glut4 ↑ blood insulin and adiposity (perirenal and gonadal), hepatic Ghr/Ghbp ↔ body weight, food intake, blood IGF1	Liao et al., 2016b
			**Exogenous GHRH (cow):** ↑ blood IGF1, somatomedin C, glucose, tri-iodothyronine (T3), insulin, NEFA; mean and pulsatile release of growth hormone; milk production; milk fat, protein, lactose yields; feed efficiency (milk production relative to food intake)	Hart et al., 1985; Enright et al., 1986, 1989; Pelletier et al., 1987; Lapierre et al., 1988; Abribat et al., 1990; Blanchard et al., 1991
			**Exogenous GHRH via injected plasmid (cow):** ↓ maternal mortality; hoof pathology ↑ number of T lymphocytes; body weight; health status (body condition scores) ↔ blood glucose or insulin	Brown P. A. et al., 2004
			**Exogenous GHRH (sheep):** ↓ blood urea ↑ blood GH, IGF1, insulin, glucose, FFA; milk yield and fat concentration	Hart et al., 1985
			**Exogenous GHRH or analog (pig):** ↑ gestation length; pregnancy weight gain; blood GH; blood and milk IGF1, offspring livebirths, weight and survival	Farmer et al., 1991, 1992, 1996; Etienne et al., 1992; Brown et al., 2012
Insulin-like growth factor 2- (Igf2)	Low	Non-pregnant	**Pancreatic** β**-cell specific Igf2 inactivation (mouse):** ↓ GSIS (aged female) ↑ insulin sensitivity ↔ glucose tolerance	Modi et al., 2015
			**Pancreatic** β**-cell specific Igf2 knockout with high-fat diet (mouse):** ↓ GSIS; pancreatic β-cell mass (only in females)	Modi et al., 2015
		Pregnancy and lactation	**Placental-specific Igf2 knockout Igf2P0 (mouse):** ↓ blood alpha-amino nitrogen; fetal and placental weight ↑ body weight; blood insulin, cortisol, leptin ↔ blood glucose	Mikaelsson et al., 2013
	High	Non-pregnant	**No known physiological changes**	Sferruzzi-Perri et al., 2007
		Pregnancy and lactation	**Exogenous (guinea pig):** ↑ visceral tissue amino acid uptake, fetal weight; placental structural and functional capacity ↔ lean mass; adiposity; blood glucose, alpha-amino nitrogen, FFA, triglycerides and cholesterol	

*AgRP, Agouti-related peptide; CRH, Corticotropin-releasing hormone; FFA, Free fatty acids; GSIS, Glucose-stimulated insulin secretion; NEFA, Non-esterified fatty acids; NPY, Neuropeptide Y*.

**Table 2:** Effects of the prolactin-growth hormone family *in vitro*.

**Hormones**	**Expression level**	***In vitro* effects**	**References**
Prolactin, Placental lactogen, Prl-like hormones, Growth hormone	Low	**siRNA knockdown of PRL receptor (rat pancreatic** β**-cells):** ↓ DNA synthesis (↓ cyclin B2 and D2, IRS-2, Tph1) ↑ apoptosis (↓anti-apoptotic proteins PTTG1, p21 and BCL6) ↔β cell replication or survival related genes (p18, p19, Cyclin D3, CDK2, CDK4, CDK6, IGF2, BAX, or TLR4)	Arumugam et al., 2014
		**siRNA knockdown of GH (hen granulosa cells primary culture):** ↓ proliferation	Ahumada-Solórzano et al., 2016
	High	**Exogenous PRL, PLP, GH (human, rat and mouse islets):** ↓ apoptosis (↑anti- apoptotic proteins; p21 and BCL6) ↑β-cell mass; GSIS; DNA synthesis, β-cell replication or survival related genes (cyclins A2, B1, B2 and D2, IRS-2, Tph1, FoxM1, BCLxL and PTTG1); insulin secretion and glucose oxidation (only PRL;↑glucokinase, hexokinase and GLUT2 expression); serotonin biosynthesis (Tph1, Tph2, Jak2, STAT5) ↔β cell replication or survival related genes (p18, p19, Cyclin D3, CDK2, CDK4, CDK6, IGF2, BAX, or TLR4).	Nielsen, 1982; Brelje et al., 1989, 1993; Weinhaus et al., 1996; Sorenson and Brelje, 1997; Arumugam et al., 2014
		**Exogenous PRL (mouse alveolar mammary epithelial cells):** ↓ milk protein expression (β-casein) ↑ leaky tight junctions (↓Tj transmembrane proteins: Cldn3, Cldn4)	Kobayashi et al., 2016
		**Exogenous PRL (human fetal membranes**+**LPS):** ↓ TNF-α, IL-1β ↔ IL-6, IL-10	Flores-Espinosa et al., 2017
		**Exogenous PRL (rat uterine stromal cells):** ↓ decidualization (PGE2, PGF2α); cytolytic activity ↔ cell viability; proliferation	Prigent-Tessier et al., 1996
		**Exogenous PL (mouse ovarian cells):** ↑ progesterone secretion	Galosy and Talamantes, 1995
		**Exogenous PRL (rat uterine NK cells):** ↓ cytolytic activity ↔ cell viability; proliferation	Müller et al., 1999
		**PLPE transfection (human and murine erythroid cells):** ↑ proliferation; differentiation (hemoglobin production)	Bittorf et al., 2000
		**Exogenous PLF (bovine capillary endothelial cells):** ↑ angiogenesis- endothelial cell migration (through MAPK activation and IGF-II/mannose 6-phosphate receptor interaction)	Groskopf et al., 1997
		**PLP-E/F exogenous (mouse bone marrow):** ↑ megakaryocyte differentiation, progenitor growth (colony formation)	Zhou et al., 2002
		**Exogenous PLF (mouse neuroblastoma cells):** ↑ microvilli formation; proliferation	Wang et al., 2006
		**Exogenous GH (hen granulosa cells primary culture):** ↑ proliferation; IGF1 secretion	Ahumada-Solórzano et al., 2016
		**Exogenous GHRH (sheep and rat pituitary cells):** ↑ GH secretion; IGF1 secretion	Blanchard et al., 1991
		**Exogenous GH (rat ovarian granulosa cells):** ↓ LH-stimulated progesterone production ↑ progesterone production; cAMP accumulation	Apa et al., 1995
Insulin-like growth factor 2 (Igf2)	Low	**Treatment with Igf2 mutant** + **prolactin (bovine capillary endothelial cells):** ↓ motility; MAPK activity	Groskopf et al., 1997
		**IGF2R siRNA knockdown (BeWo and human placental villous explants):** ↓ apoptosis ↑ IGF2-stimulated mitosis	Harris et al., 2011
		**IGF2 knockdown (human hemangioma stem cells):** ↓ cell differentiation; leptin induction ↔ proliferation	Kleiman et al., 2013
	High	**Exogenous (human endothelial cells):** ↑ migration; angiogenesis	Lee et al., 2000
		**Exogenous (chick chorioallantoic membrane):** ↑ angiogenic activity; migration	Bae et al., 1998
		**Exogenous (human keratinocyte cell line, human liver carcinoma cell line):** ↑ VEGF	Bae et al., 1998
		**Adenoviral-mediated overexpression (mouse pancreatic** β **cells):** ↓β cell differentiation; islet function (↑ glucose intolerance and↓ insulin release)	Casellas et al., 2015
		**Exogenous (bovine granulosa cells):** ↑ proliferation; estradiol and progesterone production; aromatase (CYP19A1) mRNA	Spicer and Aad, 2007
		**Exogenous (mouse primary hepatocytes):** ↑ proliferation	Bae et al., 1998

*BAX, BCL2 associated X; CDK, Cyclin dependent kinases; GSIS, Glucose-stimulated insulin secretion; IRS2, Insulin receptor substrate 2; LPS, Lipopolysaccharide; MAPK, Mitogen-activated protein kinase; PGE, Prostaglandin E synthase; PGF2α, Prostaglandin F2α; PTTG1, Pituitary tumor-transforming 1; siRNA, short interfering RNA; TLR4, Toll-like receptor; VEGF, Vascular endothelial growth factor*.

Studies performed both *in vivo* and *in vitro* support a role for the PRL-GH family in mediating maternal metabolic adaptations to pregnancy (Tables 1, 2). PRL, PRL-like proteins and PL, principally via the PRL receptor, induce β-cell mass expansion by both increasing β-cell proliferation and reducing apoptosis of islets *in vivo* and *in vitro* (Table 2; PRL/PL/GH; Brelje et al., 1993; Huang et al., 2009). PRL and PL also increase insulin secretion during pregnancy, particularly in response to glucose, by enhancing the expression of glucose sensors (glucokinase, hexokinase and glucose transporter-2) and activating the serotonin biosynthesis pathway in pancreatic islets (Table 2; PRL/PL/GH; Nielsen, 1982; Brelje et al., 1989, 1993; Weinhaus et al., 1996; Sorenson and Brelje, 1997; Arumugam et al., 2014). Moreover, PL protects β-cells against streptozotocin-induced cell death in mice (Fujinaka et al., 2004). GH may also be important for modulating pancreatic insulin production (Billestrup and Nielsen, 1991; Brelje et al., 1993). However, GH from the placenta appears to be primarily important in the acquisition of insulin resistance and shifting metabolic fuel use from glucose to lipid in the mother during pregnancy (Table 1; PRL/PL/GH; Horber and Haymond, 1990; Goodman et al., 1991; Galosy and Talamantes, 1995; Barbour et al., 2002; Dominici et al., 2005; Boparai et al., 2010; Liao et al., 2016b; Sairenji et al., 2017). Placental GH reduces insulin receptor expression and signaling, as well as, diminishes the abundance of the insulin-sensitive glucose-transporter, GLUT-4, in the skeletal muscle (Barbour et al., 2004; Kirwan et al., 2004). Insulin receptor abundance and signaling in the liver is also reduced in response to increased GH abundance in transgenic mice (Dominici et al., 1999). In white adipose tissue, GH also disrupts the insulin signaling pathway, and inhibits insulin action on glucose uptake and lipid accumulation (Del Rincon et al., 2007). In part, the effects of GH may be mediated through insulin-like growth factor-1 (IGF1), which is primarily secreted from the liver in response to GH and exerts lipolytic effects during pregnancy (Randle, 1998; Sferruzzi-Perri et al., 2006; Del Rincon et al., 2007). Insulin-like growth factor-2 (IGF2), which is not directly regulated by GH, but is secreted by the placenta is also important for modulating the sensitivity of β cells to glucose (Tables 1, 2; IGF2; Casellas et al., 2015; Modi et al., 2015) and maternal insulin and glucose concentrations during pregnancy (Petry et al., 2010; Sferruzzi-Perri et al., 2011). Polymorphisms/mutations in the PRL-GH family of genes and receptors have been reported in human pregnancies associated with gestational diabetes and fetal growth restriction (Rygaard et al., 1998; Le et al., 2013). Moreover, loss of PRLR signaling in β-cells causes gestational diabetes mellitus (GDM) in mice (Banerjee et al., 2016). Taken together, the production of PRL-GH family of hormones by the placenta appears to be important in regulating both insulin production and sensitivity of the mother in response to pregnancy.

The PRL-GH family is also implicated in the regulation of appetite and body weight. For instance, exogenous PRL increases food intake through inhibiting the action of leptin in non-pregnant rats (Table 1; PRL/PL/GH; Sorenson et al., 1987; Farmer et al., 1991, 1992; Ladyman et al., 2010). In contrast, GH appears to decrease food intake in rodents through reducing ghrelin production and hypothalamic expression of appetite-stimulating neuropeptides, AgRP and NPY (Table 1; PRL/PL/GH; Farmer et al., 1991, 1992). In non-pregnant animals, GH is important for controlling body weight and composition (such as adiposity; Farmer et al., 1991, 1992; Zhou et al., 1997). However, in pregnancy, exogenous GH or GH releasing hormone (GHRH) does not appear to affect maternal weight gain in mice, although increases it in pigs (Table 1; PRL/PL/GH; Brown et al., 2012). The effect of PRL on weight gain and body adiposity is even less clear; with both no effect and an increase reported for non-pregnant and pregnant rodents.

The PRL-GH family also plays an important role in lactation and maternal behavior. In mice, a deficiency in PRLR or inhibition of PRL secretion *in vivo* compromises mammary gland development, differentiation and milk production; the latter of which is associated with loss of STAT5 signaling and fewer leaky tight junctions (Table 1; PRL/PL/GH; Weinhaus et al., 1996; Zhou et al., 1997). In contrast, exogenous GHRH in sheep and cows increases mammary gland milk production (Hart et al., 1985; Enright et al., 1988). There is also evidence that PRL induces maternal behaviors, such as nurturing, nursing and pup retrieval in non-pregnant rodents (Table 1; PRL/PL/GH; Bridges and Millard, 1988). Taken together, members of the PRL-GH family appear to promote changes in maternal glucose metabolism, behavior and mammary gland function which are expected to be important for supporting the growth of offspring during pregnancy and lactation.

### Steroid hormones

The placenta is a primary source of steroid hormones during pregnancy. Placental steroid hormones include estrogens and progesterone (Costa, 2016; Edey et al., 2018). In species like rodents, the corpus luteum continues to contribute to the circulating pool of steroid hormones during pregnancy, whereas in other species such as humans and ruminants, the placenta serves as the main source (Costa, 2016). Physiological effects of progesterone are mediated predominately by nuclear receptors (PR-A, PR-B) although membrane bound-type receptors (mPR) enable non-genomic actions. Steroid hormones are implicated in pregnancy complications such as gestational diabetes and preeclampsia. High progesterone and estrogen concentrations have been reported for women with gestational diabetes (Branisteanu and Mathieu, 2003; Qi et al., 2017). Moreover, placental estrogen and progesterone levels are reduced in preeclamptic patients compared with healthy pregnant women (Açikgöz et al., 2013).

Studies performed *in vivo*, suggest placental steroid hormones may be important in driving the changes in insulin sensitivity and glucose metabolism of the mother during pregnancy (Table 3). Hyperinsulinemic-euglycemic clamp studies in women and rodents highlight a role for progesterone in reducing maternal insulin sensitivity during pregnancy. Progesterone administration decreases the ability of insulin to inhibit glucose production by the liver, and diminishes insulin-stimulated glucose uptake by skeletal muscle and to a lesser extent in the adipose tissue of non-pregnant animals (Table 3; Progesterone; Leturque et al., 1984; Ryan et al., 1985; Kim, 2009). In contrast, exogenous estrogen increases whole body insulin sensitivity in non-pregnant state (Table 3; Estrogen; Ahmed-Sorour and Bailey, 1980). Similarly, genetic deficiency of ERα or aromatase (Cyp19), which is involved in estrogen production, reduces hepatic and whole body insulin sensitivity and impairs glucose tolerance in non-pregnant mice (Takeda et al., 2003; Bryzgalova et al., 2006). Loss of the estrogen receptor or estrogen production is also associated with increased body weight, adiposity and hepatic lipogenesis (Table 3; Estrogen; Takeda et al., 2003; Bryzgalova et al., 2006). Progesterone and estrogen also exert opposite effects on food intake *in vivo* (Table 3). In particular, estrogen depresses food intake in part via induction of leptin production by adipose tissue, whereas progesterone increases food intake by enhancing NPY and reducing CART expression by the hypothalamus (Table 3; Fungfuang et al., 2013; Stelmanska and Sucajtys-Szulc, 2014). Estrogen and progesterone however seem to have similar effects on the pancreas; they both appear to induce islet hypertrophy and/or increase pancreatic insulin levels and glucose-stimulated secretion *in vivo* (Table 3; Costrini and Kalkhoff, 1971; Bailey and Ahmed-Sorour, 1980). Nevertheless, there is some evidence that progesterone may inhibit the PRL-induced proliferation and insulin secretion of β cells *in vitro* (Table 4; Progesterone; Sorenson et al., 1993). Furthermore, in rodent models of type 1 and 2 diabetes mellitus, estrogen supplementation protects pancreatic β-cells from oxidative stress, lipotoxicity and apoptosis (Table 3; Estrogen; Tiano and Mauvais-Jarvis, 2012). Therefore, both estrogen and progesterone play roles in regulating insulin and glucose homeostasis, lipid handling and appetite regulation, which may be important in promoting metabolic changes in the mother during pregnancy.

**Table 3:** *In vivo* effects of steroid hormones *in vivo*.

**Hormones**	**Expression level**	***In vivo*** **effects**		**References**
Estrogen	Low	Non-pregnant	**Estrogen receptor knockout ER**^−/−^**(ERKO, BERKO or viral-mediated ER suppression mouse):** ↓ glucose tolerance; whole body and hepatic insulin sensitivity; insulin-stimulated glucose uptake by skeletal muscle; blood adiponectin, testosterone; sexual behavior ↑ body weight; abnormalities in vascular smooth muscle cells (ion channel function); systolic and diastolic blood pressure; arterial pressure; heart failure; hepatic lipid biosynthesis; adipose tissue mass; blood glucose, insulin, leptin	Zhu et al., 2002; Bryzgalova et al., 2006; Ribas et al., 2011
			**Aromatase knockout CYP19–/– (mouse):** ↓ glucose and insulin tolerance; glucose oxidation; lean body mass ↑ body weight; adipocyte volume; blood glucose and testosterone	Yeh et al., 2002; Takeda et al., 2003
		Pregnancy and lactation	**Estrogen receptor knockout ER**^−/−^**(ERKO, BERKO or viral-mediated ER suppression mouse):** ↓ litter size; maternal nurturing behavior (time spent nursing pups) ↔maternal aggression toward a male intruder	Ribeiro et al., 2012
			**ER antagonist (guinea pig):** ↓ NOS activity in the cerebellum	Weiner et al., 1994
	High	Non-pregnant	**Exogenous estrogen in T1DM, T2DM model (mouse):** ↓ oxidative stress (β cells); apoptosis; amyloid polypeptide toxicity; lipotoxicity	Tiano and Mauvais-Jarvis, 2012
			**Exogenous (ovariectomized rat or mouse):** ↓ hepatic glucose production; blood glucose; TNF-α macrophage synthesis; gluconeogenesis; food intake (via ↑ leptin) ↑ insulin sensitivity; glycogen storage; VEGF, PlGF (angiogenesis); eNOS production; arterial vasodilatatory responses ↔ body weight	Ahmed-Sorour and Bailey, 1980, 1981; Zhang et al., 1999; Storment et al., 2000; Fungfuang et al., 2013
		Pregnancy and lactation	**Exogenous (ovariectomized mouse):** ↓ intimal cell proliferation in response to vessel injury	Zhang et al., 1999
Progesterone	Low	Non-pregnant	**Progesterone receptor knockout PR**^−/−^**(mouse):** ↓ reproductive tissue development; ovulation; mammary gland development; sexual behavior ↑ uterine mass, inflammation	Lydon et al., 1995
		Pregnancy and lactation	**Exogenous antagonist (rat):** ↓ oxytocin production; oxytocin receptor synthesis ↑ premature birth; blood estrogen; oxytocin receptor synthesis	Fang et al., 1997
			**Exogenous antagonist (mouse):** ↑ preterm parturition; myometrial monocytes near parturition (Cx-43)	Edey et al., 2018
	High	Non-pregnant	**Exogenous (mice):** ↑ mammary gland lateral branching and number of stem cells	Joshi et al., 2010
			**Exogenous (ovariectomized rat):** ↓ insulin-dependent suppression of endogenous hepatic glucose production ↑ insulin resistance in the liver, skeletal muscles and adipose tissue; eNOS expression in the abdominal aortas, food intake (via ↑NPY, ↓CART) ↔ insulin-mediated glucose uptake (peripheral tissues), body weight	Fang et al., 1997; Stelmanska and Sucajtys-Szulc, 2014
			**Exogenous (mink):** ↑ uterine glycogen catabolism, glucose release	Dean et al., 2014
		Pregnancy and lactation	**Exogenous (mouse):** ↑ myometrial monocyte numbers ↔ myometrial neutrophil numbers	Edey et al., 2018
			**Exogenous (ovariectomized mice):** ↓ intimal proliferation in response to vessel injury ↑ anti-anxiety behavior (↑ hippocampal and prefrontal cortex 3α,5α-THP)	Koonce and Frye, 2013

*eNOS, Endothelial nitric oxide synthase; PlGF, Placental growth factor; T1DM, Type 1 diabetes mellitus; T2DM, Type 2 diabetes mellitus; THP, Tetrahydroprogesterone; TNF, Tumor necrosis factor; VEGF, Vascular endothelial growth factor*.

**Table 4:** Effects of steroid hormones *in vitro*.

**Hormones**	**Expression level**	***In vitro* effects**	**References**
Estrogen	Low	**ER antagonist (human cytotrophoblast):** ↓ proliferation	Kumar et al., 2009
	High	**Exogenous (rat islets):** ↔ PRL-induced β cell proliferation or insulin secretion	Sorenson et al., 1993
		**Exogenous (human umbilical venous endothelial cells):** ↓ folic acid induced anti-angiogenic action ↑ leptin expression; differentiation	Lee et al., 2017
		**Exogenous (human, mouse, rat vascular smooth muscle cells):** ↓ proliferation (↑ cell cycle arrest at G1) ↑ eNOS activity	Haynes et al., 2000; Hisamoto et al., 2001; Takahashi et al., 2003
		**Exogenous (pregnant ewe uterine artery endothelial cells):** ↑ angiogenesis (↑cell proliferation)	Jobe et al., 2010
		**Exogenous (rat coronary arteries):** ↓ vasodilation (gender and oestrous cycle dependent) ↑ nitric oxide production (NOS)	Binko and Majewski, 1998; Shaw et al., 2001
		**Exogenous (human myometrial cells):** ↑ myometrial gap junction communication	Di et al., 2001
Progesterone	Low	**No known physiological effects**	
	High	**Exogenous (rabbit, rat coronary arteries):** ↑ coronary relaxation (calcium influx dependent)	Jiang et al., 1992
		**Exogenous (human umbilical vein endothelial cells):** ↓ LPS-induced leukocyte adhesion ↑ nitric oxide synthesis	Simoncini et al., 2004
		**Exogenous (human umbilical venous endothelial cells):** ↓ leptin-induced invasion; folic acid-induced anti-angiogenic action; LPS-induced cytokine secretion (TNF-α, IL-1β, IL-6, IL-8, MIP-1α, IL-10)	Garcia-Ruíz et al., 2015; Jo et al., 2015; Lee et al., 2017
		**Exogenous (rat islets):** ↓ PRL-induced β cell proliferation and insulin secretion	Sorenson et al., 1993
		**Exogenous (mouse mammary epithelial cell):** ↑ proliferation; DNA synthesis; lobulo-alveoli development	Plaut et al., 1999; Obr et al., 2013
		**Exogenous (human T cells):** ↓ CD4 and CD8 T cell proliferation and production of IFN-γ, TNF-α, IL-10, IL-5, IL-17, CD4 ↑ CD4 and CD8 T cell production of IL-4	Lissauer et al., 2015

CD, Cluster of differentiation; eNOS, Endothelial nitric oxide synthase; IL, Interleukin; LPS, Lipopolysaccharide; TNF, Tumor necrosis factor

Work conducted both *in vitro* and *in vivo* indicate that estrogen and progesterone may also facilitate some of the cardiovascular changes that accompany pregnancy (Tables 3, 4). Estrogen attenuates the vasoconstrictor responses of blood vessels, impairs vascular smooth muscle cell proliferation and calcium influx, and increases vasodilatory nitric oxide synthase activity *in vitro* (Table 4; Estrogen; Takahashi et al., 2003). It also increases uterine artery angiogenesis and amplifies the vasodilatory impact of vascular endothelial growth factor on isolated rat uterine vessels (Storment et al., 2000; Jobe et al., 2010). In non-pregnant mice, deficiency of the ERβ gene leads to defects in vascular smooth muscle function, hypertension and signs of heart failure (Table 4; Estrogen; Zhu et al., 2002; Fliegner et al., 2010). Conversely, estrogen supplementation appears to protect the heart and vasculature from pressure overload or vessel injury (Zhang et al., 1999; Zhu et al., 2002; Fliegner et al., 2010). Progesterone also exerts cardiovascular effects. It stimulates nitric oxide synthesis by human umbilical vein endothelial cells *in vitro* and by rat abdominal aorta and mesenteric arteries *in vivo* (Tables 3, 4; Progesterone; Chataigneau et al., 2004; Simoncini et al., 2004). It also decreases blood pressure, when infused into ovariectomised ewes and protects against vascular injury in non-pregnant mice (Pecins-Thompson and Keller-Wood, 1997; Zhang et al., 1999). In culture, progesterone induces hypertrophy and inhibits apoptosis of rodent cardiomyocytes (Morrissy et al., 2010; Chung et al., 2012). Thus, via its impacts on cardiomyocytes, progesterone may mediate the pregnancy-induced growth of the mother's heart *in vivo*. In late pregnancy, the murine heart shifts to use fatty acids, rather than glucose and lactate, as a metabolic fuel. In part, this metabolic shift is proposed to be mediated by progesterone during pregnancy, which inhibits pyruvate dehydrogenase activity in ventricular myocytes (Liu et al., 2017). Thus, placental-derived progesterone and estrogen may mediate part of the changes in the maternal cardiovascular system during pregnancy.

In many mammalian species, progesterone levels decline just before parturition and this is associated with the initiation of labor. Indeed, in rodents, inhibition of progesterone synthesis or administration of a progesterone antagonist results in premature delivery of the neonate (Table 3; Progesterone; Fang et al., 1997; Kota et al., 2013). In humans, circulating progesterone levels continue to be high until birth. Commencement of labor is therefore proposed to be related to a functional withdrawal of progesterone activity in the myometrium of women (Brown A. G. et al., 2004; Norwitz and Caughey, 2011). In experimental animals, progesterone reduces the production of prostaglandins and decreases the expression of contraction-associated genes including oxytocin and prostaglandin receptors, gap junction proteins and ion channels in the myometrium (Table 3; Progesterone; Fang et al., 1997; Soloff et al., 2011; Edey et al., 2018). Together, these progesterone-mediated actions decrease contractility of uterine smooth muscle cells and maintain uterine quiescence until term. In contrast to progesterone, estrogen levels rise prior to term and estrogen promotes the expression of contraction-associated genes and contraction of the myometrium (Table 4; Estrogen; Nathanielsz et al., 1998; Di et al., 2001; Chandran et al., 2014). Therefore, in many species, the high ratio of estrogen to progesterone in the maternal circulation is thought to contribute the onset of labor. Parturition is associated with an influx of inflammatory cells and release of pro-inflammatory cytokines, including interleukin (IL)-1β and tumor necrosis factor (TNF)-α, in the myometrium, cervix and fetal membranes (Golightly et al., 2011). In mice, progesterone reduces the expression of pro-inflammatory cytokines, including IL-1β and IL-6 by the uterus and trophoblast and may modulate the abundance of myometrial monocytes (Table 3; Estrogen; Edey et al., 2018). Progesterone also decreases the ability of LPS to induce pro-inflammatory cytokine secretion by human myometrium and placental explants (Youssef et al., 2009; Garcia-Ruíz et al., 2015). It also diminishes the ability of estrogen to induce the infiltration of macrophages and neutrophils into the uterus, and decreases LPS-induced leukocyte adhesion to human umbilical vein cells (Simoncini et al., 2004). Thus, it is perhaps not surprising that progesterone receptor null mice demonstrate chronic uterine inflammation, particularly in response to estrogen treatment (Table 3; Estrogen; Lydon et al., 1995). There is also evidence that placental steroids participate in cervical softening, by regulating the expression of matrix remodeling enzymes as well as leukocyte infiltration and function (Chinnathambi et al., 2014; Gopalakrishnan et al., 2016; Berkane et al., 2017). In addition to regulating the events leading to parturition, recent data suggest that during the course of pregnancy, both estrogen and progesterone contribute to the maternal tolerance of the fetus by modulating proliferation and cytokine expression of CD4 and CD8 T cells and enhancing the suppressive function of T-regulatory cells (Mao et al., 2010; Robinson and Klein, 2012; Lissauer et al., 2015).

Additionally, both estrogen and progesterone are key stimulators of mammary gland development. For instance, progesterone stimulates proliferation of mammary stem cells and mammary epithelium (Tables 3, 4; Progesterone; Joshi et al., 2010; Lee et al., 2013). In mice, deficiency of the progesterone receptor restricts mammary gland development, whereas exogenous progesterone induces ductal side branching and lobuloalveolar differentiation and development (Table 3; Progesterone; Plaut et al., 1999; Joshi et al., 2010). In addition, both estrogen and progesterone may have indirect effects on mammary gland development by regulating prolactin secretion from the pituitary gland (Rezaei et al., 2016).

Maternal behavior during and after birth are regulated by the steroid hormones. Estrogen stimulates maternal nurturing behavior in numerous species, including rats, mice, sheep and primates (Bridges, 2015). In particular, maternal care is induced by estrogen treatment, whereas the converse happens when ERα expression is suppressed; deficiency of ERα increases the latency to pup retrieval and reduces the length of time dams spend nursing and licking their pups (Table 3; Estrogen; Ribeiro et al., 2012). Findings from animal models suggest that progesterone plays a role in regulating anxiety and depression-related behavior. For instance, exogenous progesterone stimulates anti-anxiety and anti-depressive actions in mouse dams (Table 3; Progesterone; Koonce and Frye, 2013). In contrast, progesterone withdrawal increases these types of behaviors (Gulinello et al., 2002). Thus, placental-derived steroids may modulate several aspects of maternal physiology which are beneficial to both pregnancy and post-partum support of the offspring.

### Neuroactive hormones

One major target of placental hormones is the maternal brain and related neuroendocrine organs such as the hypothalamus and pituitary glands. These neuroendocrine effects enable the mother to respond and adapt accordingly to her environment, so as to mitigate the adverse effects of stress and maintain homeostasis (Voltolini and Petraglia, 2014). Neuroactive hormones also prepare and enable the future mother to adequately care for her young (Lévy, 2016). In addition to their impact on the maternal neuroendocrine system, these hormones have additional functions *in vivo* and *in vitro* functions as well, which are detailed in Tables 5, 6, respectively.

**Table 5:** Effects of neuropeptides *in vivo*.

**Hormones**	**Expression level**	***In vivo*** **effects**		**References**
Serotonin	Low	Non-pregnant	**Serotonin receptor knockout Htr3a** ^−/−^**(mouse):** ↔ glucose tolerance; GSIS; serotonin production and release; pancreatic β-cell mass	Ohara-Imaizumi et al., 2013
			**Dietary restriction of precursor – tryptophan/inhibitor of serotonin synthase or receptor/serotonin receptor knockout Htr2b**^−/−^**(mouse):** ↔ glucose tolerance	Kim et al., 2010
			**Serotonin transporter knockout SERT** ^−/−^**(mouse):** ↓ food intake; glucose and insulin tolerance; hepatic and white adipose tissue glucose uptake and insulin sensitivity (Akt signaling); estrus cyclicity; blood 17β-estradiol; brown adipose tissue mass; lipid droplet number; lipolysis (PGC1α, PPARα, and CPT1b); ovarian Cyp19a expression ↑ blood glucose; white adipose tissue mass; adipocyte size; lipid droplet area; lipogenesis (PPARγ, SREBP1c, Fabp4, LPL, HSL and ATGL); adipose inflammation (IL-6 and TNF-α)	Zha et al., 2017
			**Administration of selective serotonin-reuptake inhibitors (mouse):** ↓ glucose and insulin tolerance; blood 17β-estradiol; ovarian Cyp19a expression ↑ weight; adiposity; adipocyte size	Alenina et al., 2009; Kane et al., 2012
			**Serotonin synthesis pathway enzyme knockout Tph2**^−/−^**(mouse):** ↓ postnatal survival; heart rate; blood pressure; respiration; social interaction; blood IGF1 ↑ early growth restriction; aggression; repetitive and compulsive behaviors; daytime sleep	
		Pregnancy and lactation	**Serotonin receptor knockout Htr3a** ^−/−^**(mouse):** ↓ glucose tolerance; GSIS ↔ weight; pancreatic β cell mass expansion; serotonin production and release; litter size	Ohara-Imaizumi et al., 2013
			**Serotonin synthesis pathway enzyme Tph1**^−/−^**(mouse):** ↓ blood and mammary serotonin and PTHrP; blood calcium; osteoclast activity; mammary gland epithelial cell proliferation, calcium transporters and sonic hedgehog signaling ↑ blood glucose and insulin	Laporta et al., 2014a,b
			**Serotonin synthesis pathway enzyme Tph2**^−/−^**(mouse):** ↓ brain serotonin; pup retrieval; nest building; offspring survival and weaning weights; lactation; lactation-induced aggression ↑ pup killing	Angoa-Pérez et al., 2014
			**Dietary restriction of precursor – tryptophan /inhibitor of serotonin synthase or receptor/serotonin receptor knockout Htr2b**^−/−^**(mouse):** ↓ glucose tolerance; pancreatic β-cell expansion (proliferation); blood insulin ↔ insulin tolerance	Kim et al., 2010
			**Serotonin transporter SERT** ^−/−^**(mouse):** ↑ blood glucose and insulin; JZ necrosis (TUNEL positive cells) and hemorrhage (fibrin deposition)	Hadden et al., 2017
	High	Non-pregnant	**No known physiological changes**	
		Pregnancy and lactation	**Infusion of serotonin precursor (cow):** ↓ food intake; colostrum yield; urine calcium elimination ↑ blood FFAs, calcium content; colostrum serotonin; loose stools; defecation frequency; urine metabolite (deoxypyridinoline); milk calcium content; hepatic expression of serotonin; hepatic CASP3- and Ki67-positive cell numbers ↔ blood glucose, insulin, magnesium, prolactin, glucagon; weight; milk yields; heart rates; respiration rates; body temperatures	Laporta et al., 2015; Weaver et al., 2016, 2017; Hernández-Castellano et al., 2017
			**Injection of precursor – tryptophan (mouse, rat and rabbit):** ↓ uterine blood flow; decidualization ↑ termination of pregnancy; placental hemorrhage; circulating PRL ↔ uterine contractility; serum progesterone	Poulson et al., 1960; Robson and Sullivan, 1966; Habiger, 1975 Mitchell et al., 1983; Tomogane et al., 1992
			**Dietary intake of precursor – tryptophan (mice, rats):** ↓ blood insulin; milk glucose ↑ blood, liver and mammary gland serotonin; blood and mammary gland PTHrP; blood and milk calcium; liver expression of gluconeogenic and glycolytic enzymes (PC, PCK, PDK4, PFK1); mammary gland expression of TPH1, calcium transporters, glucose transporters; femur bone resorption ↔ body weight; mammary gland structure and milk yield; pup weights	Laporta et al., 2013a,b
Melatonin	Low	Non-pregnant	**Melatonin receptor knockout MT1**^−/−^**(mouse):** ↓ glucose and insulin tolerance; circadian rhythm of blood glucose and corticosterone; time spent resting ↑ depressive-like and anxiety-like behaviors; psychomotor disturbances; time spent eating; hyperactivity; blood corticosterone and glucose; pancreatic insulin production; liver glucagon receptor expression	Weil et al., 2006; Contreras-Alcantara et al., 2010; Adamah-Biassi et al., 2014; Comai et al., 2015; Owino et al., 2016
			**Melatonin receptor knockout MT2**^−/−^**(mouse):** ↓ circadian rhythm of blood glucose; blood insulin; axon formation; synaptic transmission ↑ liver glucagon receptor expression; pancreatic insulin production	Liu et al., 2015
			**Double melatonin receptor knockout MT1/MT2**^−/−^**(mouse):** ↓ blood insulin ↑ cognitive performance; hyperactivity; motor activity; liver glucagon receptor expression; pancreatic insulin production	Mühlbauer et al., 2009; Bähr et al., 2011; O'neal-Moffitt et al., 2014
		Pregnancy and lactation	**No known physiological changes**	
	High	Non-pregnant	**Exogenous (rat):** ↓ liver glucagon receptor expression ↑ blood glucagon	Bähr et al., 2011
			**Mammary-specific melatonin MT1 receptor overexpression (mouse):** ↓ mammary gland ductal growth, ductal branching, and terminal end bud formation	Xiang et al., 2012
		Pregnancy and lactation	**Exogenous (cow):** ↑ heart rate; pulse pressure; uterine blood flow; uterine melatonin receptor expression ↓ circulating progesterone and estradiol ↔ gestation and birthweight	Brockus et al., 2016
			**Exogenous (sheep):** ↓ pancreatic insulin-positive tissue area, size and percentage of large insulin-containing cell clusters; blood prolactin receptors; milk protein content (β-casein and whey acidic protein) ↑ oxygen consumption; blood LH and progesterone; pancreas and small intestine weights; pancreatic α-amylase activity; citrate synthase activity; number of fetuses; conception and pregnancy rates	Wallace et al., 1988; Denicolo et al., 2008; Scott et al., 2009; Prezotto et al., 2014; Keomanivong et al., 2016
			**Exogenous in growth restriction model – high altitude (sheep):** ↓ oxidative stress (↓ blood 8-isoprostanes); birthweight ↑ blood cortisol; plasma antioxidant capacity; gestation length	González-Candia et al., 2016
			**Exogenous (rat):** ↓ food intake; weight gain; blood and pituitary LH; pituitary prolactin; litter size; birthweight ↑ blood prolactin; offspring mortality	Nir and Hirschmann, 1980; Jahnke et al., 1999; Singh et al., 2013
			**Melatonin receptor MT1 overexpression (mouse):** ↓ mammary gland lobulo-alveolar development; mammary epithelial cell proliferation (Akt1, phospho-Stat5, Wnt4) and estrogen and progesterone receptor expression; suckling pup weight	Xiang et al., 2012
Oxytocin	Low	Non-pregnant	**Oxytocin knockout OT**^−/−^**(mouse):** ↓ glucose and insulin tolerance; bone mineral density; social memory; maternal behavior (pup retrieval and licking) ↑ adiposity; sucrose solution intake; carbohydrate preference; blood glucose, leptin and adrenaline ↔ food intake	Ferguson et al., 2000; Amico et al., 2005; Pedersen et al., 2006; Miedlar et al., 2007; Sclafani et al., 2007; Camerino, 2009; Tamma et al., 2009
			**Oxytocin receptor knockout OTR** ^−/−^**(mouse):** ↓ bone mineral density; cold-induced thermogenesis; social memory; maternal behavior (pup retrieval) ↑ adiposity; aggressive behavior; blood triglycerides; brown adipose tissue lipid droplet size	Takayanagi et al., 2005, 2008; Lee et al., 2008; Tamma et al., 2009
			**Oxytocin antagonist administration (rat):** ↓ latency to first meal post-fast ↑ food and fluid intake; time spent eating	Arletti et al., 1989, 1990
			**OXTR RNAi administration (prairie voles):** ↓ social attachment; maternal care (grooming)	Keebaugh et al., 2015
		Pregnancy and lactation	**Oxytocin knockout OT** ^−/−^**(mouse):** ↓ milk release; post-partum mammary development ↑ mammary gland milk accumulation	Nishimori et al., 1996; Young et al., 1996; Wagner et al., 1997
			**Oxytocin receptor knockout OTR** ^−/−^**(mouse):** ↓ milk release; maternal behavior (pup retrieval) ↑mammary gland milk accumulation	Takayanagi et al., 2005; Lee et al., 2008
			**Oxytocin antagonist administration (rat):** ↑ latency to display maternal behaviors (nest building, pup retrieval)	Van Leengoed et al., 1987
	High	Non-pregnant	**Exogenous (rat):** ↓ food and fluid intake; blood pressure; blood calcium ↑ latency to first meal post-fast; bone formation	Arletti et al., 1989, 1990; Petersson et al., 1996; Elabd et al., 2007
			**Exogenous (diet-induced obese rats):** ↓ weight gain ↑ glucose and insulin tolerance; adipose tissue lipolysis and fatty acid β-oxidation	Deblon et al., 2011
			**Exogenous (mouse):** ↑ body temperature; bone mineral density	Mason et al., 1986; Tamma et al., 2009
		Pregnancy and lactation	**Exogenous (rat):** ↑ delivery induction (via induced Fos expression in supraoptic nucleus and brain stem neurons)	Antonijevic et al., 1995
			**Injection of oxytocin antagonist (Syrian hamster):** ↑ aggression to intruder (number of bites and contact time)	Ferris et al., 1992

*ATGL, Adipose triglyceride lipase; CASP, Caspase; CPT1b, Carnitine palmitoyltransferase 1B; GSIS, Glucose-stimulated insulin secretion; HSL, Hormone-sensitive lipase; IL, Interleukin; JZ, junctional zone; PC, Pyruvate carboxylase; PDK4, Pyruvate dehydrogenase kinase 4; PFK1, 6-phosphofructokinase subunit alpha; PGC1, PPARG Coactivator 1; PPAR, Peroxisome proliferator-activated receptor; SREBP1, Sterol regulatory element-binding transcription factor 1; LPL, Lipoprotein lipase; TNF, Tumor necrosis factor*.

**Table 6:** Effects of neuropeptides *in vitro*.

**Hormones**	**Expression level**	***In vitro* effects**	**References**
Serotonin	Low	**Exposure to selective serotonin-reuptake inhibitors (BeWo trophoblast cell and H295R adrenocortical cell co-culture):** ↓ serotonin transporter activity; estrogen secretion ↑ aromatase CYP19 activity	Hudon Thibeault et al., 2017
	High	**Exogenous (human third trimester placental arteries and veins):** ↑ vessel vasoconstriction; cotyledon; perfusion pressure and thromboxane release	Bjøro and Stray-Pedersen, 1986; Cruz et al., 1997
		**Exogenous (bovine placentome cells):** ↑ proliferation	Fecteau and Eiler, 2001
		**Exogenous (human adipocytes):** ↑ lipid-binding proteins, glucose carriers, triacylglycerol synthesis enzymes	Sonier et al., 2005
		**Exogenous (mouse adipocytes):** ↓ brown fat differentiation ↑ fat storage and white fat differentiation; lipid-binding proteins, glucose carriers, triacylglycerol synthesis enzymes	Grès et al., 2013; Rozenblit-Susan et al., 2017
		**Exogenous (mouse pancreatic** β **cells):** ↑ proliferation	Kim et al., 2010
		**Exogenous (rat osteoblast):** ↓ proliferation; differentiation; mineralization	Dai et al., 2014
Melatonin	Low	**Melatonin receptor MT1 siRNA administration (rat insulinoma):** ↑ insulin production and secretion	Wang et al., 2017
	High	**Exogenous (human trophoblast cells):** ↑ hCG secretion; syncytialization ↓ hypoxia-induced oxidative stress and apoptosis; mitochondrial lipid peroxidation	Iwasaki et al., 2005; Milczarek et al., 2010; Lanoix et al., 2013; Soliman et al., 2015
		**Exogenous (human myometrial cells):** ↑ oxytocin-induced contractility; oxytocin sensitization	Ayar et al., 2001; Sharkey et al., 2009, 2010
		**Exogenous (rat myometrial cells):** ↓ spontaneous and oxytocin-induced contractility	Abd-Allah et al., 2003
		**Exogenous (rat uterine and hypothalamic explants):** ↓ prostaglandin release	Gimeno et al., 1980
		**Exogenous (seal uterine artery):** ↓ noradrenaline-induced vasoconstriction	Stokkan and Aarseth, 2004
		**Exogenous (rat insulinoma and mouse pancreatic islets):** ↓ insulin release; expression of glucagon-like peptide 1; glucagon-stimulated insulin release	Mühlbauer et al., 2012
		**Exogenous (mouse pancreatic** α**-cells):** ↑ glucagon production	Bähr et al., 2011
Oxytocin	Low	**Oxytocin knockout OT**^−/−^**(mouse osteoblast and osteoclast cells):** ↓ proliferation; maturation; differentiation	Tamma et al., 2009
	High	**Exogenous (human third trimester primary trophoblast cells):** ↓ nitric oxide production	Nanetti et al., 2015
		**Exogenous (human decidual cells in labor):** ↑ prostaglandin synthesis; release of free arachidonic acid	Wilson et al., 1988
		**Exogenous (guinea pig placenta perfusion):** ↓ uptake of glucose and alanine (related to changes in placental flow)	Rybakowski et al., 2000
		**Exogenous (rat myometrial strips):** ↑ contractility	Ayar et al., 2001
		**Exogenous (rat mammary gland slice):** ↑ release of triglycerides and protein	Da Costa et al., 1995
		**Exogenous (human umbilical vein endothelial cells):** ↑ migration; invasion	Cattaneo et al., 2008
		**Exogenous (mouse osteoblast and osteoclast):** ↑ proliferation; differentiation	Tamma et al., 2009

#### Melatonin and serotonin

Melatonin and its precursor, serotonin, are tryptophan-derived hormones with well-known neuroendocrine impacts. In humans, circulating concentrations of melatonin and serotonin increase as pregnancy advances (Lin et al., 1996; Nakamura et al., 2001). In the non-pregnant state, melatonin and serotonin are primarily produced by the pineal gland and the brain, respectively. However, the enzymes involved in melatonin and serotonin biosynthesis are also expressed by the human placenta throughout gestation (Iwasaki et al., 2005; Soliman et al., 2015; Laurent et al., 2017). The mouse placenta similarly expresses the enzymes needed for serotonin synthesis (Wu et al., 2016), although work is required to assess if melatonin synthesizing enzymes are also expressed. The rat placenta does not produce melatonin *de novo* due to the lack of synthesizing enzymes (Tamura et al., 2008). However, the same study demonstrated that conditioned medium from cultured term rat placentas stimulated melatonin release by the maternal pineal gland (Tamura et al., 2008). These findings suggest that placental-derived factors may indirectly regulate melatonin levels by the mother during pregnancy. Placental expression of melatonin, serotonin and their respective enzymes, also remains to be investigated in other species such as rabbits and sheep, which are commonly used in pregnancy-related studies. Mouse models that result in deficiencies or reduced bioactivity of these hormones demonstrate altered sleep patterns, melancholic behavior, hyperactivity and aggression in the non-pregnant state (Table 5; Serotonin and Melatonin; Weil et al., 2006; Alenina et al., 2009; Kane et al., 2012; Adamah-Biassi et al., 2014; O'neal-Moffitt et al., 2014; Comai et al., 2015). Serotonin is thus a major regulator of maternal mood and behavior (Angoa-Pérez and Kuhn, 2015). For instance, genetically-induced serotonin deficiency leads to increased maternal aggression, lower pup retrieval and greater pup cannibalization, which reduces postnatal survival of offspring in mice (Angoa-Pérez et al., 2014). There is some evidence that serotonin and melatonin may also impact maternal feeding behavior. For example, increased serotonin signaling reduces food intake in pregnant cows (Laporta et al., 2015; Weaver et al., 2016, 2017; Hernández-Castellano et al., 2017). Similarly, exogenous melatonin lowers food intake in pregnant rats (Nir and Hirschmann, 1980; Jahnke et al., 1999; Singh et al., 2013). These negative effects on maternal food intake suggest that peak serotonin and melatonin concentrations in late pregnancy may serve to control the maternal appetite and prevent excessive weight gain.

Another key function of melatonin and serotonin is glucose homeostasis and the regulation of steroid synthesis (Table 5; Serotonin and Melatonin). In mice, loss of melatonin or serotonin signaling leads to glucose intolerance and insulin resistance, with consequences for blood glucose and insulin concentrations in both the non-pregnant and pregnant state (Contreras-Alcantara et al., 2010; Kim et al., 2010; Owino et al., 2016). However, these neuroactive hormones appear to have differential effects on the pancreas (Table 6; Serotonin and Melatonin). Serotonin promotes pancreatic β-cell proliferation *in vitro* (Kim et al., 2010), and is thus important for pancreatic β-cell mass expansion during pregnancy in mice (Goyvaerts et al., 2016). In contrast, melatonin reduces insulin release by rodent pancreatic islets *in vitro* (Mühlbauer et al., 2012). Non-pregnant mice with deficient serotonin signaling have impaired lipid handling and excessive lipid accumulation in association with reduced adipose aromatase expression and circulating estrogen (Zha et al., 2017). Similarly, treating placental-derived trophoblast cells with norfluoxetine, a selective serotonin-reuptake inhibitor, inhibits aromatase activity and estrogen secretion *in vitro* (Hudon Thibeault et al., 2017). Supplementation of melatonin in non-pregnant humans reduces circulating triglycerides and cholesterol levels, but effects of lipid handling in pregnancy are unknown (Mohammadi-Sartang et al., 2017). Melatonin also modulates steroid production. For instance, melatonin treatment in pregnant cows reduces circulating estrogen and progesterone (Brockus et al., 2016), while lack of melatonin signaling raises blood corticosterone in mice (Comai et al., 2015).

Given melatonin's additional effects on regulating the circadian rhythm (Mühlbauer et al., 2009), there is some weak evidence for its role in the timing of parturition (Yellon and Longo, 1988; González-Candia et al., 2016). Melatonin can either enhance or reduce uterine myometrial contractility depending on the species (Table 6; Melatonin; Ayar et al., 2001; Sharkey et al., 2009, 2010). Both melatonin and serotonin are also important for lactation, specifically for mammary gland development and milk nutrient content (Okatani et al., 2001; Xiang et al., 2012; Laporta et al., 2014a,b). For instance, mammary gland proliferation and calcium transport is impaired in pregnant mice with genetically-induced serotonin deficiency (Laporta et al., 2014a,b). Conversely, supplementation of a serotonin precursor increases mammary calcium transporter expression and milk calcium content in lactating mice and cows (Laporta et al., 2013a,b, 2015; Weaver et al., 2016, 2017; Hernández-Castellano et al., 2017). In contrast to serotonin, increased melatonin signaling is associated with reduced ductal growth and branching, as well as impaired terminal end bud formation in the non-pregnant state (Xiang et al., 2012). Thus, during lactation, these mice with increased melatonin signaling have impaired mammary gland lobulo-alveolar development and reduced milk protein content, which reduces the weight of suckling pups (Xiang et al., 2012). Indeed, a recent study showed antenatal melatonin supplementation further exacerbated the growth restriction of offspring and raised circulating maternal cortisol in a sheep model of fetal growth restriction (González-Candia et al., 2016). Nevertheless, melatonin supplementation during pregnancy confers significant beneficial neuroprotective effects on the fetus and enhances maternal antioxidant capacity (Miller et al., 2014; González-Candia et al., 2016; Castillo-Melendez et al., 2017). Therefore, while melatonin supplementation shows promise for use in the clinic, particularly for enhancing the neurodevelopmental outcomes of offspring in growth compromised pregnancies, the potential adverse outcomes for both mother and child must also be considered and should be assessed in further studies.

#### Oxytocin

Another key neuroendocrine factor is oxytocin. Oxytocin is widely known for its role in triggering maternal nursing behavior (Bosch and Neumann, 2012). This is mediated by oxytocin's actions on the maternal brain, as well as, the mammary glands. Indeed, a greater rise in circulating oxytocin concentrations from early to late pregnancy in pregnant women, is associated with a stronger bond between a mother and her infant (Levine et al., 2007). Concurrently, placental expression of oxytocin also peaks at term in humans (Kim S. C. et al., 2017). The rat placenta also produces oxytocin (Lefebvre et al., 1992), while placental expression in other species remains unclear. Reduced oxytocin signaling decreases maternal nurturing behavior such as pup retrieval in rats (Van Leengoed et al., 1987). It also decreases the willingness of female voles to care for, groom and lick unrelated pups (Keebaugh et al., 2015). Low oxytocin signaling can additionally impair social bonding in voles and mice (Ferguson et al., 2000; Takayanagi et al., 2005; Lee et al., 2008; Keebaugh et al., 2015), while high levels builds trust and cooperation in a group setting to facilitate group survival in humans (Declerck et al., 2010; De Dreu et al., 2010). Moreover, a lack of oxytocin disrupts mammary gland proliferation and lobuloalveolar development, which impairs milk release from the mammary tissues in mice (Nishimori et al., 1996; Wagner et al., 1997). Therefore, high oxytocin levels enable the mother to bond better and protect her newborn, when it is most vulnerable.

Oxytocin is also important in the process of parturition (Table 6; Oxytocin); it stimulates the contraction of smooth muscle cells in the myometrium (Ayar et al., 2001; Arrowsmith and Wray, 2014), by inducing calcium influx and stimulating prostaglandin release (Wilson et al., 1988; Voltolini and Petraglia, 2014; Kim S. H. et al., 2017). Cardiovascular effects of oxytocin include its ability to significantly lower blood pressure in non-pregnant rats (Petersson et al., 1996). There is also some evidence that oxytocin induces anti-inflammatory and antioxidant effects in the heart under hypoxic conditions in non-pregnant rats (Gutkowska and Jankowski, 2012). Nevertheless, the specific cardiovascular effects of oxytocin in pregnancy remain to be explored.

Studies performed in non-pregnant rodents show that oxytocin also affects metabolic function *in vivo* (Table 5; Oxytocin). In particular, loss of oxytocin reduces glucose and insulin tolerance and increases adiposity (Camerino, 2009), whereas exogenous oxytocin has the reverse effect (Deblon et al., 2011). Studies are however, required to determine whether the rise in oxytocin in late pregnancy (Levine et al., 2007) may serve to improve insulin sensitivity in the mother in preparation for the metabolic requirements of delivery and lactation. There is some evidence that oxytocin may additionally play a role in controlling energy expenditure and thermoregulation during pregnancy. Even with a similar diet and activity level to control mice, oxytocin-deficient mice become obese due to reduced energy expenditure from poor thermoregulation in the non-pregnant state (Chaves et al., 2013). Furthermore, exogenous oxytocin in non-pregnant mice causes a rise in body temperature (Mason et al., 1986; Tamma et al., 2009). Nevertheless, whether oxytocin may play a role in controlling heat dissipation due to the increased maternal energy expenditure during pregnancy requires exploration. Exogenous oxytocin also reduces food intake in non-pregnant rats (Arletti et al., 1989, 1990). However, the role of oxytocin in appetite regulation during pregnancy remains to be explored. There is also evidence for oxytocin's possible involvement in maternal bone metabolism and calcium homeostasis during pregnancy and lactation. For instance, oxytocin stimulates both bone resorption and bone formation by osteoclasts and osteoblasts respectively *in vitro* (Tamma et al., 2009). Moreover, oxytocin administration in rats reduces circulating calcium with an overall skew toward bone formation (Elabd et al., 2007). These findings may suggest that the peak in circulating oxytocin toward term promote the restoration of depleted maternal skeletal calcium stores.

#### Other neuroactive hormones

In addition to the aforementioned melatonin, serotonin and oxytocin, the human placenta also produces neuroactive hormones such as kisspeptin and thyrotropin-releasing hormone (TRH), which may function in adapting maternal physiology to support pregnancy (Bajoria and Babawale, 1998; De Pedro et al., 2015). In humans, circulating kisspeptin rises throughout pregnancy to concentrations 10,000-fold that of the non-pregnant state, with the placenta speculated as a major source (Horikoshi et al., 2003). In the non-pregnant state, kisspeptin can both stimulate and impede glucose stimulated insulin secretion in mice (Bowe et al., 2009; Song et al., 2014). The nature of the effect may partly relate to differences in the actions of kisspeptin isoforms on pancreatic islets (Bowe et al., 2012). Kisspeptin may also have effects on the maternal cardiovascular system, given its reported vasoconstrictive effects on vascular smooth muscle cells and fibrotic effects on the heart in non-pregnant rats (Mead et al., 2007; Zhang et al., 2017). Studies in humans highlight the importance of regulating kisspeptin production during gestation; increased placental kisspeptin is associated with pre-eclampsia (Whitehead et al., 2013; Matjila et al., 2016) and reduced circulating kisspeptin is observed in women with hypertension and diabetes during pregnancy (Cetković et al., 2012; Matjila et al., 2016). Like the human, the murine placenta produces kisspeptin. Although a kisspeptin-deficient mouse has been established, previous work has been focused on feto-placental outcomes, with no examination of maternal physiology (Herreboudt et al., 2015). Studies are required to determine the consequences of abnormal placental kisspeptin on the maternal physiology during pregnancy.

In the non-pregnant state, hypothalamic TRH stimulates release of thyroid-stimulating hormone and PRL from the pituitary (Hershman et al., 1973; Vale et al., 1973; Askew and Ramsden, 1984). However, during pregnancy, the placenta serves as an additional source of TRH (Bajoria and Babawale, 1998). Excess TRH in pregnancy raises blood concentrations of thyroid-stimulating hormone and PRL in humans, rhesus monkeys, sheep and rats (Thomas et al., 1975; Azukizawa et al., 1976; Roti et al., 1981; Moya et al., 1986; Lu et al., 1998). Conversely, a lack of TRH reduces blood PRL in mice (Rabeler et al., 2004; Yamada et al., 2006). Thyroid hormones are necessary for optimal brain development as well as thyroid function (Miranda and Sousa, 2018). Impaired TRH signaling is associated with anxiety-like and depressive-like behavior in non-pregnant mice (Zeng et al., 2007; Sun et al., 2009) and there is some evidence which suggests a link between thyroid dysfunction and poor maternal mood during pregnancy in humans (Basraon and Costantine, 2011). However, whether any direct causal relationship between placental hormones, like TRH and perinatal depression remains unclear. Additionally, TRH is implicated in glucose homeostasis and appetite regulation. For example, mice with TRH deficiency are hyperglycaemic, due to an impaired insulin response to glucose (Yamada et al., 1997). Reduced TRH signaling also impedes leptin production and ghrelin acylation, which results in less energy conservation during fasting and a lower body mass in the non-pregnant state (Groba et al., 2013; Mayerl et al., 2015). Investigations are warranted to identify whether TRH may contribute to the regulation of glucose handling and appetite in the mother during pregnancy.

### Additional hormones

The placenta also produces numerous other hormones with pleiotropic effects. Several key ones, which have been implicated in pregnancy failure or disorders of pregnancy such as hypertension, hyperglycemia and hypercalcemia, are discussed here. The hormones presented here are by no means exhaustive and were selected primarily on their major associations with abnormal maternal physiology during pregnancy. The gonadotropin, chorionic gonadotropin (CG); transforming growth factor β (TGF β) family member, activin; angiogenic factor, relaxin; bone metabolism-associated parathyroid hormone-related protein (PTHrP) and energy homeostasis regulator, leptin are reviewed (Tables 7, 8).

**Table 7:** Effects of additional hormones *in vivo*.

**Hormones**	**Expression levels**	***In vivo*** **effects**		**References**
Activins	Low	Non-pregnant	**Dysfunctional activin receptor ACVR1C (mouse):** ↓ fat accumulation ↑ adipocyte lipolysis	Yogosawa et al., 2013
			**Truncated activin receptor ACVR2A (mouse):** ↑ number and area of renal glomeruli ↓ size of renal glomeruli	Maeshima et al., 2000
			**Bone-specific activin receptors ACVR2A and/or ACVR2B deletion (mouse):** ↑ femoral trabecular bone volume	Goh et al., 2017
		Pregnancy and lactation	**No known physiological effects**	
	High	Non-pregnant	**Induced endogenous overexpression (mouse):** ↑ estrus stage in cycle; blood activin A and FSH; numbers of corpora lutea; granulosa cell layer thickness; ovary size	Kim et al., 2008
			**Exogenous (mouse):** ↑ activation of muscle catabolic pathways	Ding et al., 2017
		Pregnancy and lactation	**Exogenous (mouse):** ↓ gestation length ↑ blood pressure; proteinuria; endothelial oxidative stress; fetal growth restriction	Lim et al., 2015
PTHrP	Low	Non-pregnant	**PTHRP knockout PTHrP** ^−/−^**(mouse):** ↓ height; chondrocyte proliferation ↑ premature chondrocyte maturation; bone mineralization - Lethal at birth	Karaplis et al., 1994
		Pregnancy and lactation	**Infusion of PTH/PTHrP receptor antagonist or antibody against PTHrP:** ↓ decidual apotosis ↑ decidualization; uterine weight	Vanhouten et al., 2003
			**Mammary-specific PTHrP deletion (mouse):** ↓ blood and milk PTHrP; blood vitamin D; urinary cAMP; bone turnover; lactation-associated bone loss ↑ bone mass	Williams et al., 1998
			**Bone-specific PTHrP deletion (mouse):** ↑ skeletal fragility	Kirby et al., 2011
	High	Non-pregnant	**Mammary-specific PTHrP overexpression (mouse):** ↓ mammary ductal branching and elongation	Wysolmerski et al., 1995; Dunbar et al., 2001
			**Pancreatic** β **cell-specific PTHrP overexpression (mouse):** ↓ diabetogenic effects of streptozotocin; blood glucose ↑ pancreatic islet number and mass; circulating insulin	Vasavada et al., 1996; Porter et al., 1998
			**Bone-specific PTHrP overexpression/constitutively active PTHrP receptor (mouse):** ↓ bone ossification, mineralization and length; chondrocyte differentiation	Weir et al., 1996; Schipani et al., 1997
			**Kidney-specific PTHrP overexpression (mouse):** ↑ renal hypertrophy; urinary albumin excretion	Izquierdo et al., 2006; Romero et al., 2010
		Pregnancy and lactation	**Exogenous (goat):** ↑ mammary gland uptake of calcium, phosphorous, magnesium; milk calcium, phosphorous, magnesium content	Barlet et al., 1992
			**Mammary-specific PTHrP overexpression (mouse):** ↓ mammary lobuloalveolar and terminal duct development	Wysolmerski et al., 1995
Relaxin	Low	Non-pregnant	**Relaxin knockout Rln**^−/−^**(mouse):** ↓ renal smooth muscle cell density ↑ mean arterial pressure; lung function (airway fibrosis and smooth muscle thickening); heart weight (expression of cardiac hypertrophy associated genes); renal collagen content	Samuel et al., 2003; Lekgabe et al., 2006; Debrah et al., 2011; Mirabito Colafella et al., 2017
		Pregnancy and lactation	**Relaxin knockout Rln**^−/−^**(mouse):** ↓ gestational weight gain; lactation; blood sFlt-1; mammary gland development; reproductive tissue growth and remodeling (e.g., cervix, vagina); litter size ↑ labor length; mean arterial pressure; plasma osmolality; urinary albumin/creatinine ratio; vascular vasoconstriction; expression of angiogenic markers (Vegfa, Esr1, Pgr, Rxfp1, Egln1, Hif1a, MMP14, Ankrd37); blood progesterone; mammary duct dilation	Zhao et al., 1999, 2000; Marshall et al., 2016a,b; Mirabito Colafella et al., 2017; O'sullivan et al., 2017
			**Relaxin receptor knockout RXFP1** ^−/−^**(mouse):** ↓ mammary gland development; lactation ↑ obstructed delivery; lung fibrosis and collagen accumulation	Kamat et al., 2004; Krajnc-Franken et al., 2004
			**Smooth muscle-specific relaxin receptor RXFP1 deletion (mouse):** ↓ cervival and vaginal epithelial development ↑ collagen content in reproductive tract organs and uterine artery	Kaftanovskaya et al., 2015
			**Administration of relaxin antibody (rat):** ↓ stroke volume; cardiac output; global arterial compliance ↑ systemic vascular resistance	Debrah et al., 2006
	High	Non-pregnant	**Exogenous (rhesus monkeys):** ↓ endometrial expression of MMP1 and MMP3; endometrial progesterone production ↑ blood GH and prolactin; endometrial growth; endometrial angiogenesis (endothelial proliferation and dilatation); uterine weight; endometrial expression of TIMP1, estrogen receptor alpha; endometrial resident lymphocyte number	Hisaw et al., 1967; Bethea et al., 1989; Goldsmith et al., 2004
			**Exogenous (rat):** ↓ systemic and renal vascular resistance; angiotensin-induced renal vasoconstriction; plasma osmolality; haematocrit; vascular smooth muscle tone ↑ renal plasma flow; glomerular filtration rate; urinary sodium excretion; water intake; cardiac output; global arterial compliance; uterine artery blood flow velocity	Weisinger et al., 1993; Danielson et al., 1999; Conrad et al., 2004; Vodstrcil et al., 2012
			**Exogenous (mouse):** ↓ cervical and vaginal apoptosis of stroma and epithelium; renal collagen content ↑ decidualization; decidual expression of laminin; cervical and vaginal proliferation of stroma and epithelium; renal vascular remodeling; renal smooth muscle cell density	Bani et al., 1995; Yao et al., 2008; Debrah et al., 2011
			**Overexpression (mouse):** ↑ nipple hypertrophy	Feng et al., 2006
		Pregnancy and lactation	**Exogenous (rhesus monkeys):** ↑ blood prolactin	Bethea et al., 1989
			**Exogenous (marmoset):** ↓ gestation length ↑ uterine expression of estrogen-associated factors; uterine macrophage infiltration; endometrial angiogenesis; uterine growth; placental growth	Einspanier et al., 2009
Leptin	Low	Non-pregnant	**Dysfunctional leptin Lep**^ob/ob^ **(mouse):** ↓ activity; oxygen consumption; body temperature ↑ food intake; weight; weight gain; adiposity; blood glucose and insulin	Pelleymounter et al., 1995
			**Heterozygous for dysfunctional leptin Lep**^ob/+^ **or leptin receptor Lepr**^db/+^ **(mouse):** ↑ adiposity; adipose tissue mass	Chung et al., 1998
		Pregnancy and lactation	**Dysfunctional leptin Lep**^ob/ob^ **(mouse) with pre to mid pregnancy leptin treatment to initiate pregnancy** ↓ lactation; mammary gland development ↑ food intake; gestation length	Chehab et al., 1996; Mounzih et al., 1998; Malik et al., 2001
			**Heterozygous for dysfunctional leptin receptor Lepr**^db/+^ **(mouse):** ↓ glucose and insulin tolerance; skeletal muscle insulin signaling ↑ food intake; weight gain; GSIS; blood leptin; fasting blood glucose; adipose tissue mass; hepatic glucose production; fetal weight ↔ fed and fasting blood insulin	Ishizuka et al., 1999; Yamashita et al., 2001
	High	Non-pregnant	**Exogenous (rat):** ↓ food intake; blood glucose and insulin ↑ blood pressure; heart rate; oxygen consumption; energy expenditure (brown adipose thermogenesis)	Scarpace et al., 1997; Shek et al., 1998
			**Overexpression (mouse):** ↓ time to puberty and menopause onset; liver; white and brown adipose tissue mass; hepatic glycogen and lipid storage ↑ glucose metabolism; insulin sensitivity (skeletal muscle and hepatic insulin signaling); blood pressure; sympathetic nervous system activation; urinary catecholamine content	Ogawa et al., 1999; Aizawa-Abe et al., 2000; Yura et al., 2000
			**Exogenous (mouse):** ↓ food intake; weight; weight gain; time to puberty onset; blood LH ↑ lean mass percentage; ovarian and uterine weight	Pelleymounter et al., 1995; Chehab et al., 1997
		Pregnancy and lactation	**Overexpression (mouse):** ↓ food intake; fetal weight ↑ blood pressure; pregnancy-associated rise in blood leptin	Sagawa et al., 2002
			**Exogenous (mouse):** ↓ food intake; weight gain; GSIS; fed blood insulin; fasting blood insulin and leptin; adipose tissue mass; fetal and placental weights; placental leptin ↑ fed blood glucose	Kulkarni et al., 1997; Yamashita et al., 2001
			**Exogenous (rat):** ↑ blood pressure; proteinuria; blood markers of endothelial activation (E-selectin and ICAM-1) ↔ food intake; weight	Ibrahim et al., 2013

*cAMP, Cyclic adenosine monophosphate; FSH, Follicle stimulating hormone; GSIS, Glucose-stimulated insulin secretion; ICAM-1, Intercellular adhesion molecule 1; LH, Luteinizing hormone; MMP, Matrix metalloproteinase; TIMP, Tissue inhibitor of metalloproteinase*.

**Table 8:** Effects of additional hormones *in vitro*.

**Hormones**	**Expression level**	***In vitro* effects**	**References**
Activins	Low	**Exogenous low physiological concentrations (human endothelial cells):** ↑ proliferation and migration	Yong et al., 2015
		**Activin receptor ACVR2A siRNA knockdown (human endometrial stromal cells):** ↓ decidualization	Yong et al., 2017
		**Activin receptor knockout ACVR2A**^−/−^**(mouse osteoblast cells):** ↑ differentiation; mineral deposition; expression of osterix, osteocalcin, and dentin matrix acidic phosphoprotein 1	Clementi et al., 2013; Goh et al., 2017
	High	**Exogenous (human first trimester and third trimester primary trophoblast, JEG-3 and HTR-8/SVneo cells):** ↓ inhibin secretion ↑ apoptosis; invasion (SNAIL, SLUG, MMP2); hCG production; oxytocin secretion; aromatase activity (estrogen production); progesterone production	Qu and Thomas, 1993; Steele et al., 1993; Florio et al., 1996; Song et al., 1996; Ni et al., 2000; Bearfield et al., 2005; Jones et al., 2006; Yu et al., 2012; Li et al., 2014, 2015
		**Exogenous (mouse placental cells):** ↑ differentiation to labyrinth cell fate ↓ growth hormone releasing hormone secretion	Yamaguchi et al., 1995
		**Exogenous (rat decidual stromal cells):** ↑ apoptosis (DNA degradation; caspase 3 activity)	Tessier et al., 2003
		**Exogenous (human endometrial stromal cells):** ↑ decidualisation; production of MMP2, MMP3, MMP7, MMP9	Jones et al., 2006
		**Exogenous high pathological concentrations (human endothelial cells):** ↑ oxidative stress, permeability and endothelin production	Lim et al., 2015; Yong et al., 2015
		**Exogenous (mouse myoblast cells):** ↑ atropy; myofibrillar protein loss; autophagy activation	Ding et al., 2017
*PTHrP*	Low	**Parathyroid hormone-related protein knockout PTHrP** ^−/−^**(mouse ectoplacental cone explant):** ↑ apoptosis ↓ proliferation; differentiation	Duval et al., 2017
		**PTHrP antibody, siRNA or receptor antagonist administration (rat and mouse vascular smooth muscle cells):** ↓ proliferation ↑ PTH1R expression	Song et al., 2009
		**PTHrP antibody or siRNA administration (mouse podocytes):** ↓ high glucose induced hypertrophy	Romero et al., 2010
	High	**Exogenous (human third trimester cytotrophoblast cells):** ↓ apoptosis	Crocker et al., 2002
		**Exogenous (rat choriocarcinoma cells):** ↑ calcium uptake	Hershberger and Tuan, 1998
		**Exogenous (mouse ectoplacental cone cells):** ↑ trophoblast giant cell differentiation	El-Hashash and Kimber, 2006
		**Exogenous (human, baboon and rat myometrium):** ↓ spontaneous contraction; oxytocin-induced contraction	et al., 1994; Pitera et al., 1998; Slattery et al., 2001
		**Exogenous (rat uterine artery):** ↑ relaxation	Meziani et al., 2005
		**Exogenous (mouse podocytes):** ↑ high glucose-induced hypertrophy	Romero et al., 2010
		**Exogenous (human lung epithelial cell):** ↓ proliferation ↑ surfactant production	Sasaki et al., 2000
		**Exogenous (mouse osteoblast):** ↑ growth arrest (↓cyclin D1 expression; CDK1 kinase activity)	Datta et al., 2005
		**Exogenous (rat and mouse vascular smooth muscle cells):** ↓ proliferation	Song et al., 2009
hCG	Low	**hCG antibody administration (human third trimester cytrophoblast cells):** ↓ syncytiotrophoblast differentiation	Shi et al., 1993
		**hCG receptor antibody administration (human third trimester cytrophoblast cells):** ↑ syncytiotrophoblast differentiation; hCG release (*autocrine, self-stimulatory effects)	Shi et al., 1993
	High	**Exogenous (human trophoblast cells):** ↓ leptin secretion ↑ VEGF secretion; adhesion to uterine epithelial cells; invasion; migration; differentiation	Shi et al., 1993; Islami et al., 2003a; Prast et al., 2008; Lee C. L. et al., 2013; Chen et al., 2015
		**Exogenous (human myometrial strips/smooth muscle cells):** ↓ oxytocin-induced contractions; gap junctions (connexin43)	Ambrus and Rao, 1994; Eta et al., 1994
		**Exogenous (human endometrial epithelial cells):** ↑ VEGF secretion	Berndt et al., 2006
		**Exogenous (human uterine microvascular/umbilical vein endothelial cells):** ↑ proliferation; capillary formation; migration	Zygmunt et al., 2002; Berndt et al., 2006
		**Exogenous (rat aorta explant/chicken chorioallantoic membrane):** ↑ vessel outgrowth and network complexity	Zygmunt et al., 2002; Berndt et al., 2006
		**Exogenous (human uterine natural killer cells):** ↑ proliferation	Kane et al., 2009
		**Exogenous (mouse B cells):** ↑ proliferation of specific cell populations; IL10 production; glycosylated antibody synthesis	Fettke et al., 2016
Relaxin	Low	**Relaxin antibody administration (pregnant mouse uterine arteries):** ↑ vessel wall stiffness	Vodstrcil et al., 2012
	High	**Exogenous (human first trimester extravillous, third trimester cytotrophoblast and HTR-8/SVneo cells):** ↓ apoptosis (↓caspase 3 and cleaved PARP; ↑BCL2) ↑ proliferation; inflammatory markers (IL6 and IL8); invasive potential (↓TIMP1; ↑MMP2 and MMP9)	Maruo et al., 2007; Bryant-Greenwood et al., 2009; Lodhi et al., 2013; Astuti et al., 2015
		**Exogenous (human lower uterine segment fibroblast cells):** ↑ matrix remodeling (↑MMP1 and MMP3; ↓ TIMP1)	Palejwala et al., 2001
		**Exogenous (rat uterine artery):** ↑ relaxation	Longo et al., 2003
		**Exogenous (human endometrial/decidual stromal cells):** ↑ expression of VEGF, IGFBP1, RXFP1	Unemori et al., 1999; Mazella et al., 2004
		**Exogenous (human, pig and rat myometrial strips):** ↓ spontaneous contraction	Maclennan and Grant, 1991; Longo et al., 2003
Leptin	Low	**Leptin antisense oligonucleotide (human third trimester placental explants):** ↑ immunosuppression (HLA-G)	Barrientos et al., 2015
		**Leptin antisense oligonucleotide (human JEG-3 and BeWo cytotrophoblast cells):** ↑apoptosis	Magariños et al., 2007
	High	**Exogenous (human third trimester placental explants):** ↓ apoptosis (caspase 3 activation and p53); triglyceride and cholesterol content ↑ NO production; glycerol release (lipid hydrolysis)	White et al., 2006; Toro et al., 2014
		**Exogenous (human primary first and third trimester trophoblast, JEG-3 and BeWo cells):** ↓ apoptosis (caspase 3 activation and p53); VEGF, estradiol and progesterone release ↑ proliferation; invasion (MMP2, MMP9 and fetal fibronectin); migration; immunosuppression (HLA-G); testosterone production; hCG and IL6 release	Castellucci et al., 2000; Cameo et al., 2003; Islami et al., 2003a; Coya et al., 2006; Magariños et al., 2007; Liu et al., 2009; Toro et al., 2014; Barrientos et al., 2015
		**Exogenous (mouse trophoblast cells):** ↑ invasion; placental lactogen and MMP2	Schulz and Widmaier, 2004; Hughes et al., 2017
		**Exogenous (human myometrial smooth muscle cells):** ↑ proliferation	Barrichon et al., 2015
		**Exogenous (human and bovine endothelial cells):** ↑ proliferation; migration; tube formation; phosphorylation of transcription factor STAT3	Sierra-Honigmann et al., 1998
		**Exogenous (human and rat pancreatic islets):** ↓ insulin production and secretion	Kulkarni et al., 1997; Seufert et al., 1999
		**Exogenous (rat pancreatic** β **cells):** ↑ proliferation	Islam et al., 1997

*CASP, Caspase; CDK, Cyclin dependent kinase; HLA, Human leukocyte antigen; MMP, Matrix metalloproteinase; PARP, Poly (ADP-ribose) polymerase; siRNA, short interfering RNA; TIMP, Tissue inhibitor of metalloproteinase; VEGF, Vascular endothelial growth factor*.

#### Chorionic gonadotropin (CG)

CG, is secreted by the human (hCG) and equine (eCG) placenta, although hCG has been more extensively studied. hCG is a large glycoprotein composed of α and β subunits, of which the α subunit identical to luteinizing hormone (LH), follicle stimulating hormone (FSH) and thyroid stimulating hormone (TSH). As a result, hCG can interact with LH, FSH and TSH receptors. In women, hCG is secreted from the trophoblast from very early in gestation and is thought to be the first placental hormone to act on the mother (Ogueh et al., 2011). Indeed, maternal circulating hCG concentrations peak in the first trimester and then decline toward term (Ogueh et al., 2011). In early pregnancy, hCG maintains corpus luteum allowing the continued secretion of ovarian progesterone and estrogens until the steroidogenic activity of the fetal-placental unit can compensate for maternal ovarian function (Fournier et al., 2015). In particular, hCG increases the abundance of low-density lipoprotein receptor and thus uptake of cholesterol for steroidogenesis. It also enhances the expression and/or activity of steroidogenic enzymes including 3β-hydroxysteroid and aromatase. There is also some evidence which suggests hCG may inhibit factors that promote luteal demise, such as the prostaglandins. The high levels of hCG in early pregnancy are also sufficient to bind to the TSH receptor and may act to increase maternal thyroid hormone production, which as mentioned previously, may exert effects in the mother and fetus.

CG may also play important autocrine and paracrine roles at the maternal-fetal interface. Administration of hCG antisera prevents implantation in marmoset *in vivo* (Hearn et al., 1988). Recent proteomic analysis of estrogen and hCG treated human endometrial epithelial cells demonstrates that hCG targets pathways involved in metabolism, basement membrane and cell connectivity, proliferation and differentiation, cellular adhesion, extracellular-matrix organization, developmental growth, growth factor regulation and cell signaling (Greening et al., 2016). Such pathways are likely to be important for placental development, as attenuating hCG signaling disrupts trophoblast differentiation *in vitro* (Shi et al., 1993). In contrast, supplementing human trophoblast cells with hCG increases their differentiation, migration, invasion and adhesion to uterine epithelial cells, and decreases their leptin secretion *in vitro* (Table 8; hCG; Shi et al., 1993; Prast et al., 2008; Lee C. L. et al., 2013; Chen et al., 2015). hCG also promotes angiogenic vascular endothelial growth factor secretion by both trophoblast and endometrial epithelial cells (Islami et al., 2003a; Berndt et al., 2006) and enhances endothelial tube formation and migration (Zygmunt et al., 2002). Furthermore, hCG is key in suppressing the maternal immune system from mounting a response against paternal antigens carried by the allogenic conceptus. Administration of hCG in a mouse model of spontaneous abortion significantly reduces the number of fetal resorptions due to improved immune tolerance of the fetus (Schumacher et al., 2013). *In vitro*, hCG enhances proliferation of immunosuppressive uterine natural killer cells (Kane et al., 2009), and the production of immunosuppressing IL-10 by B cells (Fettke et al., 2016). hCG can also modulate the immune system even in a non-pregnant state, as shown by its efficacy in preventing the development of autoimmune diabetes in a mouse model (Khil et al., 2007). In pregnancy, hCG additionally inhibits the contractile function of smooth muscle cells in the uterus to help sustain myometrial quiescence (Ambrus and Rao, 1994; Eta et al., 1994), so as to prevent premature expulsion of the fetus. Glycosylation of hCG affects its biological activity and half-life (Fournier et al., 2015). Given its involvement with multiple systems, it is perhaps unsurprising that abnormal concentrations of hCG and hCG glycoforms have been linked with pregnancy complications such as fetal growth restriction and preeclampsia (Chen et al., 2012). However, whether the abnormal concentrations of hCG are cause or consequence of the disorders remains to be determined.

#### Activins

Activins are members of the TGFβ family and were first discovered for their role in stimulating FSH production and determining estrus cyclicity and fertility in mice (Ahn et al., 2004; Sandoval-Guzmán et al., 2012). Activin signaling promotes the decidualization, as well as, apoptosis of endometrial stroma cells (Table 8; Activins; Tessier et al., 2003; Clementi et al., 2013; Yong et al., 2017); processes that accommodate implantation and conceptus development (Peng et al., 2015). Additionally, activin A enhances steroid production, invasion and apoptosis of human trophoblast *in vitro* (Ni et al., 2000; Yu et al., 2012; Li et al., 2015). However, activins may also be of importance in modulating the physiology of the mother during pregnancy (Table 7; Activins). In normal human pregnancy, activin A concentrations gradually rise during gestation and peak at term (Fowler et al., 1998). The placenta is thought to be the main source of activin A in the maternal circulation during pregnancy, given the rapid clearance after delivery of the placenta (Muttukrishna et al., 1997; Fowler et al., 1998). A similar rise of activin in the maternal circulation is observed in pregnant ewes (Jenkin et al., 2001), while the circulating profiles in other species remain undetermined. Nevertheless, in mice, impaired activin signaling leads to poor pregnancy outcomes such as fewer viable pups (Clementi et al., 2013; Peng et al., 2015). However, there is evidence that an increase in activin may also be pathological and detrimental to pregnancy outcome. For instance in pregnant mice, infusion of activin A or plasmid overexpression of activin A results in the development of a preeclamptic phenotype; dams display hypertension and proteinuria, in addition to growth restriction and greater *in utero* deaths (Kim et al., 2008; Lim et al., 2015). The maternal hypertension observed likely results from pathological concentrations of activin A inducing vascular endothelial dysfunction (Yong et al., 2015). In the non-pregnant state, activins are also important for renal glomeruli development (Maeshima et al., 2000), as well as, for bone, fat and muscle metabolism (Yogosawa et al., 2013; Ding et al., 2017; Goh et al., 2017). The possible contributions of activin to these latter functions in pregnancy are currently unclear. Therefore, the impact of activin signaling on these other body systems during pregnancy remains to be determined.

#### Relaxin

Relaxin is a potent vasodilator (Danielson et al., 1999), and regulates hemodynamics in both the non-pregnant and pregnant state (Table 7; Relaxin; Conrad et al., 2004). In pregnant women, circulating relaxin concentration peaks in the first trimester, declines in the second trimester and is maintained until delivery in the third trimester (Quagliarello et al., 1979; Seki et al., 1985). In contrast, circulating relaxin peaks toward term in mice, rats, guinea pigs and hamsters (O'byrne and Steinetz, 1976; O'byrne et al., 1976; Renegar and Owens, 2002). In pregnant mice, relaxin deficiency leads to proteinuria, suggesting a particular role of relaxin in modulating renal function during pregnancy (O'sullivan et al., 2017). In addition, relaxin-deficient mice remain sensitive to vasoconstrictors such as angiotensin and endothelin, and are hypertensive during pregnancy (Marshall et al., 2016a; Mirabito Colafella et al., 2017). During pregnancy, relaxin-deficient mice also display stiffer uterine vessels and fetal growth is retarded (Gooi et al., 2013). Relaxin also enhances capillarisation and glucose uptake of skeletal muscles in non-pregnant mice (Bonner et al., 2013). Taken together, these data highlight the importance of relaxin in mediating changes in maternal vascular function that serve to promote blood flow to the gravid uterus during pregnancy.

Relaxin may play additional roles within the uterus that are important for implantation, placentation and pregnancy maintenance (Tables 7, 8; Relaxin). *In vitro*, relaxin increases decidual cell insulin-like growth factor binding protein-1 expression, a marker of decidualization (Mazella et al., 2004). It also enhances survival and proliferation of cultured human trophoblast cells (Lodhi et al., 2013; Astuti et al., 2015). During early mouse pregnancy, relaxin modulates the uterine expression of genes involved in angiogenesis, steroid hormone action and remodeling (Marshall et al., 2016b). Indeed in pregnant marmosets, exogenous relaxin improves uterine and placental growth (Einspanier et al., 2009). Relaxin infusion also alters the endometrial lymphocyte number *in vivo* (Goldsmith et al., 2004), which suggests a possible role of relaxin in achieving immune tolerance of the allogenic conceptus. Relaxin impedes spontaneous contractility of myometrium in humans, rats and pigs (Maclennan and Grant, 1991; Longo et al., 2003), and is thus thought to play a role in regulating the onset of parturition (Vannuccini et al., 2016). In mice with a deficiency in relaxin signaling, obstructed deliveries occur at a higher rate due to poor maturation of the cervix (Zhao et al., 1999; Kamat et al., 2004; Krajnc-Franken et al., 2004; Kaftanovskaya et al., 2015). Conversely in hamsters, the rise in circulating relaxin toward term coincides with cervical ripening in preparation for delivery (O'byrne et al., 1976). Insufficient relaxin signaling also impedes mammary development through excessive duct dilation and reduces the nursing of offspring in mice (Zhao et al., 1999; Kamat et al., 2004; Krajnc-Franken et al., 2004). Conversely, overexpression leads to hypertrophy of the nipples in non-pregnant mice (Feng et al., 2006). Hence, relaxin is important in driving changes at the maternal-fetal interface that establish pregnancy, adapts the cardiovascular system of the mother to support the pregnancy and prepares the mother for lactation post-partum.

#### Parathyroid hormone-related protein (PTHrP)

During pregnancy, the placenta serves as an additional source of PTHrP (Bowden et al., 1994; Emly et al., 1994), a key hormone involved in bone metabolism (Table 7; PTHrP). PTHrP concentrations in the maternal blood rise throughout gestation in humans (Gallacher et al., 1994; Ardawi et al., 1997; Hirota et al., 1997) and correlate with the rise in maternal circulating calcium during pregnancy (Bertelloni et al., 1994). However, excessively high circulating PTHrP can lead to hypercalcaemia during pregnancy (Winter and Appelman-Dijkstra, 2017). PTHrP increases maternal bone resorption, thereby enabling calcium transfer from mother to fetus for bone development (Salles, 2016). Thus, it is perhaps not surprising that complete knockout of PTHrP in mice is lethal at birth in association with abnormal bone development (Karaplis et al., 1994). Carrying one defective PTHrP copy is enough to also impede bone development and reduce snout length in mice (Amizuka et al., 1996). Mammary-specific PTHrP deletion increases maternal bone mass and protects against lactation-associated bone loss by reducing bone turnover in mice (Williams et al., 1998; Vanhouten et al., 2003). However, deleting bone-specific PTHrP increases skeletal fragility, both in the non-pregnant and pregnant state (Kirby et al., 2011). PTHrP infusion of lactating goats increases mammary gland uptake calcium, phosphorous and magnesium for transfer in milk to the neonate (Barlet et al., 1992). These findings imply that a fine balance of PTHrP production by gestational and maternal tissues must be achieved for appropriate regulation of maternal bone metabolism and offspring calcium requirements during pregnancy and lactation.

Placental-derived PTHrP may also exert additional effects on the placenta and the mother which are beneficial for offspring development and growth. PTHrP stimulates the proliferation, differentiation, outgrowth and calcium uptake of trophoblast *in vitro* (Table 8; PTHrP; Hershberger and Tuan, 1998; El-Hashash and Kimber, 2006). *In vivo*, blocking PTHrP signaling during mouse pregnancy leads to excessive uterine growth and decidualization in association with a decrease in decidual cell apoptosis (Williams et al., 1998; Vanhouten et al., 2003). Moreover, over-expression of PTHrP impairs mammary gland branching morphogenesis (Wysolmerski et al., 1995; Dunbar et al., 2001). These studies highlight a possible important regulatory role of PTHrP in the control of decidualization and mammary gland development *in vivo*. In non-pregnant mice, PTHrP enhances pancreatic β-cells proliferation and insulin secretion whilst it inhibits islet cell apoptosis (Vasavada et al., 1996; Porter et al., 1998; Cebrian et al., 2002; Fujinaka et al., 2004). It also increases renal plasma flow and glomerular filtration rate, and exerts proliferative effects on renal glomerular and tubule cells in rodents (Izquierdo et al., 2006; Romero et al., 2010). Additionally, *in vitro* studies show PTHrP can induce relaxation of uterine arteries (Meziani et al., 2005). However, the significance of PTHrP on glucose-insulin dynamics and renal and vascular function of the mother during pregnancy remains to be investigated.

#### Leptin

Leptin is an abundant circulating hormone involved in regulating appetite. In the non-pregnant state, the adipose tissue is the exclusive source of circulating leptin. During pregnancy in humans, baboons and mice, concentrations of leptin rapidly rise throughout gestation, peaking toward term (Highman et al., 1998; Henson et al., 1999; Malik et al., 2005). The rise in leptin positively correlates with increases in maternal body fat (Highman et al., 1998). In humans, blood leptin rapidly falls to non-pregnant concentrations within 24 h of delivery, indicating that the placenta contributes to the main rise of leptin in pregnancy (Masuzaki et al., 1997). In particular, leptin is produced by the human placental trophoblast cells (Masuzaki et al., 1997). A similar post-pregnancy decline and placental trophoblast expression is seen in baboons (Henson et al., 1999). However, this is not the case for mice, as the murine placenta does not produce leptin (Malik et al., 2005). Nevertheless, leptin studies in mice still provide useful knowledge about pregnancy-related effects of leptin (Table 7; Leptin). For instance, leptin in pregnancy helps prepare the mother for lactation, as a deficiency results in impaired mammary gland development, which is detrimental for lactation post-delivery (Mounzih et al., 1998; Malik et al., 2001). Another significant effect of leptin in pregnancy observed through mouse studies is leptin resistance, whereby the dam increases her food intake in mid-pregnancy to meet increased energy demands despite an increase in circulating leptin, which in the non-pregnant state would lead to satiety (Mounzih et al., 1998). In contrast, excessive leptin significantly decreases maternal food intake and restricts feto-placental growth (Yamashita et al., 2001). Leptin exposure of rat and human islets and cultured insulinoma cells significantly decreases insulin production *in vitro*, demonstrating that leptin may be directly involved in glucose metabolism (Table 8; Leptin; Kulkarni et al., 1997). Indeed dysfunctional leptin signaling in pregnancy leads to the spontaneous development of a gestational diabetic phenotype in db/+ mice, who are heterozygous for the leptin receptor (Table 7; Leptin; Yamashita et al., 2001). Further *in vitro* studies on placental explants or trophoblast cultures highlight a potential for leptin to be involved in immune modulation and placental hormone production, given its stimulatory effects on HLA-G and hCG expression (Table 8; Leptin; Chardonnens et al., 1999; Islami et al., 2003a,b; Barrientos et al., 2015). Additional effects of leptin on the placenta are thoroughly reviewed elsewhere (Schanton et al., 2018). Therefore, placental leptin can have systemic effects on the mother in pregnancy.

## Conclusion

**Figure 2:**
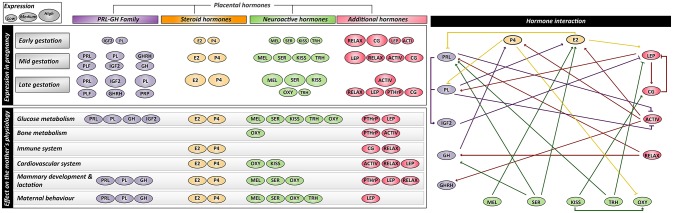
Summary of expression profiles, interactions and maternal physiological effects of placental-derived hormones. PRL, prolactin; PL, placental lactogen; PLF, proliferins; PRP, proliferin-related proteins; GH, growth hormone; GHRH, growth hormone releasing hormone; IGF1/2, insulin-like growth factor-1/2; E2, estrogen; P4, progesterone; MEL, melatonin; SER, serotonin; KISS, kisspeptin; OXY, Oxytocin; TRH, thyrotropin-releasing hormone; RELAX, relaxin; ACTIV, activin; CG, chorionic gonadotropin; LEP, leptin; PTHrP, parathyroid hormone-related protein.

Pregnancy represents a unique physiological paradigm; there are dynamic and reversible changes in the function of many organ systems in the mother that are designed to support offspring development. In part, these changes are signaled via the placental secretion of hormones, which in turn, alter in abundance, interact with one another and exert wide effects on maternal tissues during pregnancy. For instance, steroid hormones modulate most systems of the mother throughout pregnancy. However, they also alter the production of other hormones, such as prolactin and placental lactogens, which in turn, may contribute to the physiological changes in the mother (Figure 2). However, further work is required to better define how placental hormones elicit their actions in the mother, as well as, identify the extent to which they interplay with hormones produced by maternal tissues. As the endocrine and metabolic state of the mother is also influenced by her environment, maternal conditions such as poor nutrition and obesity may modulate placental hormone production and pregnancy adaptations. Indeed, previous work has shown that an obesogenic diet during pregnancy alters the expression of PRL/PL genes in the placenta in association with mal-adaptations of maternal metabolism in mice (Musial et al., 2017). Further studies are nonetheless needed to assess the interaction of the maternal environment with placental endocrine function. Placental hormones are also released into the fetal circulation, where they may have direct impacts on fetal growth and development (Freemark, 2010). Investigations exploring the importance of placental endocrine function on fetal growth, independent of the mother, will require future examination. Collectively, further studies on the nature and role of placental endocrine function in maternal adaptations and fetal growth will undoubtedly provide novel insights into understanding of the potential causes of obstetrical syndromes such as gestational diabetes and preeclampsia that are marked by maternal physiological maladaptation.

## Author contributions

TN and HY substantially contributed to the conception of the work, drafting and revision of the manuscript, preparation of the tables and approved of the final version. JL-T substantially contributed to the conception of the work, drafting and revision of the manuscript, preparation of the figures and approved of the final version. AS-P substantially contributed to the conception of the work, critical revision of the manuscript for intellectual content and approved of the final version.

### Conflict of interest statement

The authors declare that the research was conducted in the absence of any commercial or financial relationships that could be construed as a potential conflict of interest.

## References

[B1] Abd-Allah A. R. El-Sayed El S. M. Abdel-Wahab M. H. Hamada F. M. (2003). Effect of melatonin on estrogen and progesterone receptors in relation to uterine contraction in rats. Pharmacol. Res. 47, 349–354. 10.1016/S1043-6618(03)00014-8 12644393

[B2] Abribat T. Lapierre H. Dubreuil P. Pelletier G. Gaudreau P. Brazeau P.. (1990). Insulin-like growth factor-I concentration in Holstein female cattle: variations with age, stage of lactation and growth hormone-releasing factor administration. Domest. Anim. Endocrinol. 7, 93–102. 10.1016/0739-7240(90)90058-8 2107053

[B3] Açikgöz S. Bayar U. O. Can M. Güven B. Mungan G. Dogan S.. (2013). Levels of oxidized LDL, estrogens, and progesterone in placenta tissues and serum paraoxonase activity in preeclampsia. Mediators Inflamm. 2013:862982. 10.1155/2013/862982 23606795 PMC3625559

[B4] Ackermann A. M. Gannon M. (2007). Molecular regulation of pancreatic beta-cell mass development, maintenance, and expansion. J. Mol. Endocrinol. 38, 193–206. 10.1677/JME-06-0053 17293440

[B5] Adamah-Biassi E. B. Hudson R. L. Dubocovich M. L. (2014). Genetic deletion of MT1 melatonin receptors alters spontaneous behavioral rhythms in male and female C57BL/6 mice. Horm. Behav. 66, 619–627. 10.1016/j.yhbeh.2014.08.012 25200199 PMC4698802

[B6] Adamova Z. Ozkan S. Khalil R. A. (2009). Vascular and cellular calcium in normal and hypertensive pregnancy. Curr. Clin. Pharmacol. 4, 172–190. 10.2174/157488409789375320 19500073 PMC2852626

[B7] Ahmed-Sorour H. Bailey C. J. (1980). Role of ovarian hormones in the long-term control of glucose homeostasis. Interaction with insulin, glucagon and epinephrine. Horm. Res. 13, 396–403. 10.1159/000179307 7024081

[B8] Ahmed-Sorour H. Bailey C. J. (1981). Role of ovarian hormones in the long-term control of glucose homeostasis, glycogen formation and gluconeogenesis. Ann. Nutr. Metab. 25, 208–212. 10.1159/000176496 7305285

[B9] Ahn J. M. Jung H. K. Cho C. Choi D. Mayo K. E. Cho B. N. (2004). Changes in the reproductive functions of mice due to injection of a plasmid expressing an inhibin alpha-subunit into muscle: a transient transgenic model. Mol. Cells 18, 79–86. 15359127

[B10] Ahumada-Solórzano S. M. Martínez-Moreno C. G. Carranza M. Ávila-Mendoza J. Luna-Acosta J. L. Harvey S.. (2016). Autocrine/paracrine proliferative effect of ovarian GH and IGF-I in chicken granulosa cell cultures. Gen. Comp. Endocrinol. 234, 47–56. 10.1016/j.ygcen.2016.05.008 27174747

[B11] Aizawa-Abe M. Ogawa Y. Masuzaki H. Ebihara K. Satoh N. Iwai H.. (2000). Pathophysiological role of leptin in obesity-related hypertension. J. Clin. Invest. 105, 1243–1252. 10.1172/JCI8341 10791999 PMC315441

[B12] Alenina N. Kikic D. Todiras M. Mosienko V. Qadri F. Plehm R.. (2009). Growth retardation and altered autonomic control in mice lacking brain serotonin. Proc. Natl. Acad. Sci. U.S.A. 106, 10332–10337. 10.1073/pnas.0810793106 19520831 PMC2700938

[B13] Alperin M. Kaddis T. Pichika R. Esparza M. C. Lieber R. L. (2016). Pregnancy-induced adaptations in intramuscular extracellular matrix of rat pelvic floor muscles. Am. J. Obstet. Gynecol. 215, 210 e211–210 e217. 10.1016/j.ajog.2016.02.018 26875952 PMC5450638

[B14] Alperin M. Lawley D. M. Esparza M. C. Lieber R. L. (2015). Pregnancy-induced adaptations in the intrinsic structure of rat pelvic floor muscles. Am. J. Obstet. Gynecol. 213, 191 e191–191 e197. 10.1016/j.ajog.2015.05.012 25979618 PMC4757427

[B15] Ambrus G. Rao C. V. (1994). Novel regulation of pregnant human myometrial smooth muscle cell gap junctions by human chorionic gonadotropin. Endocrinology 135, 2772–2779. 10.1210/endo.135.6.7988470 7988470

[B16] Amico J. A. Vollmer R. R. Cai H. M. Miedlar J. A. Rinaman L. (2005). Enhanced initial and sustained intake of sucrose solution in mice with an oxytocin gene deletion. Am. J. Physiol. Regul. Integr. Comp. Physiol. 289, R1798–R1806. 10.1152/ajpregu.00558.2005 16150836

[B17] Amizuka N. Karaplis A. C. Henderson J. E. Warshawsky H. Lipman M. L. Matsuki Y.. (1996). Haploinsufficiency of parathyroid hormone-related peptide (PTHrP) results in abnormal postnatal bone development. Dev. Biol. 175, 166–176. 10.1006/dbio.1996.0104 8608863

[B18] Angoa-Pérez M. Kane M. J. Sykes C. E. Perrine S. A. Church M. W. Kuhn D. M. (2014). Brain serotonin determines maternal behavior and offspring survival. Genes Brain Behav. 13, 579–591. 10.1111/gbb.12159 25077934 PMC4804711

[B19] Angoa-Pérez M. Kuhn D. M. (2015). Neuronal serotonin in the regulation of maternal behavior in rodents. Neurotransmitter (Houst) 2:e615. 10.14800/nt.615 27148594 PMC4852377

[B20] Antonijevic I. A. Leng G. Luckman S. M. Douglas A. J. Bicknell R. J. Russell J. A. (1995). Induction of uterine activity with oxytocin in late pregnant rats replicates the expression of c-fos in neuroendocrine and brain stem neurons as seen during parturition. Endocrinology 136, 154–163. 10.1210/endo.136.1.7828526 7828526

[B21] Apa R. Lanzone A. Miceli F. Mastrandrea M. Macchione E. Caruso A.. (1995). Growth hormone-releasing factor stimulates meiotic maturation in follicle- and cumulus-enclosed rat oocyte. Mol. Cell. Endocrinol. 112, 195–201. 10.1016/0303-7207(95)03599-3 7489823

[B22] Ardawi M. S. Nasrat H. A. BA'Aqueel H. S. (1997). Calcium-regulating hormones and parathyroid hormone-related peptide in normal human pregnancy and postpartum: a longitudinal study. Eur. J. Endocrinol. 137, 402–409. 10.1530/eje.0.1370402 9368509

[B23] Arletti R. Benelli A. Bertolini A. (1989). Influence of oxytocin on feeding behavior in the rat. Peptides 10, 89–93. 10.1016/0196-9781(89)90082-X 2748428

[B24] Arletti R. Benelli A. Bertolini A. (1990). Oxytocin inhibits food and fluid intake in rats. Physiol. Behav. 48, 825–830. 10.1016/0031-9384(90)90234-U 2087513

[B25] Arrowsmith S. Wray S. (2014). Oxytocin: its mechanism of action and receptor signalling in the myometrium. J. Neuroendocrinol. 26, 356–369. 10.1111/jne.12154 24888645

[B26] Arumugam R. Fleenor D. Freemark M. (2014). Knockdown of prolactin receptors in a pancreatic beta cell line: effects on DNA synthesis, apoptosis, and gene expression. Endocrine 46, 568–576. 10.1007/s12020-013-0073-1 24114406 PMC3984618

[B27] Askew R. D. Ramsden D. B. (1984). Effect of repeated stimulation by thyrotropin-releasing hormone (TRH) on thyrotropin and prolactin secretion in perfused euthyroid and hypothyroid rat pituitary fragments. Horm. Res. 20, 269–276. 10.1159/000180007 6439618

[B28] Astuti Y. Nakabayashi K. Deguchi M. Ebina Y. Yamada H. (2015). Human recombinant H2 relaxin induces AKT and GSK3beta phosphorylation and HTR-8/SVneo cell proliferation. Kobe J. Med. Sci. 61, E1–8. 10.24546/81008925 25868609

[B29] Atherton J. C. Dark J. M. Garland H. O. Morgan M. R. Pidgeon J. Soni S. (1982). Changes in water and electrolyte balance, plasma volume and composition during pregnancy in the rat. J. Physiol. (Lond). 330, 81–93. 10.1113/jphysiol.1982.sp014330 7175756 PMC1225287

[B30] Ayar A. Kutlu S. Yilmaz B. Kelestimur H. (2001). Melatonin inhibits spontaneous and oxytocin-induced contractions of rat myometrium *in vitro*. Neuro Endocrinol. Lett. 22, 199–207. 11449192

[B31] Azukizawa M. Murata Y. Ikenoue T. Martin C. B. Jr. Hershman J. M. (1976). Effect of thyrotropin-releasing hormone on secretion of thyrotropin, prolactin, thyroxine, and triiodothyronine in pregnant and fetal rhesus monkeys. J. Clin. Endocrinol. Metab. 43, 1020–1028. 10.1210/jcem-43-5-1020 825527

[B32] Bacq Y. (2013). The liver in normal pregnancy, in Madame Curie Bioscience Database. (Austin, TX: Landes Bioscience).

[B33] Bader R. A. Bader M. E. Rose D. F. Braunwald E. (1955). Hemodynamics at rest and during exercise in normal pregnancy as studies by cardiac catheterization. J. Clin. Invest. 34, 1524–1536. 10.1172/JCI103205 13263433 PMC438730

[B34] Bae M. H. Lee M. J. Bae S. K. Lee O. H. Lee Y. M. Park B. C.. (1998). Insulin-like growth factor II (IGF-II) secreted from HepG2 human hepatocellular carcinoma cells shows angiogenic activity. Cancer Lett. 128, 41–46. 10.1016/S0304-3835(98)00044-5 9652791

[B35] Baeyens L. Hindi S. Sorenson R. L. German M. S. (2016). beta-Cell adaptation in pregnancy. Diabetes Obes. Metab. 18(Suppl. 1), 63–70. 10.1111/dom.12716 27615133 PMC5384851

[B36] Bähr I. Mühlbauer E. Schucht H. Peschke E. (2011). Melatonin stimulates glucagon secretion *in vitro* and *in vivo*. J. Pineal Res. 50, 336–344. 10.1111/j.1600-079X.2010.00848.x 21244480

[B37] Bailey C. J. Ahmed-Sorour H. (1980). Role of ovarian hormones in the long-term control of glucose homeostasis. Effects of insulin secretion. Diabetologia 19, 475–481. 10.1007/BF00281829 7004967

[B38] Bajoria R. Babawale M. (1998). Ontogeny of endogenous secretion of immunoreactive-thyrotropin releasing hormone by the human placenta. J. Clin. Endocrinol. Metab. 83, 4148–4155. 10.1210/jcem.83.11.5216 9814505

[B39] Banerjee R. R. Cyphert H. A. Walker E. M. Chakravarthy H. Peiris H. Gu X.. (2016). Gestational diabetes mellitus from inactivation of prolactin receptor and mafb in islet beta-cells. Diabetes 65, 2331–2341. 10.2337/db15-1527 27217483 PMC4955982

[B40] Bani G. Maurizi M. Bigazzi M. Bani Sacchi T. (1995). Effects of relaxin on the endometrial stroma. Studies in mice. Biol. Reprod 53, 253–262. 10.1095/biolreprod53.2.253 7492676

[B41] Barbour L. A. Shao J. Qiao L. Leitner W. Anderson M. Friedman J. E.. (2004). Human placental growth hormone increases expression of the p85 regulatory unit of phosphatidylinositol 3-kinase and triggers severe insulin resistance in skeletal muscle. Endocrinology 145, 1144–1150. 10.1210/en.2003-1297 14633976

[B42] Barbour L. A. Shao J. Qiao L. Pulawa L. K. Jensen D. R. Bartke A.. (2002). Human placental growth hormone causes severe insulin resistance in transgenic mice. Am. J. Obstet. Gynecol. 186, 512–517. 10.1067/mob.2002.121256 11904616

[B43] Barker D. J. (2004). The developmental origins of well-being. Philos. Trans. R. Soc. Lond. B Biol. Sci. 359, 1359–1366. 10.1098/rstb.2004.1518 15347527 PMC1693427

[B44] Barlet J. P. Champredon C. Coxam V. Davicco M. J. Tressol J. C. (1992). Parathyroid hormone-related peptide might stimulate calcium secretion into the milk of goats. J. Endocrinol. 132, 353–359. 10.1677/joe.0.1320353 1564419

[B45] Barrichon M. Hadi T. Wendremaire M. Ptasinski C. Seigneuric R. Marcion G.. (2015). Dose-dependent biphasic leptin-induced proliferation is caused by non-specific IL-6/NF-kappaB pathway activation in human myometrial cells. Br. J. Pharmacol. 172, 2974–2990. 10.1111/bph.13100 25653112 PMC4459017

[B46] Barrientos G. Toro A. Moschansky P. Cohen M. Garcia M. G. Rose M.. (2015). Leptin promotes HLA-G expression on placental trophoblasts via the MEK/Erk and PI3K signaling pathways. Placenta 36, 419–426. 10.1016/j.placenta.2015.01.006 25649687

[B47] Basraon S. Costantine M. M. (2011). Mood disorders in pregnant women with thyroid dysfunction. Clin. Obstet. Gynecol. 54, 506–514. 10.1097/GRF.0b013e3182273089 21857182

[B48] Bearfield C. Jauniaux E. Groome N. Sargent I. L. Muttukrishna S. (2005). The secretion and effect of inhibin A, activin A and follistatin on first-trimester trophoblasts *in vitro*. Eur. J. Endocrinol. 152, 909–916. 10.1530/eje.1.01928 15941932

[B49] Ben-Jonathan N. Hugo E. (2015). Prolactin (PRL) in adipose tissue: regulation and functions. Adv. Exp. Med. Biol. 846, 1–35. 10.1007/978-3-319-12114-7_1 25472532

[B50] Berkane N. Liere P. Oudinet J. P. Hertig A. Lefèvre G. Pluchino N.. (2017). From pregnancy to preeclampsia: a key role for estrogens. Endocr. Rev. 38, 123–144. 10.1210/er.2016-1065 28323944

[B51] Berndt S. Perrier D'hauterive S. Blacher S. Péqueux C. Lorquet S. Munaut C.. (2006). Angiogenic activity of human chorionic gonadotropin through LH receptor activation on endothelial and epithelial cells of the endometrium. FASEB J. 20, 2630–2632. 10.1096/fj.06-5885fje 17065221

[B52] Bertelloni S. Baroncelli G. I. Pelletti A. Battini R. Saggese G. (1994). Parathyroid hormone-related protein in healthy pregnant women. Calcif. Tissue Int. 54, 195–197. 10.1007/BF00301677 8055365

[B53] Bethea C. L. Cronin M. J. Haluska G. J. Novy M. J. (1989). The effect of relaxin infusion on prolactin and growth hormone secretion in monkeys. J. Clin. Endocrinol. Metab. 69, 956–962. 10.1210/jcem-69-5-956 2793997

[B54] Billestrup N. Nielsen J. H. (1991). The stimulatory effect of growth hormone, prolactin, and placental lactogen on beta-cell proliferation is not mediated by insulin-like growth factor-I. Endocrinology 129, 883–888. 10.1210/endo-129-2-883 1677331

[B55] Binart N. Helloco C. Ormandy C. J. Barra J. Clément-Lacroix P. Baran N.. (2000). Rescue of preimplantatory egg development and embryo implantation in prolactin receptor-deficient mice after progesterone administration. Endocrinology 141, 2691–2697. 10.1210/endo.141.7.7568 10875275

[B56] Binko J. Majewski H. (1998). 17β-Estradiol reduces vasoconstriction in endothelium-denuded rat aortas through inducible NOS. Am. J. Physiol. Heart Circ. Physiol. 274, H853–H859. 10.1152/ajpheart.1998.274.3.H853 9530196

[B57] Bittorf T. Jaster R. Soares M. J. Seiler J. Brock J. Friese K.. (2000). Induction of erythroid proliferation and differentiation by a trophoblast-specific cytokine involves activation of the JAK/STAT pathway. J. Mol. Endocrinol. 25, 253–262. 10.1677/jme.0.0250253 11013351

[B58] Bjøro K. Stray-Pedersen S. (1986). Effects of vasoactive autacoids on different segments of human umbilicoplacental vessels. Gynecol. Obstet. Invest. 22, 1–6. 10.1159/000298881 2875016

[B59] Blanchard M. M. Goodyer C. G. Charrier J. Kann G. Garcia-Villar R. Bousquet-Melou A.. (1991). GRF treatment of late pregnant ewes alters maternal and fetal somatotropic axis activity. Am. J. Physiol. 260, E575–580. 10.1152/ajpendo.1991.260.4.E575 1673320

[B60] Bonner J. S. Lantier L. Hocking K. M. Kang L. Owolabi M. James F. D. (2013). Relaxin treatment reverses insulin resistance in mice fed a high-fat diet. Diabetes 62, 3251–3260. 10.2337/db13-0033 23801576 PMC3749347

[B61] Boparai R. K. Arum O. Khardori R. Bartke A. (2010). Glucose homeostasis and insulin sensitivity in growth hormone-transgenic mice: a cross-sectional analysis. Biol. Chem. 391, 1149–1155. 10.1515/bc.2010.124 20707609 PMC4009680

[B62] Bosch O. J. Neumann I. D. (2012). Both oxytocin and vasopressin are mediators of maternal care and aggression in rodents: from central release to sites of action. Horm. Behav. 61, 293–303. 10.1016/j.yhbeh.2011.11.002 22100184

[B63] Bowden S. J. Emly J. F. Hughes S. V. Powell G. Ahmed A. Whittle M. J.. (1994). Parathyroid hormone-related protein in human term placenta and membranes. J. Endocrinol. 142, 217–224. 10.1677/joe.0.1420217 7930994

[B64] Bowe J. E. Foot V. L. Amiel S. A. Huang G. C. Lamb M. W. Lakey J.. (2012). GPR54 peptide agonists stimulate insulin secretion from murine, porcine and human islets. Islets 4, 20–23. 10.4161/isl.18261 22192948

[B65] Bowe J. E. King A. J. Kinsey-Jones J. S. Foot V. L. Li X. F. O'byrne K. T.. (2009). Kisspeptin stimulation of insulin secretion: mechanisms of action in mouse islets and rats. Diabetologia 52, 855–862. 10.1007/s00125-009-1283-1 19221709

[B66] Branisteanu D. D. Mathieu C. (2003). Progesterone in gestational diabetes mellitus: guilty or not guilty? Trends Endocrinol. Metab. 14, 54–56. 10.1016/S1043-2760(03)00003-1 12591170

[B67] Brelje T. C. Allaire P. Hegre O. Sorenson R. L. (1989). Effect of prolactin versus growth hormone on islet function and the importance of using homologous mammosomatotropic hormones. Endocrinology 125, 2392–2399. 10.1210/endo-125-5-2392 2676483

[B68] Brelje T. C. Scharp D. W. Lacy P. E. Ogren L. Talamantes F. Robertson M.. (1993). Effect of homologous placental lactogens, prolactins, and growth hormones on islet B-cell division and insulin secretion in rat, mouse, and human islets: implication for placental lactogen regulation of islet function during pregnancy. Endocrinology 132, 879–887. 10.1210/endo.132.2.8425500 8425500

[B69] Bridges R. S. (2015). Neuroendocrine regulation of maternal behavior. Front. Neuroendocrinol. 36, 178–196. 10.1016/j.yfrne.2014.11.007 25500107 PMC4342279

[B70] Bridges R. S. Millard W. J. (1988). Growth hormone is secreted by ectopic pituitary grafts and stimulates maternal behavior in rats. Horm. Behav. 22, 194–206. 10.1016/0018-506X(88)90066-9 3397052

[B71] Bridges R. S. Robertson M. C. Shiu R. P. Sturgis J. D. Henriquez B. M. Mann P. E. (1997). Central lactogenic regulation of maternal behavior in rats: steroid dependence, hormone specificity, and behavioral potencies of rat prolactin and rat placental lactogen I. Endocrinology 138, 756–763. 10.1210/endo.138.2.4921 9003012

[B72] Brockus K. E. Hart C. G. Gilfeather C. L. Fleming B. O. Lemley C. O. (2016). Dietary melatonin alters uterine artery hemodynamics in pregnant Holstein heifers. Domest. Anim. Endocrinol. 55, 1–10. 10.1016/j.domaniend.2015.10.006 26641925

[B73] Brown A. G. Leite R. S. Strauss J. F. III. (2004). Mechanisms underlying “functional” progesterone withdrawal at parturition. Ann. N. Y. Acad. Sci. 1034, 36–49. 10.1196/annals.1335.004 15731298

[B74] Brown P. A. Davis W. C. Draghia-Akli R. (2004). Immune-enhancing effects of growth hormone-releasing hormone delivered by plasmid injection and electroporation. Mol. Ther. 10, 644–651. 10.1016/j.ymthe.2004.06.1015 15451448

[B75] Brown P. A. Khan A. S. Draghia-Akli R. Pope M. A. Bodles-Brakhop A. M. Kern D. R. (2012). Effects of administration of two growth hormone-releasing hormone plasmids to gilts on sow and litter performance for the subsequent three gestations. Am. J. Vet. Res. 73, 1428–1434. 10.2460/ajvr.73.9.1428 22924725

[B76] Bryant-Greenwood G. D. Yamamoto S. Y. Sadowsky D. W. Gravett M. G. Novy M. J. (2009). Relaxin stimulates interleukin-6 and interleukin-8 secretion from the extraplacental chorionic cytotrophoblast. Placenta 30, 599–606. 10.1016/j.placenta.2009.04.009 19467703

[B77] Bryzgalova G. Gao H. Ahren B. Zierath J. R. Galuska D. Steiler T. L.. (2006). Evidence that oestrogen receptor-alpha plays an important role in the regulation of glucose homeostasis in mice: insulin sensitivity in the liver. Diabetologia 49, 588–597. 10.1007/s00125-005-0105-3 16463047

[B78] Bustamante J. J. Copple B. L. Soares M. J. Dai G. (2010). Gene profiling of maternal hepatic adaptations to pregnancy. Liver Int. 30, 406–415. 10.1111/j.1478-3231.2009.02183.x 20040050 PMC4356012

[B79] Bustamante J. J. Dai G. Soares M. J. (2008). Pregnancy and lactation modulate maternal splenic growth and development of the erythroid lineage in the rat and mouse. Reprod. Fertil. Dev. 20, 303–310. 10.1071/RD07106 18255020

[B80] Cameo P. Bischof P. Calvo J. C. (2003). Effect of leptin on progesterone, human chorionic gonadotropin, and interleukin-6 secretion by human term trophoblast cells in culture. Biol. Reprod. 68, 472–477. 10.1095/biolreprod.102.006122 12533410

[B81] Camerino C. (2009). Low sympathetic tone and obese phenotype in oxytocin-deficient mice. Obesity (Silver. Spring). 17, 980–984. 10.1038/oby.2009.12 19247273

[B82] Carter A. M. (2012). Evolution of placental function in mammals: the molecular basis of gas and nutrient transfer, hormone secretion, and immune responses. Physiol. Rev. 92, 1543–1576. 10.1152/physrev.00040.2011 23073626

[B83] Casellas A. Mallol C. Salavert A. Jimenez V. Garcia M. Agudo J.. (2015). Insulin-like growth factor 2 overexpression induces beta-cell dysfunction and increases beta-cell susceptibility to damage. J. Biol. Chem. 290, 16772–16785. 10.1074/jbc.M115.642041 25971976 PMC4505425

[B84] Castellucci M. De Matteis R. Meisser A. Cancello R. Monsurrò V. Islami D.. (2000). Leptin modulates extracellular matrix molecules and metalloproteinases: possible implications for trophoblast invasion. Mol. Hum. Reprod. 6, 951–958. 10.1093/molehr/6.10.951 11006325

[B85] Castillo-Melendez M. Yawno T. Sutherland A. Jenkin G. Wallace E. M. Miller S. L. (2017). effects of antenatal melatonin treatment on the cerebral vasculature in an ovine model of fetal growth restriction. Dev. Neurosci. 39, 323–337. 10.1159/000471797 28467985

[B86] Catalano P. M. Hoegh M. Minium J. Huston-Presley L. Bernard S. Kalhan S.. (2006). Adiponectin in human pregnancy: implications for regulation of glucose and lipid metabolism. Diabetologia 49, 1677–1685. 10.1007/s00125-006-0264-x 16752186

[B87] Cattaneo M. G. Chini B. Vicentini L. M. (2008). Oxytocin stimulates migration and invasion in human endothelial cells. Br. J. Pharmacol. 153, 728–736. 10.1038/sj.bjp.0707609 18059319 PMC2259201

[B88] Cebrian A. García-Ocaña A. Takane K. K. Sipula D. Stewart A. F. Vasavada R. C. (2002). Overexpression of parathyroid hormone-related protein inhibits pancreatic beta-cell death *in vivo* and *in vitro*. Diabetes 51, 3003–3013. 10.2337/diabetes.51.10.3003 12351440

[B89] Cetković A. Miljic D. Ljubić A. Patterson M. Ghatei M. Stamenkovic J.. (2012). Plasma kisspeptin levels in pregnancies with diabetes and hypertensive disease as a potential marker of placental dysfunction and adverse perinatal outcome. Endocr. Res. 37, 78–88. 10.3109/07435800.2011.639319 22489921

[B90] Chandran S. Cairns M. T. O'brien M. Smith T. J. (2014). Transcriptomic effects of estradiol treatment on cultured human uterine smooth muscle cells. Mol. Cell. Endocrinol. 393, 16–23. 10.1016/j.mce.2014.05.020 24942541

[B91] Chang J. Streitman D. (2012). Physiologic adaptations to pregnancy. Neurol. Clin. 30, 781–789. 10.1016/j.ncl.2012.05.001 22840789

[B92] Chapman A. B. Abraham W. T. Zamudio S. Coffin C. Merouani A. Young D.. (1998). Temporal relationships between hormonal and hemodynamic changes in early human pregnancy. Kidney Int. 54, 2056–2063. 10.1046/j.1523-1755.1998.00217.x 9853271

[B93] Chardonnens D. Cameo P. Aubert M. L. Pralong F. P. Islami D. Campana A.. (1999). Modulation of human cytotrophoblastic leptin secretion by interleukin-1α and 17β-oestradiol and its effect on HCG secretion. Mol. Hum. Reprod. 5, 1077–1082. 10.1093/molehr/5.11.1077 10541571

[B94] Chataigneau T. Zerr M. Chataigneau M. Hudlett F. Hirn C. Pernot F. (2004). Chronic treatment with progesterone but not medroxyprogesterone acetate restores the endothelial control of vascular tone in the mesenteric artery of ovariectomized rats. Menopause 11, 255–263. 10.1097/01.GME.0000097847.95550.E3 15167304

[B95] Chaves V. E. Tilelli C. Q. Brito N. A. Brito M. N. (2013). Role of oxytocin in energy metabolism. Peptides 45, 9–14. 10.1016/j.peptides.2013.04.010 23628372

[B96] Chehab F. F. Lim M. E. Lu R. (1996). Correction of the sterility defect in homozygous obese female mice by treatment with the human recombinant leptin. Nat. Genet. 12, 318–320. 10.1038/ng0396-318 8589726

[B97] Chehab F. F. Mounzih K. Lu R. Lim M. E. (1997). Early onset of reproductive function in normal female mice treated with leptin. Science 275, 88–90. 10.1126/science.275.5296.88 8974400

[B98] Chen J. Z. Sheehan P. M. Brennecke S. P. Keogh R. J. (2012). Vessel remodelling, pregnancy hormones and extravillous trophoblast function. Mol. Cell. Endocrinol. 349, 138–144. 10.1016/j.mce.2011.10.014 22051447

[B99] Chen L. Xie Y. Fan J. Sui L. Xu Y. Zhang N.. (2015). HCG induces beta1,4-GalT I expression and promotes embryo implantation. Int. J. Clin. Exp. Pathol. 8, 4673–4683. 26191157 PMC4503029

[B100] Cheung K. L. Lafayette R. A. (2013). Renal physiology of pregnancy. Adv. Chronic Kidney Dis. 20, 209–214. 10.1053/j.ackd.2013.01.012 23928384 PMC4089195

[B101] Chinnathambi V. Blesson C. S. Vincent K. L. Saade G. R. Hankins G. D. Yallampalli C.. (2014). Elevated testosterone levels during rat pregnancy cause hypersensitivity to angiotensin II and attenuation of endothelium-dependent vasodilation in uterine arteries. Hypertension 64, 405–414. 10.1161/HYPERTENSIONAHA.114.03283 24842922 PMC4096063

[B102] Chung E. Yeung F. Leinwand L. A. (2012). Akt and MAPK signaling mediate pregnancy-induced cardiac adaptation. J Appl. Physiol. (1985) 112, 1564–1575. 10.1152/japplphysiol.00027.2012 22345431 PMC3362236

[B103] Chung W. K. Belfi K. Chua M. Wiley J. Mackintosh R. Nicolson M.. (1998). Heterozygosity for Lep(ob) or Lep(rdb) affects body composition and leptin homeostasis in adult mice. Am. J. Physiol. 274, R985–R990. 9575960 10.1152/ajpregu.1998.274.4.R985

[B104] Clarke A. G. Kendall M. D. (1994). The thymus in pregnancy: the interplay of neural, endocrine and immune influences. Immunol. Today 15, 545–551. 10.1016/0167-5699(94)90212-7 7802926

[B105] Clementi C. Tripurani S. K. Large M. J. Edson M. A. Creighton C. J. Hawkins S. M.. (2013). Activin-like kinase 2 functions in peri-implantation uterine signaling in mice and humans. PLoS Genet. 9:e1003863. 10.1371/journal.pgen.1003863 24244176 PMC3828128

[B106] Comai S. Ochoa-Sanchez R. Dominguez-Lopez S. Bambico F. R. Gobbi G. (2015). Melancholic-Like behaviors and circadian neurobiological abnormalities in melatonin MT1 receptor knockout mice. Int. J. Neuropsychopharmacol. 18. 10.1093/ijnp/pyu075 25638817 PMC4360238

[B107] Conrad K. P. Debrah D. O. Novak J. Danielson L. A. Shroff S. G. (2004). Relaxin modifies systemic arterial resistance and compliance in conscious, nonpregnant rats. Endocrinology 145, 3289–3296. 10.1210/en.2003-1612 15198972

[B108] Contreras-Alcantara S. Baba K. Tosini G. (2010). Removal of melatonin receptor type 1 induces insulin resistance in the mouse. Obesity (Silver. Spring). 18, 1861–1863. 10.1038/oby.2010.24 20168308 PMC2929321

[B109] Contreras G. Gutiérrez M. Beroíza T. Fantín A. Oddó H. Villarroel L.. (1991). Ventilatory drive and respiratory muscle function in pregnancy. Am. Rev. Respir. Dis. 144, 837–841. 10.1164/ajrccm/144.4.837 1928958

[B110] Costa M. A. (2016). The endocrine function of human placenta: an overview. Reprod. Biomed. Online 32, 14–43. 10.1016/j.rbmo.2015.10.005 26615903

[B111] Costrini N. V. Kalkhoff R. K. (1971). Relative effects of pregnancy, estradiol, and progesterone on plasma insulin and pancreatic islet insulin secretion. J. Clin. Invest. 50, 992–999. 10.1172/JCI106593 4928265 PMC292019

[B112] Coya R. Martul P. Algorta J. Aniel-Quiroga M. A. Busturia M. A. Señarís R. (2006). Effect of leptin on the regulation of placental hormone secretion in cultured human placental cells. Gynecol. Endocrinol. 22, 620–626. 10.1080/09513590601012587 17145648

[B113] Crocker I. Kaur M. Hosking D. J. Baker P. N. (2002). Rescue of trophoblast apoptosis by parathyroid hormone-related protein. BJOG 109, 218–220. 10.1111/j.1471-0528.2002.01033.x 11888106

[B114] Cruz M. A. Gallardo V. Miguel P. Carrasco G. Gonzalez C. (1997). Serotonin-induced vasoconstriction is mediated by thromboxane release and action in the human fetal-placental circulation. Placenta 18, 197–204. 10.1016/S0143-4004(97)90093-X 9089782

[B115] Díaz P. Powell T. L. Jansson T. (2014). The role of placental nutrient sensing in maternal-fetal resource allocation. Biol. Reprod. 91:82. 10.1095/biolreprod.114.121798 25122064 PMC4435028

[B116] Da Costa T. H. Taylor K. Ilic V. Williamson D. H. (1995). Regulation of milk lipid secretion: effects of oxytocin, prolactin and ionomycin on triacylglycerol release from rat mammary gland slices. Biochem. J. 308(Pt 3), 975–981. 10.1042/bj3080975 8948458 PMC1136818

[B117] Dai G. Lu L. Tang S. Peal M. J. Soares M. J. (2002). Prolactin family miniarray: a tool for evaluating uteroplacental-trophoblast endocrine cell phenotypes. Reproduction 124, 755–765. 10.1530/rep.0.1240755 12530913

[B118] Dai S. Q. Yu L. P. Shi X. Wu H. Shao P. Yin G. Y.. (2014). Serotonin regulates osteoblast proliferation and function *in vitro*. Braz. J. Med. Biol. Res. 47, 759–765. 10.1590/1414-431X20143565 25098615 PMC4143203

[B119] Danielson L. A. Sherwood O. D. Conrad K. P. (1999). Relaxin is a potent renal vasodilator in conscious rats. J. Clin. Invest. 103, 525–533. 10.1172/JCI5630 10021461 PMC408107

[B120] Datta N. S. Chen C. Berry J. E. Mccauley L. K. (2005). PTHrP signaling targets cyclin D1 and induces osteoblastic cell growth arrest. J. Bone Miner. Res. 20, 1051–1064. 10.1359/JBMR.050106 15883646

[B121] Davison J. M. Dunlop W. (1980). Renal hemodynamics and tubular function normal human pregnancy. Kidney Int. 18, 152–161. 10.1038/ki.1980.124 7003196

[B122] Dean M. Hunt J. Mcdougall L. Rose J. (2014). Uterine glycogen metabolism in mink during estrus, embryonic diapause and pregnancy. J. Reprod. Dev. 60, 438–446. 10.1262/jrd.2014-013 25225159 PMC4284318

[B123] Deblon N. Veyrat-Durebex C. Bourgoin L. Caillon A. Bussier A. L. Petrosino S.. (2011). Mechanisms of the anti-obesity effects of oxytocin in diet-induced obese rats. PLoS ONE 6:e25565. 10.1371/journal.pone.0025565 21980491 PMC3181274

[B124] Debrah D. O. Debrah J. E. Haney J. L. Mcguane J. T. Sacks M. S. Conrad K. P.. (2011). Relaxin regulates vascular wall remodeling and passive mechanical properties in mice. J. Appl. Physiol. (1985) 111, 260–271. 10.1152/japplphysiol.00845.2010 21551018 PMC3137537

[B125] Debrah D. O. Novak J. Matthews J. E. Ramirez R. J. Shroff S. G. Conrad K. P. (2006). Relaxin is essential for systemic vasodilation and increased global arterial compliance during early pregnancy in conscious rats. Endocrinology 147, 5126–5131. 10.1210/en.2006-0567 16873529

[B126] Declerck C. H. Boone C. Kiyonari T. (2010). Oxytocin and cooperation under conditions of uncertainty: the modulating role of incentives and social information. Horm. Behav. 57, 368–374. 10.1016/j.yhbeh.2010.01.006 20080100

[B127] De Dreu C. K. Greer L. L. Handgraaf M. J. Shalvi S. Van Kleef G. A. Baas M.. (2010). The neuropeptide oxytocin regulates parochial altruism in intergroup conflict among humans. Science 328, 1408–1411. 10.1126/science.1189047 20538951

[B128] Del Rincon J. P. Iida K. Gaylinn B. D. Mccurdy C. E. Leitner J. W. Barbour L. A.. (2007). Growth hormone regulation of p85alpha expression and phosphoinositide 3-kinase activity in adipose tissue: mechanism for growth hormone-mediated insulin resistance. Diabetes 56, 1638–1646. 10.2337/db06-0299 17363744

[B129] Denicolo G. Morris S. T. Kenyon P. R. Morel P. C. Parkinson T. J. (2008). Melatonin-improved reproductive performance in sheep bred out of season. Anim. Reprod. Sci. 109, 124–133. 10.1016/j.anireprosci.2007.10.012 18082341

[B130] De Pedro M. A. Morán J. Díaz I. Murias L. Fernández-Plaza C. Gonzále C.. (2015). Circadian Kisspeptin expression in human term placenta. Placenta 36, 1337–1339. 10.1016/j.placenta.2015.09.009 26422423

[B131] Dill R. Walker A. M. (2017). Role of prolactin in promotion of immune cell migration into the mammary gland. J. Mammary Gland Biol. Neoplasia 22, 13–26. 10.1007/s10911-016-9369-0 27900586 PMC5313375

[B132] Ding H. Zhang G. Sin K. W. Liu Z. Lin R. K. Li M.. (2017). Activin A induces skeletal muscle catabolism via p38beta mitogen-activated protein kinase. J. Cachexia Sarcopenia Muscle 8, 202–212. 10.1002/jcsm.12145 27897407 PMC5377410

[B133] Di W. L. Lachelin G. C. Mcgarrigle H. H. Thomas N. S. Becker D. L. (2001). Oestriol and oestradiol increase cell to cell communication and connexin43 protein expression in human myometrium. Mol. Hum. Reprod. 7, 671–679. 10.1093/molehr/7.7.671 11420391

[B134] Dominici F. P. Argentino D. P. Muñoz M. C. Miquet J. G. Sotelo A. I. Turyn D. (2005). Influence of the crosstalk between growth hormone and insulin signalling on the modulation of insulin sensitivity. Growth Horm. IGF Res. 15, 324–336. 10.1016/j.ghir.2005.07.001 16112592

[B135] Dominici F. P. Cifone D. Bartke A. Turyn D. (1999). Loss of sensitivity to insulin at early events of the insulin signaling pathway in the liver of growth hormone-transgenic mice. J. Endocrinol. 161, 383–392. 10.1677/joe.0.1610383 10333541

[B136] Douglas A. J. Johnstone L. E. Leng G. (2007). Neuroendocrine mechanisms of change in food intake during pregnancy: a potential role for brain oxytocin. Physiol. Behav. 91, 352–365. 10.1016/j.physbeh.2007.04.012 17512024

[B137] Drynda R. Peters C. J. Jones P. M. Bowe J. E. (2015). The role of non-placental signals in the adaptation of islets to pregnancy. Horm. Metab. Res. 47, 64–71. 10.1055/s-0034-1395691 25506682

[B138] Dunbar M. E. Dann P. Brown C. W. Van Houton J. Dreyer B. Philbrick W. P.. (2001). Temporally regulated overexpression of parathyroid hormone-related protein in the mammary gland reveals distinct fetal and pubertal phenotypes. J. Endocrinol. 171, 403–416. 10.1677/joe.0.1710403 11739006

[B139] Duval C. Dilworth M. R. Tunster S. J. Kimber S. J. Glazier J. D. (2017). PTHrP is essential for normal morphogenetic and functional development of the murine placenta. Dev. Biol. 430, 325–336. 10.1016/j.ydbio.2017.08.033 28864069

[B140] Edey L. F. Georgiou H. O'dea K. P. Mesiano S. Herbert B. R. Lei K.. (2018). Progesterone, the maternal immune system and the onset of parturition in the mouse. Biol. Reprod. 98, 376–395. 10.1093/biolre/iox146 29145579

[B141] Einspanier A. Lieder K. Husen B. Ebert K. Lier S. Einspanier R.. (2009). Relaxin supports implantation and early pregnancy in the marmoset monkey. Ann. N. Y. Acad. Sci. 1160, 140–146. 10.1111/j.1749-6632.2009.03947.x 19416176

[B142] Elabd S. K. Sabry I. Hassan W. B. Nour H. Zaky K. (2007). Possible neuroendocrine role for oxytocin in bone remodeling. Endocr. Regul. 41, 131–141. 18257653

[B143] El-Hashash A. H. Kimber S. J. (2006). PTHrP induces changes in cell cytoskeleton and E-cadherin and regulates Eph/Ephrin kinases and RhoGTPases in murine secondary trophoblast cells. Dev. Biol. 290, 13–31. 10.1016/j.ydbio.2005.10.010 16375886

[B144] Elling S. V. Powell F. C. (1997). Physiological changes in the skin during pregnancy. Clin. Dermatol. 15, 35–43. 10.1016/S0738-081X(96)00108-3 9034654

[B145] Elsheikh A. Creatsas G. Mastorakos G. Milingos S. Loutradis D. Michalas S. (2001). The renin-aldosterone system during normal and hypertensive pregnancy. Arch. Gynecol. Obstet. 264, 182–185. 10.1007/s004040000104 11205704

[B146] Emly J. F. Gregory J. Bowden S. J. Ahmed A. Whittle M. J. Rushton D. I.. (1994). Immunohistochemical localization of parathyroid hormone-related protein (PTHrP) in human term placenta and membranes. Placenta 15, 653–660. 10.1016/S0143-4004(05)80411-4 7824450

[B147] Enright W. J. Chapin L. T. Moseley W. M. Tucker H. A. (1988). Effects of infusions of various doses of bovine growth hormone-releasing factor on growth hormone and lactation in Holstein cows. J. Dairy Sci. 71, 99–108. 10.3168/jds.S0022-0302(88)79530-2 3131403

[B148] Enright W. J. Chapin L. T. Moseley W. M. Zinn S. A. Kamdar M. B. Krabill L. F.. (1989). Effects of infusions of various doses of bovine growth hormone-releasing factor on blood hormones and metabolites in lactating Holstein cows. J. Endocrinol. 122, 671–679. 10.1677/joe.0.1220671 2509616

[B149] Enright W. J. Chapin L. T. Moseley W. M. Zinn S. A. Tucker H. A. (1986). Growth hormone-releasing factor stimulates milk production and sustains growth hormone release in Holstein cows. J. Dairy Sci. 69, 344–351. 10.3168/jds.S0022-0302(86)80412-X 3084602

[B150] Ernst S. Demirci C. Valle S. Velazquez-Garcia S. Garcia-Ocaña A. (2011). Mechanisms in the adaptation of maternal beta-cells during pregnancy. Diabetes Manag. (Lond). 1, 239–248. 10.2217/dmt.10.24 21845205 PMC3155205

[B151] Eta E. Ambrus G. Rao C. V. (1994). Direct regulation of human myometrial contractions by human chorionic gonadotropin. J. Clin. Endocrinol. Metab. 79, 1582–1586. 7989459 10.1210/jcem.79.6.7989459

[B152] Etienne M. Bonneau M. Kann G. Deletang F. (1992). Effects of administration of growth hormone-releasing factor to sows during late gestation on growth hormone secretion, reproductive traits, and performance of progeny from birth to 100 kilograms live weight. J. Anim. Sci. 70, 2212–2220. 10.2527/1992.7072212x 1644696

[B153] Everson G. T. (1992). Gastrointestinal motility in pregnancy. Gastroenterol. Clin. North Am. 21, 751–776. 1478733

[B154] Fang X. Wong S. Mitchell B. F. (1997). Effects of RU486 on estrogen, progesterone, oxytocin, and their receptors in the rat uterus during late gestation. Endocrinology 138, 2763–2768. 10.1210/endo.138.7.5247 9202215

[B155] Farmer C. Dubreuil P. Pelletier G. Petitclerc D. Gaudreau P. Brazeau P. (1991). Effects of active immunization against somatostatin (SRIF) and/or injections of growth hormone-releasing factor (GRF) during gestation on hormonal and metabolic profiles in sows. Domest. Anim. Endocrinol. 8, 415–422. 10.1016/0739-7240(91)90009-9 1684146

[B156] Farmer C. Petitclerc D. Pelletier G. Brazeau P. (1992). Lactation performance of sows injected with growth hormone-releasing factor during gestation and(or) lactation. J. Anim. Sci. 70, 2636–2642. 10.2527/1992.7092636x 1399876

[B157] Farmer C. Robert S. Matte J. J. (1996). Lactation performance of sows fed a bulky diet during gestation and receiving growth hormone-releasing factor during lactation. J. Anim. Sci. 74, 1298–1306. 10.2527/1996.7461298x 8791202

[B158] Fecteau K. A. Eiler H. (2001). Placenta detachment: unexpected high concentrations of 5-hydroxytryptamine (serotonin) in fetal blood and its mitogenic effect on placental cells in bovine. Placenta 22, 103–110. 10.1053/plac.2000.0596 11162359

[B159] Feng S. Bogatcheva N. V. Kamat A. A. Truong A. Agoulnik A. I. (2006). Endocrine effects of relaxin overexpression in mice. Endocrinology 147, 407–414. 10.1210/en.2005-0626 16223865

[B160] Ferguson J. N. Young L. J. Hearn E. F. Matzuk M. M. Insel T. R. Winslow J. T. (2000). Social amnesia in mice lacking the oxytocin gene. Nat. Genet. 25, 284–288. 10.1038/77040 10888874

[B161] Ferris C. F. Foote K. B. Meltser H. M. Plenby M. G. Smith K. L. Insel T. R. (1992). Oxytocin in the amygdala facilitates maternal aggression. Ann. N. Y. Acad. Sci. 652, 456–457. 10.1111/j.1749-6632.1992.tb34382.x 1626847

[B162] Fettke F. Schumacher A. Canellada A. Toledo N. Bekeredjian-Ding I. Bondt A.. (2016). Maternal and fetal mechanisms of B cell regulation during pregnancy: human chorionic gonadotropin stimulates B cells to Produce IL-10 while alpha-fetoprotein drives them into apoptosis. Front. Immunol. 7:495. 10.3389/fimmu.2016.00495 28008329 PMC5144100

[B163] Fliegner D. Schubert C. Penkalla A. Witt H. Kararigas G. Dworatzek E.. (2010). Female sex and estrogen receptor-beta attenuate cardiac remodeling and apoptosis in pressure overload. Am. J. Physiol. Regul. Integr. Comp. Physiol. 298, R1597–R1606. 10.1152/ajpregu.00825.2009 20375266

[B164] Flores-Espinosa P. Preciado-Martínez E. Mejía-Salvador A. Sedano-González G. Bermejo-Martínez L. Parra-Covarruvias A.. (2017). Selective immuno-modulatory effect of prolactin upon pro-inflammatory response in human fetal membranes. J. Reprod. Immunol. 123, 58–64. 10.1016/j.jri.2017.09.004 28938125

[B165] Florio P. Lombardo M. Gallo R. Di Carlo C. Sutton S. Genazzani A. R.. (1996). Activin A, corticotropin-releasing factor and prostaglandin F2 alpha increase immunoreactive oxytocin release from cultured human placental cells. Placenta 17, 307–311. 10.1016/S0143-4004(96)90054-5 8829213

[B166] Fournier T. Guibourdenche J. Evain-Brion D. (2015). Review: hCGs: different sources of production, different glycoforms and functions. Placenta 36(Suppl. 1), S60–S65. 10.1016/j.placenta.2015.02.002 25707740

[B167] Fowden A. L. Giussani D. A. Forhead A. J. (2006). Intrauterine programming of physiological systems: causes and consequences. Physiology (Bethesda). 21, 29–37. 10.1152/physiol.00050.2005 16443820

[B168] Fowden A. L. Moore T. (2012). Maternal-fetal resource allocation: co-operation and conflict. Placenta 33(Suppl. 2), e11–e15. 10.1016/j.placenta.2012.05.002 22652046

[B169] Fowler P. A. Evans L. W. Groome N. P. Templeton A. Knight P. G. (1998). A longitudinal study of maternal serum inhibin-A, inhibin-B, activin-A, activin-AB, pro-alphaC and follistatin during pregnancy. Hum. Reprod. 13, 3530–3536. 10.1093/humrep/13.12.3530 9886545

[B170] Freemark M. (2010). Placental hormones and the control of fetal growth. J. Clin. Endocrinol. Metab. 95, 2054–2057. 10.1210/jc.2010-0517 20444932

[B171] Freemark M. Avril I. Fleenor D. Driscoll P. Petro A. Opara E.. (2002). Targeted deletion of the PRL receptor: effects on islet development, insulin production, and glucose tolerance. Endocrinology 143, 1378–1385. 10.1210/endo.143.4.8722 11897695

[B172] Freemark M. Fleenor D. Driscoll P. Binart N. Kelly P. (2001). Body weight and fat deposition in prolactin receptor-deficient mice. Endocrinology 142, 532–537. 10.1210/endo.142.2.7979 11159821

[B173] Frise C. Noori M. Williamson C. (2013). Severe metabolic alkalosis in pregnancy. Obstet. Med. 6, 138–140. 10.1258/om.2012.120030 27708709 PMC5032930

[B174] Fudge N. J. Kovacs C. S. (2010). Pregnancy up-regulates intestinal calcium absorption and skeletal mineralization independently of the vitamin D receptor. Endocrinology 151, 886–895. 10.1210/en.2009-1010 20051486

[B175] Fujinaka Y. Sipula D. Garcia-Ocaña A. Vasavada R. C. (2004). Characterization of mice doubly transgenic for parathyroid hormone-related protein and murine placental lactogen: a novel role for placental lactogen in pancreatic beta-cell survival. Diabetes 53, 3120–3130. 10.2337/diabetes.53.12.3120 15561942

[B176] Fungfuang W. Terada M. Komatsu N. Moon C. Saito T. R. (2013). Effects of estrogen on food intake, serum leptin levels and leptin mRNA expression in adipose tissue of female rats. Lab. Anim. Res. 29, 168–173. 10.5625/lar.2013.29.3.168 24106512 PMC3791351

[B177] Gallacher S. J. Fraser W. D. Owens O. J. Dryburgh F. J. Logue F. C. Jenkins A.. (1994). Changes in calciotrophic hormones and biochemical markers of bone turnover in normal human pregnancy. Eur. J. Endocrinol. 131, 369–374. 10.1530/eje.0.1310369 7921225

[B178] Gallego M. I. Binart N. Robinson G. W. Okagaki R. Coschigano K. T. Perry J.. (2001). Prolactin, growth hormone, and epidermal growth factor activate Stat5 in different compartments of mammary tissue and exert different and overlapping developmental effects. Dev. Biol. 229, 163–175. 10.1006/dbio.2000.9961 11133161

[B179] Galosy S. S. Talamantes F. (1995). Luteotropic actions of placental lactogens at midpregnancy in the mouse. Endocrinology 136, 3993–4003. 10.1210/endo.136.9.7649108 7649108

[B180] Garcia-Ruíz G. Flores-Espinosa P. Preciado-Martínez E. Bermejo-Martínez L. Espejel-Nuñez A. Estrada-Gutierrez G.. (2015). *In vitro* progesterone modulation on bacterial endotoxin-induced production of IL-1beta, TNFalpha, IL-6, IL-8, IL-10, MIP-1alpha, and MMP-9 in pre-labor human term placenta. Reprod. Biol. Endocrinol. 13:115. 10.1186/s12958-015-0111-3 26446923 PMC4596542

[B181] Gimeno M. F. Landa A. Sterin-Speziale N. Cardinali D. P. Gimeno A. L. (1980). Melatonin blocks *in vitro* generation of prostaglandin by the uterus and hypothalamus. Eur. J. Pharmacol. 62, 309–317. 10.1016/0014-2999(80)90098-9 6102921

[B182] Goh B. C. Singhal V. Herrera A. J. Tomlinson R. E. Kim S. Faugere M. C.. (2017). Activin receptor type 2A (ACVR2A) functions directly in osteoblasts as a negative regulator of bone mass. J. Biol. Chem. 292, 13809–13822. 10.1074/jbc.M117.782128 28659341 PMC5566533

[B183] Goldsmith L. T. Weiss G. Palejwala S. Plant T. M. Wojtczuk A. Lambert W. C.. (2004). Relaxin regulation of endometrial structure and function in the rhesus monkey. Proc. Natl. Acad. Sci. U.S.A. 101, 4685–4689. 10.1073/pnas.0400776101 15070778 PMC384807

[B184] Golightly E. Jabbour H. N. Norman J. E. (2011). Endocrine immune interactions in human parturition. Mol. Cell. Endocrinol. 335, 52–59. 10.1016/j.mce.2010.08.005 20708653

[B185] González-Candia A. Veliz M. Araya C. Quezada S. Ebensperger G. Seron-Ferre M.. (2016). Potential adverse effects of antenatal melatonin as a treatment for intrauterine growth restriction: findings in pregnant sheep. Am J Obstet Gynecol 215, 245 e241–245 e247. 10.1016/j.ajog.2016.02.040 26902986

[B186] Goodman H. M. Tai L. R. Ray J. Cooke N. E. Liebhaber S. A. (1991). Human growth hormone variant produces insulin-like and lipolytic responses in rat adipose tissue. Endocrinology 129, 1779–1783. 10.1210/endo-129-4-1779 1915067

[B187] Gooi J. H. Richardson M. L. Jelinic M. Girling J. E. Wlodek M. E. Tare M.. (2013). Enhanced uterine artery stiffness in aged pregnant relaxin mutant mice is reversed with exogenous relaxin treatment. Biol. Reprod. 89:18. 10.1095/biolreprod.113.108118 23718984

[B188] Gopalakrishnan K. Mishra J. S. Chinnathambi V. Vincent K. L. Patrikeev I. Motamedi M.. (2016). Elevated testosterone reduces uterine blood flow, spiral artery elongation, and placental oxygenation in pregnant rats. Hypertension 67, 630–639. 10.1161/HYPERTENSIONAHA.115.06946 26781277 PMC4752400

[B189] Goyvaerts L. Schraenen A. Schuit F. (2016). Serotonin competence of mouse beta cells during pregnancy. Diabetologia 59, 1356–1363. 10.1007/s00125-016-3951-2 27056372

[B190] Greening D. W. Nguyen H. P. Evans J. Simpson R. J. Salamonsen L. A. (2016). Modulating the endometrial epithelial proteome and secretome in preparation for pregnancy: the role of ovarian steroid and pregnancy hormones. J. Proteomics 144, 99–112. 10.1016/j.jprot.2016.05.026 27262222

[B191] Gregg C. (2009). Pregnancy, prolactin and white matter regeneration. J. Neurol. Sci. 285, 22–27. 10.1016/j.jns.2009.06.040 19608204

[B192] Grès S. Canteiro S. Mercader J. Carpene C. (2013). Oxidation of high doses of serotonin favors lipid accumulation in mouse and human fat cells. Mol. Nutr. Food Res. 57, 1089–1099. 10.1002/mnfr.201200681 23390020

[B193] Groba C. Mayerl S. Van Mullem A. A. Visser T. J. Darras V. M. Habenicht A. J.. (2013). Hypothyroidism compromises hypothalamic leptin signaling in mice. Mol. Endocrinol. 27, 586–597. 10.1210/me.2012-1311 23518925 PMC5416808

[B194] Groen B. Van Der Wijk A. E. Van Den Berg P. P. Lefrandt J. D. Van Den Berg G. Sollie K. M.. (2015). Immunological Adaptations to Pregnancy in Women with Type 1 Diabetes. Sci. Rep. 5:13618. 10.1038/srep13618 26391604 PMC4585728

[B195] Groskopf J. C. Syu L. J. Saltiel A. R. Linzer D. I. (1997). Proliferin induces endothelial cell chemotaxis through a G protein-coupled, mitogen-activated protein kinase-dependent pathway. Endocrinology 138, 2835–2840. 10.1210/endo.138.7.5276 9202225

[B196] Gulinello M. Gong Q. H. Smith S. S. (2002). Progesterone withdrawal increases the alpha4 subunit of the GABA(A) receptor in male rats in association with anxiety and altered pharmacology-a comparison with female rats. Neuropharmacology 43, 701–714. 10.1016/S0028-3908(02)00171-5 12367616 PMC2887344

[B197] Gutkowska J. Jankowski M. (2012). Oxytocin revisited: its role in cardiovascular regulation. J. Neuroendocrinol. 24, 599–608. 10.1111/j.1365-2826.2011.02235.x 21981277

[B198] Habiger V. W. (1975). Serotonin effect on the fetus and the feto-maternal relationship in the rat. Arzneimittelforschung. 25, 626–632. 1174077

[B199] Hadden C. Fahmi T. Cooper A. Savenka A. V. Lupashin V. V. Roberts D. J.. (2017). Serotonin transporter protects the placental cells against apoptosis in caspase 3-independent pathway. J. Cell. Physiol. 232, 3520–3529. 10.1002/jcp.25812 28109119 PMC5522371

[B200] Hadden D. R. Mclaughlin C. (2009). Normal and abnormal maternal metabolism during pregnancy. Semin. Fetal Neonatal Med. 14, 66–71. 10.1016/j.siny.2008.09.004 18986856

[B201] Haig D. (2008). Placental growth hormone-related proteins and prolactin-related proteins. Placenta 29(Suppl. A), S36–S41. 10.1016/j.placenta.2007.09.010 17981323

[B202] Hales C. N. Barker D. J. (2001). The thrifty phenotype hypothesis. Br. Med. Bull. 60, 5–20. 10.1093/bmb/60.1.5 11809615

[B203] Handwerger S. Richards R. G. Markoff E. (1992). The physiology of decidual prolactin and other decidual protein hormones. Trends Endocrinol. Metab. 3, 91–95. 10.1016/1043-2760(92)90019-W 18407085

[B204] Harris L. K. Crocker I. P. Baker P. N. Aplin J. D. Westwood M. (2011). IGF2 actions on trophoblast in human placenta are regulated by the insulin-like growth factor 2 receptor, which can function as both a signaling and clearance receptor. Biol. Reprod. 84, 440–446. 10.1095/biolreprod.110.088195 20980691 PMC3043127

[B205] Hart I. C. Chadwick P. M. James S. Simmonds A. D. (1985). Effect of intravenous bovine growth hormone or human pancreatic growth hormone-releasing factor on milk production and plasma hormones and metabolites in sheep. J. Endocrinol. 105, 189–196. 10.1677/joe.0.1050189 3921646

[B206] Hauguel-De Mouzon S. Lepercq J. Catalano P. (2006). The known and unknown of leptin in pregnancy. Am. J. Obstet. Gynecol. 194, 1537–1545. 10.1016/j.ajog.2005.06.064 16731069

[B207] Haynes M. P. Sinha D. Russell K. S. Collinge M. Fulton D. Morales-Ruiz M.. (2000). Membrane estrogen receptor engagement activates endothelial nitric oxide synthase via the PI3-kinase-Akt pathway in human endothelial cells. Circ. Res. 87, 677–682. 10.1161/01.RES.87.8.677 11029403

[B208] Hearn J. P. Gidley-Baird A. A. Hodges J. K. Summers P. M. Webley G. E. (1988). Embryonic signals during the peri-implantation period in primates. J. Reprod. Fertil. Suppl. 36, 49–58. 3142993

[B209] Hegewald M. J. Crapo R. O. (2011). Respiratory physiology in pregnancy. Clin. Chest Med. 32, 1–13, vii. 10.1016/j.ccm.2010.11.001 21277444

[B210] Hellmeyer L. Ziller V. Anderer G. Ossendorf A. Schmidt S. Hadji P. (2006). Biochemical markers of bone turnover during pregnancy: a longitudinal study. Exp. Clin. Endocrinol. Diabetes 114, 506–510. 10.1055/s-2006-951627 17115348

[B211] Henson M. C. Castracane V. D. O'neil J. S. Gimpel T. Swan K. F. Green A. E.. (1999). Serum leptin concentrations and expression of leptin transcripts in placental trophoblast with advancing baboon pregnancy. J. Clin. Endocrinol. Metab. 84, 2543–2549. 10.1210/jc.84.7.2543 10404834

[B212] Hernández-Castellano L. E. Hernandez L. L. Weaver S. Bruckmaier R. M. (2017). Increased serum serotonin improves parturient calcium homeostasis in dairy cows. J. Dairy Sci. 100, 1580–1587. 10.3168/jds.2016-11638 27988124

[B213] Herreboudt A. M. Kyle V. R. Lawrence J. Doran J. Colledge W. H. (2015). Kiss1 mutant placentas show normal structure and function in the mouse. Placenta 36, 52–58. 10.1016/j.placenta.2014.10.016 25468546 PMC4302219

[B214] Hershberger M. E. Tuan R. S. (1998). Placental 57-kDa Ca(2+)-binding protein: regulation of expression and function in trophoblast calcium transport. Dev. Biol. 199, 80–92. 10.1006/dbio.1998.8926 9676194

[B215] Hershman J. M. Kojima A. Friesen H. G. (1973). Effect of thyrotropin-releasing hormone on human pituitary thyrotropin, prolactin, placental lactogen, and chorionic thyrotropin. J. Clin. Endocrinol. Metab. 36, 497–501. 10.1210/jcem-36-3-497 4631064

[B216] Highman T. J. Friedman J. E. Huston L. P. Wong W. W. Catalano P. M. (1998). Longitudinal changes in maternal serum leptin concentrations, body composition, and resting metabolic rate in pregnancy. Am. J. Obstet. Gynecol. 178, 1010–1015. 10.1016/S0002-9378(98)70540-X 9609576

[B217] Hirota Y. Anai T. Miyakawa I. (1997). Parathyroid hormone-related protein levels in maternal and cord blood. Am. J. Obstet. Gynecol. 177, 702–706. 10.1016/S0002-9378(97)70167-4 9322645

[B218] Hisamoto K. Ohmichi M. Kurachi H. Hayakawa J. Kanda Y. Nishio Y.. (2001). Estrogen induces the Akt-dependent activation of endothelial nitric-oxide synthase in vascular endothelial cells. J. Biol. Chem. 276, 3459–3467. 10.1074/jbc.M005036200 11044445

[B219] Hisaw F. L. Hisaw F. L. Jr. Dawson A. B. (1967). Effects of relaxin on the endothelium of endometrial blood vessels in monkeys (*Macaca mulatta*). Endocrinology 81, 375–385. 10.1210/endo-81-2-375 4952013

[B220] Hoekzema E. Barba-Müller E. Pozzobon C. Picado M. Lucco F. García-García D.. (2017). Pregnancy leads to long-lasting changes in human brain structure. Nat. Neurosci. 20, 287–296. 10.1038/nn.4458 27991897

[B221] Horber F. F. Haymond M. W. (1990). Human growth hormone prevents the protein catabolic side effects of prednisone in humans. J. Clin. Invest. 86, 265–272. 10.1172/JCI114694 2195062 PMC296716

[B222] Horikoshi Y. Matsumoto H. Takatsu Y. Ohtaki T. Kitada C. Usuki S.. (2003). Dramatic elevation of plasma metastin concentrations in human pregnancy: metastin as a novel placenta-derived hormone in humans. J. Clin. Endocrinol. Metab. 88, 914–919. 10.1210/jc.2002-021235 12574233

[B223] Horseman N. D. Zhao W. Montecino-Rodriguez E. Tanaka M. Nakashima K. Engle S. J.. (1997). Defective mammopoiesis, but normal hematopoiesis, in mice with a targeted disruption of the prolactin gene. EMBO J. 16, 6926–6935. 10.1093/emboj/16.23.6926 9384572 PMC1170296

[B224] Huang C. Snider F. Cross J. C. (2009). Prolactin receptor is required for normal glucose homeostasis and modulation of beta-cell mass during pregnancy. Endocrinology 150, 1618–1626. 10.1210/en.2008-1003 19036882

[B225] Hudon Thibeault A. A. Laurent L. Vo Duy S. Sauve S. Caron P. Guillemette C.. (2017). Fluoxetine and its active metabolite norfluoxetine disrupt estrogen synthesis in a co-culture model of the feto-placental unit. Mol. Cell. Endocrinol. 442, 32–39. 10.1016/j.mce.2016.11.021 27890559

[B226] Hughes C. K. Xie M. M. Mccoski S. R. Ealy A. D. (2017). Activities for leptin in bovine trophoblast cells. Domest. Anim. Endocrinol. 58, 84–89. 10.1016/j.domaniend.2016.09.001 27743526

[B227] Ibrahim H. S. Omar E. Froemming G. R. Singh H. J. (2013). Leptin increases blood pressure and markers of endothelial activation during pregnancy in rats. Biomed Res. Int. 2013, 298401. 10.1155/2013/298401 24167814 PMC3792531

[B228] Ishizuka T. Klepcyk P. Liu S. Panko L. Liu S. Gibbs E. M.. (1999). Effects of overexpression of human GLUT4 gene on maternal diabetes and fetal growth in spontaneous gestational diabetic C57BLKS/J Lepr(db/+) mice. Diabetes 48, 1061–1069. 10.2337/diabetes.48.5.1061 10331411

[B229] Islami D. Bischof P. Chardonnens D. (2003a). Modulation of placental vascular endothelial growth factor by leptin and hCG. Mol. Hum. Reprod. 9, 395–398. 10.1093/molehr/gag053 12802046

[B230] Islami D. Bischof P. Chardonnens D. (2003b). Possible interactions between leptin, gonadotrophin-releasing hormone (GnRH-I and II) and human chorionic gonadotrophin (hCG). Eur. J. Obstet. Gynecol. Reprod. Biol. 110, 169–175. 10.1016/S0301-2115(03)00185-4 12969578

[B231] Islam M. S. Morton N. M. Hansson A. Emilsson V. (1997). Rat insulinoma-derived pancreatic beta-cells express a functional leptin receptor that mediates a proliferative response. Biochem. Biophys. Res. Commun. 238, 851–855. 10.1006/bbrc.1997.7399 9325180

[B232] Iwasaki S. Nakazawa K. Sakai J. Kometani K. Iwashita M. Yoshimura Y.. (2005). Melatonin as a local regulator of human placental function. J. Pineal Res. 39, 261–265. 10.1111/j.1600-079X.2005.00244.x 16150106

[B233] Izquierdo A. López-Luna P. Ortega A. Romero M. Guitiérrez-Tarrés M. A. Arribas I.. (2006). The parathyroid hormone-related protein system and diabetic nephropathy outcome in streptozotocin-induced diabetes. Kidney Int. 69, 2171–2177. 10.1038/sj.ki.5000195 16783882

[B234] Jackson D. Volpert O. V. Bouck N. Linzer D. I. (1994). Stimulation and inhibition of angiogenesis by placental proliferin and proliferin-related protein. Science 266, 1581–1584. 10.1126/science.7527157 7527157

[B235] Jahnke G. Marr M. Myers C. Wilson R. Travlos G. Price C. (1999). Maternal and developmental toxicity evaluation of melatonin administered orally to pregnant Sprague-Dawley rats. Toxicol. Sci. 50, 271–279. 10.1093/toxsci/50.2.271 10478864

[B236] Jenkin G. Ward J. Loose J. Schneider-Kolsky M. Young R. Canny B.. (2001). Physiological and regulatory roles of activin A in late pregnancy. Mol. Cell. Endocrinol. 180, 131–138. 10.1016/S0303-7207(01)00504-4 11451582

[B237] Jiang C. W. Sarrel P. M. Lindsay D. C. Poole-Wilson P. A. Collins P. (1992). Progesterone induces endothelium-independent relaxation of rabbit coronary artery *in vitro*. Eur. J. Pharmacol. 211, 163–167. 10.1016/0014-2999(92)90524-8 1319340

[B238] Jobe S. O. Ramadoss J. Koch J. M. Jiang Y. Zheng J. Magness R. R. (2010). Estradiol-17beta and its cytochrome P450- and catechol-O-methyltransferase-derived metabolites stimulate proliferation in uterine artery endothelial cells: role of estrogen receptor-alpha versus estrogen receptor-beta. Hypertension 55, 1005–1011. 10.1161/HYPERTENSIONAHA.109.146399 20212268 PMC2876348

[B239] Jones R. L. Findlay J. K. Farnworth P. G. Robertson D. M. Wallace E. Salamonsen L. A. (2006). Activin A and inhibin A differentially regulate human uterine matrix metalloproteinases: potential interactions during decidualization and trophoblast invasion. Endocrinology 147, 724–732. 10.1210/en.2005-1183 16282351

[B240] Joshi P. A. Jackson H. W. Beristain A. G. Di Grappa M. A. Mote P. A. Clarke C. L.. (2010). Progesterone induces adult mammary stem cell expansion. Nature 465, 803–807. 10.1038/nature09091 20445538

[B241] Jo Y. S. Lee G. S. Nam S. Y. Kim S. J. (2015). Progesterone inhibits leptin-induced invasiveness of BeWo cells. Int. J. Med. Sci. 12, 773–779. 10.7150/ijms.11610 26516305 PMC4615237

[B242] Kaftanovskaya E. M. Huang Z. Lopez C. Conrad K. Agoulnik A. I. (2015). Conditional deletion of the relaxin receptor gene in cells of smooth muscle lineage affects lower reproductive tract in pregnant mice. Biol. Reprod. 92:91. 10.1095/biolreprod.114.127209 25715795 PMC4643956

[B243] Kalkwarf H. J. Specker B. L. (2002). Bone mineral changes during pregnancy and lactation. Endocrine 17, 49–53. 10.1385/ENDO:17:1:49 12014704

[B244] Kamat A. A. Feng S. Bogatcheva N. V. Truong A. Bishop C. E. Agoulnik A. I. (2004). Genetic targeting of relaxin and insulin-like factor 3 receptors in mice. Endocrinology 145, 4712–4720. 10.1210/en.2004-0515 15256493

[B245] Kane M. J. Angoa-Peréz M. Briggs D. I. Sykes C. E. Francescutti D. M. Rosenberg D. R.. (2012). Mice genetically depleted of brain serotonin display social impairments, communication deficits and repetitive behaviors: possible relevance to autism. PLoS ONE 7:e48975. 10.1371/journal.pone.0048975 23139830 PMC3490915

[B246] Kane N. Kelly R. Saunders P. T. Critchley H. O. (2009). Proliferation of uterine natural killer cells is induced by human chorionic gonadotropin and mediated via the mannose receptor. Endocrinology 150, 2882–2888. 10.1210/en.2008-1309 19196802 PMC2709965

[B247] Karaplis A. C. Luz A. Glowacki J. Bronson R. T. Tybulewicz V. L. Kronenberg H. M.. (1994). Lethal skeletal dysplasia from targeted disruption of the parathyroid hormone-related peptide gene. Genes Dev. 8, 277–289. 10.1101/gad.8.3.277 8314082

[B248] Keebaugh A. C. Barrett C. E. Laprairie J. L. Jenkins J. J. Young L. J. (2015). RNAi knockdown of oxytocin receptor in the nucleus accumbens inhibits social attachment and parental care in monogamous female prairie voles. Soc. Neurosci. 10, 561–570. 10.1080/17470919.2015.1040893 25874849 PMC4618772

[B249] Kendall M. D. Clarke A. G. (2000). The thymus in the mouse changes its activity during pregnancy: a study of the microenvironment. J. Anat. 197(Pt 3), 393–411. 10.1046/j.1469-7580.2000.19730393.x 11117626 PMC1468141

[B250] Keomanivong F. E. Lemley C. O. Camacho L. E. Yunusova R. Borowicz P. P. Caton J. S.. (2016). Influence of nutrient restriction and melatonin supplementation of pregnant ewes on maternal and fetal pancreatic digestive enzymes and insulin-containing clusters. Animal 10, 440–448. 10.1017/S1751731115002219 26549462

[B251] Khil L. Y. Jun H. S. Kwon H. Yoo J. K. Kim S. Notkins A. L.. (2007). Human chorionic gonadotropin is an immune modulator and can prevent autoimmune diabetes in NOD mice. Diabetologia 50, 2147–2155. 10.1007/s00125-007-0769-y 17676307

[B252] Kim C. Newton K. M. Knopp R. H. (2002). Gestational diabetes and the incidence of type 2 diabetes: a systematic review. Diabetes Care 25, 1862–1868. 10.2337/diacare.25.10.1862 12351492

[B253] Kim H. Toyofuku Y. Lynn F. C. Chak E. Uchida T. Mizukami H.. (2010). Serotonin regulates pancreatic beta cell mass during pregnancy. Nat. Med. 16, 804–808. 10.1038/nm.2173 20581837 PMC2921604

[B254] Kim J. K. (2009). Hyperinsulinemic-euglycemic clamp to assess insulin sensitivity *in vivo*. Methods Mol. Biol. 560, 221–238. 10.1007/978-1-59745-448-3_15 19504253

[B255] Kim M. N. Park M. N. Jung H. K. Cho C. Mayo K. E. Cho B. N. (2008). Changes in the reproductive function and developmental phenotypes in mice following intramuscular injection of an activin betaA-expressing plasmid. Reprod. Biol. Endocrinol. 6:63. 10.1186/1477-7827-6-63 19077325 PMC2639581

[B256] Kim P. (2016). Human maternal brain plasticity: adaptation to parenting. New Dir. Child Adolesc. Dev. 2016, 47–58. 10.1002/cad.20168 27589497 PMC5667351

[B257] Kim P. Strathearn L. Swain J. E. (2016). The maternal brain and its plasticity in humans. Horm. Behav. 77, 113–123. 10.1016/j.yhbeh.2015.08.001 26268151 PMC4724473

[B258] Kim S. C. Lee J. E. Kang S. S. Yang H. S. Kim S. S. An B. S. (2017). The regulation of oxytocin and oxytocin receptor in human placenta according to gestational age. J. Mol. Endocrinol. 59, 235–243. 10.1530/JME-16-0223 28694300

[B259] Kim S. H. Bennett P. R. Terzidou V. (2017). Advances in the role of oxytocin receptors in human parturition. Mol. Cell. Endocrinol. 449, 56–63. 10.1016/j.mce.2017.01.034 28119132

[B260] King J. C. (2000). Physiology of pregnancy and nutrient metabolism. Am. J. Clin. Nutr 71, 1218S–1225S. 10.1093/ajcn/71.5.1218s 10799394

[B261] Kirby B. J. Ardeshirpour L. Woodrow J. P. Wysolmerski J. J. Sims N. A. Karaplis A. C. (2011). Skeletal recovery after weaning does not require PTHrP. J. Bone Miner. Res. 26, 1242–1251. 10.1002/jbmr.339 21308774 PMC3179289

[B262] Kirwan J. P. Varastehpour A. Jing M. Presley L. Shao J. Friedman J. E.. (2004). Reversal of insulin resistance postpartum is linked to enhanced skeletal muscle insulin signaling. J. Clin. Endocrinol. Metab. 89, 4678–4684. 10.1210/jc.2004-0749 15356080

[B263] Kleiman A. Keats E. C. Chan N. G. Khan Z. A. (2013). Elevated IGF2 prevents leptin induction and terminal adipocyte differentiation in hemangioma stem cells. Exp. Mol. Pathol. 94, 126–136. 10.1016/j.yexmp.2012.09.023 23047069

[B264] Kobayashi K. Tsugami Y. Matsunaga K. Oyama S. Kuki C. Kumura H. (2016). Prolactin and glucocorticoid signaling induces lactation-specific tight junctions concurrent with beta-casein expression in mammary epithelial cells. Biochim. Biophys. Acta 1863, 2006–2016. 10.1016/j.bbamcr.2016.04.023 27130254

[B265] Koonce C. J. Frye C. A. (2013). Progesterone facilitates exploration, affective and social behaviors among wildtype, but not 5alpha-reductase Type 1 mutant, mice. Behav. Brain Res. 253, 232–239. 10.1016/j.bbr.2013.07.025 23886595 PMC3761366

[B266] Kota S. K. Gayatri K. Jammula S. Kota S. K. Krishna S. V. Meher L. K.. (2013). Endocrinology of parturition. Indian J. Endocrinol. Metab. 17, 50–59. 10.4103/2230-8210.107841 23776853 PMC3659907

[B267] Krajnc-Franken M. A. Van Disseldorp A. J. Koenders J. E. Mosselman S. Van Duin M. Gossen J. A. (2004). Impaired nipple development and parturition in LGR7 knockout mice. Mol. Cell. Biol. 24, 687–696. 10.1128/MCB.24.2.687-696.2004 14701741 PMC343807

[B268] Krutzén E. Olofsson P. Bäck S. E. Nilsson-Ehle P. (1992). Glomerular filtration rate in pregnancy: a study in normal subjects and in patients with hypertension, preeclampsia and diabetes. Scand. J. Clin. Lab. Invest. 52, 387–392. 10.3109/00365519209088374 1514017

[B269] Kulandavelu S. Qu D. Adamson S. L. (2006). Cardiovascular function in mice during normal pregnancy and in the absence of endothelial NO synthase. Hypertension 47, 1175–1182. 10.1161/01.HYP.0000218440.71846.db 16636199

[B270] Kulkarni R. N. Wang Z. L. Wang R. M. Hurley J. D. Smith D. M. Ghatei M. A.. (1997). Leptin rapidly suppresses insulin release from insulinoma cells, rat and human islets and, *in vivo*, in mice. J. Clin. Invest. 100, 2729–2736. 10.1172/JCI119818 9389736 PMC508476

[B271] Kumar P. Kamat A. Mendelson C. R. (2009). Estrogen receptor alpha (ERalpha) mediates stimulatory effects of estrogen on aromatase (CYP19) gene expression in human placenta. Mol. Endocrinol. 23, 784–793. 10.1210/me.2008-0371 19299445 PMC2691685

[B272] Ladyman S. R. Augustine R. A. Grattan D. R. (2010). Hormone interactions regulating energy balance during pregnancy. J. Neuroendocrinol. 22, 805–817. 10.1111/j.1365-2826.2010.02017.x 20456605

[B273] Lain K. Y. Catalano P. M. (2007). Metabolic changes in pregnancy. Clin. Obstet. Gynecol. 50, 938–948. 10.1097/GRF.0b013e31815a5494 17982337

[B274] Lanoix D. Lacasse A. A. Reiter R. J. Vaillancourt C. (2013). Melatonin: the watchdog of villous trophoblast homeostasis against hypoxia/reoxygenation-induced oxidative stress and apoptosis. Mol. Cell. Endocrinol. 381, 35–45. 10.1016/j.mce.2013.07.010 23886990

[B275] Lapensee C. R. Horseman N. D. Tso P. Brandebourg T. D. Hugo E. R. Ben-Jonathan N. (2006). The prolactin-deficient mouse has an unaltered metabolic phenotype. Endocrinology 147, 4638–4645. 10.1210/en.2006-0487 16809445

[B276] Lapierre H. Pelletier G. Petitclerc D. Dubreuil P. Morisset J. Gaudreau P.. (1988). Effect of human growth hormone-releasing factor (1-29)NH2 on growth hormone release and milk production in dairy cows. J. Dairy Sci. 71, 92–98. 10.3168/jds.S0022-0302(88)79529-6 2897385

[B277] Laporta J. Keil K. P. Vezina C. M. Hernandez L. L. (2014a). Peripheral serotonin regulates maternal calcium trafficking in mammary epithelial cells during lactation in mice. PLoS ONE 9:e110190. 10.1371/journal.pone.0110190 25299122 PMC4192539

[B278] Laporta J. Keil K. P. Weaver S. R. Cronick C. M. Prichard A. P. Crenshaw T. D.. (2014b). Serotonin regulates calcium homeostasis in lactation by epigenetic activation of hedgehog signaling. Mol. Endocrinol. 28, 1866–1874. 10.1210/me.2014-1204 25192038 PMC4213360

[B279] Laporta J. Moore S. A. Weaver S. R. Cronick C. M. Olsen M. Prichard A. P.. (2015). Increasing serotonin concentrations alter calcium and energy metabolism in dairy cows. J. Endocrinol. 226, 43–55. 10.1530/JOE-14-0693 26099356

[B280] Laporta J. Peters T. L. Merriman K. E. Vezina C. M. Hernandez L. L. (2013a). Serotonin (5-HT) affects expression of liver metabolic enzymes and mammary gland glucose transporters during the transition from pregnancy to lactation. PLoS ONE 8:e57847. 10.1371/journal.pone.0057847 23469086 PMC3585179

[B281] Laporta J. Peters T. L. Weaver S. R. Merriman K. E. Hernandez L. L. (2013b). Feeding 5-hydroxy-l-tryptophan during the transition from pregnancy to lactation increases calcium mobilization from bone in rats. Domest. Anim. Endocrinol. 44, 176–184. 10.1016/j.domaniend.2013.01.005 23433710

[B282] Laurent L. Deroy K. St-Pierre J. Côté F. Sanderson J. T. Vaillancourt C. (2017). Human placenta expresses both peripheral and neuronal isoform of tryptophan hydroxylase. Biochimie 140, 159–165. 10.1016/j.biochi.2017.07.008 28751217

[B283] Lee C. L. Chiu P. C. Hautala L. Salo T. Yeung W. S. Stenman U. H.. (2013). Human chorionic gonadotropin and its free beta-subunit stimulate trophoblast invasion independent of LH/hCG receptor. Mol. Cell. Endocrinol. 375, 43–52. 10.1016/j.mce.2013.05.009 23684886

[B284] Lee H. J. Caldwell H. K. Macbeth A. H. Tolu S. G. Young W. S. III. (2008). A conditional knockout mouse line of the oxytocin receptor. Endocrinology 149, 3256–3263. 10.1210/en.2007-1710 18356275 PMC2453083

[B285] Lee H. J. Gallego-Ortega D. Ledger A. Schramek D. Joshi P. Szwarc M. M.. (2013). Progesterone drives mammary secretory differentiation via RankL-mediated induction of Elf5 in luminal progenitor cells. Development 140, 1397–1401. 10.1242/dev.088948 23462470

[B286] Lee O. H. Bae S. K. Bae M. H. Lee Y. M. Moon E. J. Cha H. J.. (2000). Identification of angiogenic properties of insulin-like growth factor II in *in vitro* angiogenesis models. Br. J. Cancer 82, 385–391. 10.1054/bjoc.1999.0931 10646893 PMC2363289

[B287] Lee S. J. Talamantes F. Wilder E. Linzer D. I. Nathans D. (1988). Trophoblastic giant cells of the mouse placenta as the site of proliferin synthesis. Endocrinology 122, 1761–1768. 10.1210/endo-122-5-1761 3359962

[B288] Lee W. S. Lu Y. C. Kuo C. T. Chen C. T. Tang P. H. (2017). Effects of female sex hormones on folic acid-induced anti-angiogenesis. Acta Physiol. (Oxf). 222:e13001. 10.1111/apha.13001 29178430

[B289] Lefebvre D. L. Giaid A. Zingg H. H. (1992). Expression of the oxytocin gene in rat placenta. Endocrinology 130, 1185–1192. 1537285 10.1210/endo.130.3.1537285

[B290] Lekgabe E. D. Royce S. G. Hewitson T. D. Tang M. L. Zhao C. Moore X. L.. (2006). The effects of relaxin and estrogen deficiency on collagen deposition and hypertrophy of nonreproductive organs. Endocrinology 147, 5575–5583. 10.1210/en.2006-0533 16935837

[B291] Le T. N. Elsea S. H. Romero R. Chaiworapongsa T. Francis G. L. (2013). Prolactin receptor gene polymorphisms are associated with gestational diabetes. Genet. Test. Mol. Biomarkers 17, 567–571. 10.1089/gtmb.2013.0009 23651351 PMC3700434

[B292] Leturque A. Burnol A. F. Ferré P. Girard J. (1984). Pregnancy-induced insulin resistance in the rat: assessment by glucose clamp technique. Am. J. Physiol. 246, E25–31. 10.1152/ajpendo.1984.246.1.E25 6364830

[B293] Levine A. Zagoory-Sharon O. Feldman R. Weller A. (2007). Oxytocin during pregnancy and early postpartum: individual patterns and maternal-fetal attachment. Peptides 28, 1162–1169. 10.1016/j.peptides.2007.04.016 17513013

[B294] Lévy F. (2016). Neuroendocrine control of maternal behavior in non-human and human mammals. Ann. Endocrinol. (Paris). 77, 114–125. 10.1016/j.ando.2016.04.002 27130073

[B295] Liao S. Vickers M. H. Evans A. Stanley J. L. Baker P. N. Perry J. K. (2016a). Comparison of pulsatile vs. continuous administration of human placental growth hormone in female C57BL/6J mice. Endocrine 54, 169–181. 10.1007/s12020-016-1060-0 27515803

[B296] Liao S. Vickers M. H. Stanley J. L. Ponnampalam A. P. Baker P. N. Perry J. K. (2016b). The placental variant of human growth hormone reduces maternal insulin sensitivity in a dose-dependent manner in C57BL/6J Mice. Endocrinology 157, 1175–1186. 10.1210/en.2015-1718 26671184

[B297] Li J. Umar S. Amjedi M. Iorga A. Sharma S. Nadadur R. D.. (2012). New frontiers in heart hypertrophy during pregnancy. Am. J. Cardiovasc. Dis. 2, 192–207. 22937489 PMC3427979

[B298] Lim R. Acharya R. Delpachitra P. Hobson S. Sobey C. G. Drummond G. R.. (2015). Activin and NADPH-oxidase in preeclampsia: insights from *in vitro* and murine studies. Am. J. Obstet. Gynecol. 212, 86 e81–86 e12. 10.1016/j.ajog.2014.07.021 25046804

[B299] Lin B. Zhu S. Shao B. (1996). Changes of plasma levels of monoamines in normal pregnancy and pregnancy-induced hypertension women and their significance. Zhonghua Fu Chan Ke Za Zhi 31, 670–672. 9387528

[B300] Linzer D. I. Fisher S. J. (1999). The placenta and the prolactin family of hormones: regulation of the physiology of pregnancy. Mol. Endocrinol. 13, 837–840. 10.1210/mend.13.6.0286 10379883

[B301] Lissauer D. Eldershaw S. A. Inman C. F. Coomarasamy A. Moss P. A. Kilby M. D. (2015). Progesterone promotes maternal-fetal tolerance by reducing human maternal T-cell polyfunctionality and inducing a specific cytokine profile. Eur. J. Immunol. 45, 2858–2872. 10.1002/eji.201445404 26249148 PMC4833190

[B302] Liu D. Wei N. Man H. Y. Lu Y. Zhu L. Q. Wang J. Z. (2015). The MT2 receptor stimulates axonogenesis and enhances synaptic transmission by activating Akt signaling. Cell Death Differ. 22, 583–596. 10.1038/cdd.2014.195 25501601 PMC4356342

[B303] Liu H. Wu Y. Qiao F. Gong X. (2009). Effect of leptin on cytotrophoblast proliferation and invasion. J. Huazhong Univ. Sci. Technol. Med. Sci. 29, 631–636. 10.1007/s11596-009-0519-0 19821099

[B304] Liu L. X. Rowe G. C. Yang S. Li J. Damilano F. Chan M. C.. (2017). PDK4 inhibits cardiac pyruvate oxidation in late pregnancy. Circ. Res. 121, 1370–1378. 10.1161/CIRCRESAHA.117.311456 28928113 PMC5722682

[B305] Li Y. Klausen C. Cheng J. C. Zhu H. Leung P. C. (2014). Activin A, B, and AB increase human trophoblast cell invasion by up-regulating N-cadherin. J. Clin. Endocrinol. Metab. 99, E2216–2225. 10.1210/jc.2014-2118 25105734

[B306] Li Y. Klausen C. Zhu H. Leung P. C. (2015). Activin A Increases human trophoblast invasion by inducing SNAIL-mediated MMP2 up-regulation through ALK4. J. Clin. Endocrinol. Metab. 100, E1415–1427. 10.1210/jc.2015-2134 26305619

[B307] Lodhi R. S. Nakabayashi K. Suzuki K. Yamada A. Y. Hazama R. Ebina Y.. (2013). Relaxin has anti-apoptotic effects on human trophoblast-derived HTR-8/SV neo cells. Gynecol. Endocrinol. 29, 1051–1054. 10.3109/09513590.2013.829444 24070111

[B308] Lomauro A. Aliverti A. (2015). Respiratory physiology of pregnancy: physiology masterclass. Breathe (Sheff) 11, 297–301. 10.1183/20734735.008615 27066123 PMC4818213

[B309] Longo M. Jain V. Vedernikov Y. P. Garfield R. E. Saade G. R. (2003). Effects of recombinant human relaxin on pregnant rat uterine artery and myometrium *in vitro*. Am. J. Obstet. Gynecol. 188, 1468–1474; discussion 1474-1466. 10.1067/mob.2003.454 12824980

[B310] Lucas B. K. Ormandy C. J. Binart N. Bridges R. S. Kelly P. A. (1998). Null mutation of the prolactin receptor gene produces a defect in maternal behavior. Endocrinology 139, 4102–4107. 10.1210/endo.139.10.6243 9751488

[B311] Lu C. C. Chen J. J. Tsai S. C. Chien E. J. Chien C. H. Wang P. S. (1998). Increase of thyrotropin response to thyrotropin-releasing hormone (TRH) and TRH release in rats during pregnancy. Chin. J. Physiol. 41, 211–216. 10099868

[B312] Lumbers E. R. Pringle K. G. (2014). Roles of the circulating renin-angiotensin-aldosterone system in human pregnancy. Am. J. Physiol. Regul. Integr. Comp. Physiol. 306, R91–101. 10.1152/ajpregu.00034.2013 24089380

[B313] Lydon J. P. Demayo F. J. Funk C. R. Mani S. K. Hughes A. R. Montgomery C. A. Jr.. (1995). Mice lacking progesterone receptor exhibit pleiotropic reproductive abnormalities. Genes Dev. 9, 2266–2278. 10.1101/gad.9.18.2266 7557380

[B314] Maclennan A. H. Grant P. (1991). Human relaxin. *In vitro* response of human and pig myometrium. J. Reprod. Med. 36, 630–634. 1774723

[B315] Macrae D. J. Palavradji D. (1967). Maternal acid-base changes in pregnancy. J. Obstet. Gynaecol. Br. Commonw. 74, 11–16. 10.1111/j.1471-0528.1967.tb03925.x 6018086

[B316] Maeshima A. Shiozaki S. Tajima T. Nakazato Y. Naruse T. Kojima I. (2000). Number of glomeruli is increased in the kidney of transgenic mice expressing the truncated type II activin receptor. Biochem. Biophys. Res. Commun. 268, 445–449. 10.1006/bbrc.2000.2171 10679224

[B317] Magariños M. P. Sánchez-Margalet V. Kotler M. Calvo J. C. Varone C. L. (2007). Leptin promotes cell proliferation and survival of trophoblastic cells. Biol. Reprod. 76, 203–210. 10.1095/biolreprod.106.051391 17021346

[B318] Malik N. M. Carter N. D. Murray J. F. Scaramuzzi R. J. Wilson C. A. Stock M. J. (2001). Leptin requirement for conception, implantation, and gestation in the mouse. Endocrinology 142, 5198–5202. 10.1210/endo.142.12.8535 11713215

[B319] Malik N. M. Carter N. D. Wilson C. A. Scaramuzzi R. J. Stock M. J. Murray J. F. (2005). Leptin expression in the fetus and placenta during mouse pregnancy. Placenta 26, 47–52. 10.1016/j.placenta.2004.03.009 15664410

[B320] Mao G. Wang J. Kang Y. Tai P. Wen J. Zou Q.. (2010). Progesterone increases systemic and local uterine proportions of CD4+CD25+ Treg cells during midterm pregnancy in mice. Endocrinology 151, 5477–5488. 10.1210/en.2010-0426 20844003

[B321] Maroni E. S. De Sousa M. A. (1973). The lymphoid organs during pregnancy in the mouse. A comparison between a syngeneic and an allogeneic mating. Clin. Exp. Immunol. 13, 107–124. 4764346 PMC1553755

[B322] Marshall S. A. Leo C. H. Senadheera S. N. Girling J. E. Tare M. Parry L. J. (2016a). Relaxin deficiency attenuates pregnancy-induced adaptation of the mesenteric artery to angiotensin II in mice. Am. J. Physiol. Regul. Integr. Comp. Physiol. 310, R847–R857. 10.1152/ajpregu.00506.2015 26936785

[B323] Marshall S. A. Ng L. Unemori E. N. Girling J. E. Parry L. J. (2016b). Relaxin deficiency results in increased expression of angiogenesis- and remodelling-related genes in the uterus of early pregnant mice but does not affect endometrial angiogenesis prior to implantation. Reprod. Biol. Endocrinol. 14:11 10.1186/s12958-016-0148-y 27005936 PMC4802869

[B324] Maruo N. Nakabayashi K. Wakahashi S. Yata A. Maruo T. (2007). Effects of recombinant H2 relaxin on the expression of matrix metalloproteinases and tissue inhibitor metalloproteinase in cultured early placental extravillous trophoblasts. Endocrine 32, 303–310. 10.1007/s12020-008-9034-5 18236174

[B325] Mason G. A. Caldwell J. D. Stanley D. A. Hatley O. L. Prange A. J. Jr. Pedersen C. A. (1986). Interactive effects of intracisternal oxytocin and other centrally active substances on colonic temperatures of mice. Regul. Pept. 14, 253–260. 10.1016/0167-0115(86)90008-X 2941825

[B326] Masuzaki H. Ogawa Y. Sagawa N. Hosoda K. Matsumoto T. Mise H.. (1997). Nonadipose tissue production of leptin: leptin as a novel placenta-derived hormone in humans. Nat. Med. 3, 1029–1033. 10.1038/nm0997-1029 9288733

[B327] Matjila M. Millar R. Van Der Spuy Z. Katz A. (2016). Elevated placental expression at the maternal-fetal interface but diminished maternal circulatory kisspeptin in preeclamptic pregnancies. Pregnancy Hypertens. 6, 79–87. 10.1016/j.preghy.2015.11.001 26955777

[B328] Mayerl S. Liebsch C. Visser T. J. Heuer H. (2015). Absence of TRH receptor 1 in male mice affects gastric ghrelin production. Endocrinology 156, 755–767. 10.1210/en.2014-1395 25490146

[B329] Mazella J. Tang M. Tseng L. (2004). Disparate effects of relaxin and TGFbeta1: relaxin increases, but TGFbeta1 inhibits, the relaxin receptor and the production of IGFBP-1 in human endometrial stromal/decidual cells. Hum. Reprod. 19, 1513–1518. 10.1093/humrep/deh274 15155604

[B330] Mcilvride S. Mushtaq A. Papacleovoulou G. Hurling C. Steel J. Jansen E.. (2017). A progesterone-brown fat axis is involved in regulating fetal growth. Sci. Rep. 7:10671. 10.1038/s41598-017-10979-7 28878263 PMC5587669

[B331] Mead E. J. Maguire J. J. Kuc R. E. Davenport A. P. (2007). Kisspeptins: a multifunctional peptide system with a role in reproduction, cancer and the cardiovascular system. Br. J. Pharmacol. 151, 1143–1153. 10.1038/sj.bjp.0707295 17519946 PMC2189831

[B332] Meziani F. Van Overloop B. Schneider F. Gairard A. (2005). Parathyroid hormone-related protein-induced relaxation of rat uterine arteries: influence of the endothelium during gestation. J. Soc. Gynecol. Investig. 12, 14–19. 10.1016/j.jsgi.2004.07.005 15629665

[B333] Miedlar J. A. Rinaman L. Vollmer R. R. Amico J. A. (2007). Oxytocin gene deletion mice overconsume palatable sucrose solution but not palatable lipid emulsions. Am. J. Physiol. Regul. Integr. Comp. Physiol. 293, R1063–R1068. 10.1152/ajpregu.00228.2007 17596329

[B334] Mikaelsson M. A. Constância M. Dent C. L. Wilkinson L. S. Humby T. (2013). Placental programming of anxiety in adulthood revealed by Igf2-null models. Nat. Commun. 4:2311. 10.1038/ncomms3311 23921428

[B335] Milczarek R. Hallmann A. Sokołowska E. Kaletha K. Klimek J. (2010). Melatonin enhances antioxidant action of alpha-tocopherol and ascorbate against NADPH- and iron-dependent lipid peroxidation in human placental mitochondria. J. Pineal Res. 49, 149–155. 10.1111/j.1600-079X.2010.00779.x 20524970

[B336] Miller S. L. Yawno T. Alers N. O. Castillo-Melendez M. Supramaniam V. G. Vanzyl N.. (2014). Antenatal antioxidant treatment with melatonin to decrease newborn neurodevelopmental deficits and brain injury caused by fetal growth restriction. J. Pineal Res. 56, 283–294. 10.1111/jpi.12121 24456220

[B337] Mirabito Colafella K. M. Samuel C. S. Denton K. M. (2017). Relaxin contributes to the regulation of arterial pressure in adult female mice. Clin. Sci. 131, 2795–2805. 10.1042/CS20171225 29101299

[B338] Miranda A. Sousa N. (2018). Maternal hormonal milieu influence on fetal brain development. Brain Behav. 8:e00920. 10.1002/brb3.920 29484271 PMC5822586

[B339] Mitchell J. A. Hammer R. E. Goldman H. (1983). Serotonin-induced disruption of implantation in the rat: II. Suppression of decidualization. Biol. Reprod 29, 151–156. 10.1095/biolreprod29.1.151 6615961

[B340] Modi H. Jacovetti C. Tarussio D. Metref S. Madsen O. D. Zhang F. P.. (2015). Autocrine action of IGF2 regulates adult beta-cell mass and function. Diabetes 64, 4148–4157. 10.2337/db14-1735 26384384

[B341] Mohammadi-Sartang M. Ghorbani M. Mazloom Z. (2017). Effects of melatonin supplementation on blood lipid concentrations: a systematic review and meta-analysis of randomized controlled trials. Clin. Nutr. [Epub ahead of print]. 10.1016/j.clnu.2017.11.003 29191493

[B342] Mor G. Cardenas I. (2010). The immune system in pregnancy: a unique complexity. Am J Reprod Immunol 63, 425–433. 10.1111/j.1600-0897.2010.00836.x 20367629 PMC3025805

[B343] Morrissy S. Xu B. Aguilar D. Zhang J. Chen Q. M. (2010). Inhibition of apoptosis by progesterone in cardiomyocytes. Aging Cell 9, 799–809. 10.1111/j.1474-9726.2010.00619.x 20726854 PMC4133411

[B344] Mounzih K. Qiu J. Ewart-Toland A. Chehab F. F. (1998). Leptin is not necessary for gestation and parturition but regulates maternal nutrition via a leptin resistance state. Endocrinology 139, 5259–5262. 10.1210/endo.139.12.6523 9832467

[B345] Moya F. Mena P. Heusser F. Foradori A. Paiva E. Yazigi R.. (1986). Response of the maternal, fetal, and neonatal pituitary-thyroid axis to thyrotropin-releasing hormone. Pediatr. Res. 20, 982–986. 10.1203/00006450-198610000-00018 3095783

[B346] Mühlbauer E. Albrecht E. Bazwinsky-Wutschke I. Peschke E. (2012). Melatonin influences insulin secretion primarily via MT(1) receptors in rat insulinoma cells (INS-1) and mouse pancreatic islets. J. Pineal Res. 52, 446–459. 10.1111/j.1600-079X.2012.00959.x 22288848

[B347] Mühlbauer E. Gross E. Labucay K. Wolgast S. Peschke E. (2009). Loss of melatonin signalling and its impact on circadian rhythms in mouse organs regulating blood glucose. Eur. J. Pharmacol. 606, 61–71. 10.1016/j.ejphar.2009.01.029 19374844

[B348] Müller H. Liu B. Croy B. A. Head J. R. Hunt J. S. Dai G.. (1999). Uterine natural killer cells are targets for a trophoblast cell-specific cytokine, prolactin-like protein A. Endocrinology 140, 2711–2720. 10.1210/endo.140.6.6828 10342862

[B349] Munnell E. W. Taylor H. C. (1947). Liver Blood Flow in Pregnancy-Hepatic Vein Catheterization. J. Clin. Invest. 26, 952–956. 10.1172/JCI101890 16695499 PMC439394

[B350] Musial B. Fernandez-Twinn D. S. Vaughan O. R. Ozanne S. E. Voshol P. Sferruzzi-Perri A. N.. (2016). Proximity to Delivery Alters Insulin Sensitivity and Glucose Metabolism in Pregnant Mice. Diabetes 65, 851–860. 10.2337/db15-1531 26740602 PMC4876930

[B351] Musial B. Vaughan O. R. Fernandez-Twinn D. S. Voshol P. Ozanne S. E. Fowden A. L.. (2017). A Western-style obesogenic diet alters maternal metabolic physiology with consequences for fetal nutrient acquisition in mice. J. Physiol. (Lond). 595, 4875–4892. 10.1113/JP273684 28382681 PMC5509867

[B352] Muttukrishna S. Child T. J. Groome N. P. Ledger W. L. (1997). Source of circulating levels of inhibin A, pro alpha C-containing inhibins and activin A in early pregnancy. Hum. Reprod. 12, 1089–1093. 10.1093/humrep/12.5.1089 9194671

[B353] Nakamura Y. Tamura H. Kashida S. Takayama H. Yamagata Y. Karube A.. (2001). Changes of serum melatonin level and its relationship to feto-placental unit during pregnancy. J. Pineal Res. 30, 29–33. 10.1034/j.1600-079X.2001.300104.x 11168904

[B354] Nanetti L. Raffaelli F. Giulietti A. Sforza G. Raffaele Giannubilo S. Ciavattini A.. (2015). Oxytocin, its antagonist Atosiban, and preterm labor: a role for placental nitric oxide. J. Matern. Fetal Neonatal Med. 28, 611–616. 10.3109/14767058.2014.927859 24920283

[B355] Nathanielsz P. W. Jenkins S. L. Tame J. D. Winter J. A. Guller S. Giussani D. A. (1998). Local paracrine effects of estradiol are central to parturition in the rhesus monkey. Nat. Med. 4, 456–459. 10.1038/nm0498-456 9546793

[B356] Neville M. C. Mcfadden T. B. Forsyth I. (2002). Hormonal regulation of mammary differentiation and milk secretion. J. Mammary Gland Biol. Neoplasia 7, 49–66. 10.1023/A:1015770423167 12160086

[B357] Nielsen J. H. (1982). Effects of growth hormone, prolactin, and placental lactogen on insulin content and release, and deoxyribonucleic acid synthesis in cultured pancreatic islets. Endocrinology 110, 600–606. 10.1210/endo-110-2-600 6276141

[B358] Nien J. K. Mazaki-Tovi S. Romero R. Erez O. Kusanovic J. P. Gotsch F.. (2007). Plasma adiponectin concentrations in non-pregnant, normal and overweight pregnant women. J. Perinat. Med. 35, 522–531. 10.1515/JPM.2007.123 17919116 PMC2410085

[B359] Nir I. Hirschmann N. (1980). Melatonin-induced changes in blood and pituitary luteinizing hormone and prolactin levels during the perinatal period in rat dams. J Neural Transm 49, 219–228. 10.1007/BF01252127 6109752

[B360] Nishimori K. Young L. J. Guo Q. Wang Z. Insel T. R. Matzuk M. M. (1996). Oxytocin is required for nursing but is not essential for parturition or reproductive behavior. Proc. Natl. Acad. Sci. U.S.A. 93, 11699–11704. 10.1073/pnas.93.21.11699 8876199 PMC38121

[B361] Ni X. Luo S. Minegishi T. Peng C. (2000). Activin A in JEG-3 cells: potential role as an autocrine regulator of steroidogenesis in humans. Biol. Reprod. 62, 1224–1230. 10.1095/biolreprod62.5.1224 10775170

[B362] Norton M. T. Fortner K. A. Bizargity P. Bonney E. A. (2009). Pregnancy alters the proliferation and apoptosis of mouse splenic erythroid lineage cells and leukocytes. Biol. Reprod. 81, 457–464. 10.1095/biolreprod.109.076976 19369644 PMC2731983

[B363] Norwitz E. R. Caughey A. B. (2011). Progesterone supplementation and the prevention of preterm birth. Rev. Obstet. Gynecol. 4, 60–72. 22102929 PMC3218546

[B364] Obr A. E. Grimm S. L. Bishop K. A. Pike J. W. Lydon J. P. Edwards D. P. (2013). Progesterone receptor and Stat5 signaling cross talk through RANKL in mammary epithelial cells. Mol. Endocrinol. 27, 1808–1824. 10.1210/me.2013-1077 24014651 PMC3805851

[B365] O'byrne E. M. Sawyer W. K. Butler M. C. Steinetz B. G. (1976). Serum immunoreactive relaxin and softening of the uterine cervix in pregnant hamsters. Endocrinology 99, 1333–1335. 10.1210/endo-99-5-1333 991824

[B366] O'byrne E. M. Steinetz B. G. (1976). Radioimmunoassay (RIA) of relaxin in sera of various species using an antiserum to porcine relaxin. Proc. Soc. Exp. Biol. Med. 152, 272–276. 10.3181/00379727-152-39377 819937

[B367] Ogawa Y. Masuzaki H. Hosoda K. Aizawa-Abe M. Suga J. Suda M.. (1999). Increased glucose metabolism and insulin sensitivity in transgenic skinny mice overexpressing leptin. Diabetes 48, 1822–1829. 10.2337/diabetes.48.9.1822 10480614

[B368] Ogueh O. Clough A. Hancock M. Johnson M. R. (2011). A longitudinal study of the control of renal and uterine hemodynamic changes of pregnancy. Hypertens. Pregnancy 30, 243–259. 10.3109/10641955.2010.484079 21740248

[B369] Ohara-Imaizumi M. Kim H. Yoshida M. Fujiwara T. Aoyagi K. Toyofuku Y.. (2013). Serotonin regulates glucose-stimulated insulin secretion from pancreatic beta cells during pregnancy. Proc. Natl. Acad. Sci. U.S.A. 110, 19420–19425. 10.1073/pnas.1310953110 24218571 PMC3845121

[B370] Okatani Y. Wakatsuki A. Shinohara K. Taniguchi K. Fukaya T. (2001). Melatonin protects against oxidative mitochondrial damage induced in rat placenta by ischemia and reperfusion. J. Pineal Res. 31, 173–178. 10.1034/j.1600-079x.2001.310212.x 11555174

[B371] O'neal-Moffitt G. Pilli J. Kumar S. S. Olcese J. (2014). Genetic deletion of MT(1)/MT(2) melatonin receptors enhances murine cognitive and motor performance. Neuroscience 277, 506–521. 10.1016/j.neuroscience.2014.07.018 25046530

[B372] O'sullivan K. P. Marshall S. A. Cullen S. Saunders T. Hannan N. J. Senadheera S. N.. (2017). Evidence of proteinuria, but no other characteristics of pre-eclampsia, in relaxin-deficient mice. Reprod. Fertil. Dev. 29, 1477–1485. 10.1071/RD16056 27489037

[B373] Owino S. Contreras-Alcantara S. Baba K. Tosini G. (2016). Melatonin signaling controls the daily rhythm in blood glucose levels independent of peripheral clocks. PLoS ONE 11:e0148214. 10.1371/journal.pone.0148214 26824606 PMC4732609

[B374] Palejwala S. Stein D. E. Weiss G. Monia B. P. Tortoriello D. Goldsmith L. T. (2001). Relaxin positively regulates matrix metalloproteinase expression in human lower uterine segment fibroblasts using a tyrosine kinase signaling pathway. Endocrinology 142, 3405–3413. 10.1210/endo.142.8.8295 11459784

[B375] Paller M. S. Gregorini G. Ferris T. F. (1989). Pressor responsiveness in pseudopregnant and pregnant rats: role of maternal factors. Am. J. Physiol. 257, R866–R871. 10.1152/ajpregu.1989.257.4.R866 2802003

[B376] Pang W. W. Hartmann P. E. (2007). Initiation of human lactation: secretory differentiation and secretory activation. J. Mammary Gland Biol. Neoplasia 12, 211–221. 10.1007/s10911-007-9054-4 18027076

[B377] Pecins-Thompson M. Keller-Wood M. (1997). Effects of progesterone on blood pressure, plasma volume, and responses to hypotension. Am. J. Physiol. 272, R377–385. 10.1152/ajpregu.1997.272.1.R377 9039032

[B378] Pedersen C. A. Vadlamudi S. V. Boccia M. L. Amico J. A. (2006). Maternal behavior deficits in nulliparous oxytocin knockout mice. Genes Brain Behav. 5, 274–281. 10.1111/j.1601-183X.2005.00162.x 16594980

[B379] Pelletier G. Petitclerc D. Lapierre H. Bernier-Cardou M. Morisset J. Gaudreau P.. (1987). Injection of synthetic human growth hormone-releasing factors in dairy cows. 1. Effect on feed intake and milk yield and composition. J. Dairy Sci. 70, 2511–2517. 10.3168/jds.S0022-0302(87)80319-3 2896206

[B380] Pelleymounter M. A. Cullen M. J. Baker M. B. Hecht R. Winters D. Boone T.. (1995). Effects of the obese gene product on body weight regulation in ob/ob mice. Science 269, 540–543. 10.1126/science.7624776 7624776

[B381] Peng J. Fullerton P. T. Jr. Monsivais D. Clementi C. Su G. H. Matzuk M. M. (2015). Uterine activin-like kinase 4 regulates trophoblast development during mouse placentation. Mol. Endocrinol. 29, 1684–1693. 10.1210/me.2015-1048 26484579 PMC4664232

[B382] Petersson M. Alster P. Lundeberg T. Uvnäs-Moberg K. (1996). Oxytocin causes a long-term decrease of blood pressure in female and male rats. Physiol. Behav. 60, 1311–1315. 10.1016/S0031-9384(96)00261-2 8916187

[B383] Petry C. J. Evans M. L. Wingate D. L. Ong K. K. Reik W. Constância M.. (2010). Raised late pregnancy glucose concentrations in mice carrying pups with targeted disruption of H19delta13. Diabetes 59, 282–286. 10.2337/db09-0757 19794064 PMC2797934

[B384] Petry C. J. Ong K. K. Dunger D. B. (2007). Does the fetal genotype affect maternal physiology during pregnancy? Trends Mol. Med. 13, 414–421. 10.1016/j.molmed.2007.07.007 17900986

[B385] Pieper P. G. (2015). Use of medication for cardiovascular disease during pregnancy. Nat. Rev. Cardiol. 12, 718–729. 10.1038/nrcardio.2015.172 26585398

[B386] Pitera A. E. Smith G. C. Wentworth R. A. Nathanielsz P. W. (1998). Parathyroid hormone-related peptide (1 to 34) inhibits *in vitro* oxytocin-stimulated activity of pregnant baboon myometrium. Am. J. Obstet. Gynecol. 179, 492–496. 10.1016/S0002-9378(98)70385-0 9731859

[B387] Plaut K. Maple R. Ginsburg E. Vonderhaar B. (1999). Progesterone stimulates DNA synthesis and lobulo-alveolar development in mammary glands in ovariectomized mice. J. Cell Physiol. 180, 298–304. 10.1002/(SICI)1097-4652(199908)180:2<298::AID-JCP17>3.0.CO;2-V 10395299

[B388] Porter S. E. Sorenson R. L. Dann P. Garcia-Ocana A. Stewart A. F. Vasavada R. C. (1998). Progressive pancreatic islet hyperplasia in the islet-targeted, parathyroid hormone-related protein-overexpressing mouse. Endocrinology 139, 3743–3751. 10.1210/endo.139.9.6212 9724026

[B389] Poulson E. Botros M. Robson J. M. (1960). Effect of 5-hydroxytryptamine and iproniazid on pregnancy. Science 131, 1101–1102. 10.1126/science.131.3407.1101 14434518

[B390] Prast J. Saleh L. Husslein H. Sonderegger S. Helmer H. Knöfler M. (2008). Human chorionic gonadotropin stimulates trophoblast invasion through extracellularly regulated kinase and AKT signaling. Endocrinology 149, 979–987. 10.1210/en.2007-1282 18063683 PMC2974217

[B391] Prezotto L. D. Lemley C. O. Camacho L. E. Doscher F. E. Meyer A. M. Caton J. S.. (2014). Effects of nutrient restriction and melatonin supplementation on maternal and foetal hepatic and small intestinal energy utilization. J. Anim. Physiol. Anim. Nutr. (Berl). 98, 797–807. 10.1111/jpn.12142 25180375

[B392] Prigent-Tessier A. Pageaux J. F. Fayard J. M. Lagarde M. Laugier C. Cohen H. (1996). Prolactin up-regulates prostaglandin E2 production through increased expression of pancreatic-type phospholipase A2 (type I) and prostaglandin G/H synthase 2 in uterine cells. Mol. Cell. Endocrinol. 122, 101–108. 10.1016/0303-7207(96)03888-9 8898352

[B393] Qi X. Gong B. Yu J. Shen L. Jin W. Wu Z.. (2017). Decreased cord blood estradiol levels in related to mothers with gestational diabetes. Medicine (Baltimore). 96:e6962. 10.1097/MD.0000000000006962 28538390 PMC5457870

[B394] Quagliarello J. Szlachter N. Steinetz B. G. Goldsmith L. T. Weiss G. (1979). Serial relaxin concentrations in human pregnancy. Am. J. Obstet. Gynecol. 135, 43–44. 474660

[B395] Qu J. Thomas K. (1993). Regulation of inhibin secretion in human placental cell culture by epidermal growth factor, transforming growth factors, and activin. J. Clin. Endocrinol. Metab. 77, 925–931. 8408467 10.1210/jcem.77.4.8408467

[B396] Rabeler R. Mittag J. Geffers L. Rüther U. Leitges M. Parlow A. F.. (2004). Generation of thyrotropin-releasing hormone receptor 1-deficient mice as an animal model of central hypothyroidism. Mol. Endocrinol. 18, 1450–1460. 10.1210/me.2004-0017 14988432

[B397] Racicot K. Kwon J. Y. Aldo P. Silasi M. Mor G. (2014). Understanding the complexity of the immune system during pregnancy. Am J Reprod Immunol 72, 107–116. 10.1111/aji.12289 24995526 PMC6800182

[B398] Randle P. J. (1998). Regulatory interactions between lipids and carbohydrates: the glucose fatty acid cycle after 35 years. Diabetes Metab. Rev. 14, 263–283. 10.1002/(sici)1099-0895(199812)14:4<263::aid-dmr233>3.0.co;2-c 10095997

[B399] Rawn S. M. Huang C. Hughes M. Shaykhutdinov R. Vogel H. J. Cross J. C. (2015). Pregnancy hyperglycemia in prolactin receptor mutant, but not prolactin mutant, mice and feeding-responsive regulation of placental lactogen genes implies placental control of maternal glucose homeostasis. Biol. Reprod. 93:75 10.1095/biolreprod.115.132431 26269505 PMC4710193

[B400] Renegar R. H. Owens C. R. III. (2002). Measurement of plasma and tissue relaxin concentrations in the pregnant hamster and fetus using a homologous radioimmunoassay. Biol. Reprod. 67, 500–505. 10.1095/biolreprod67.2.500 12135888

[B401] Rezaei R. Wu Z. Hou Y. Bazer F. W. Wu G. (2016). Amino acids and mammary gland development: nutritional implications for milk production and neonatal growth. J. Anim. Sci. Biotechnol. 7:20. 10.1186/s40104-016-0078-8 27042295 PMC4818943

[B402] Ribas V. Drew B. G. Le J. A. Soleymani T. Daraei P. Sitz D.. (2011). Myeloid-specific estrogen receptor alpha deficiency impairs metabolic homeostasis and accelerates atherosclerotic lesion development. Proc. Natl. Acad. Sci. U.S.A. 108, 16457–16462. 10.1073/pnas.1104533108 21900603 PMC3182726

[B403] Ribeiro A. C. Musatov S. Shteyler A. Simanduyev S. Arrieta-Cruz I. Ogawa S.. (2012). siRNA silencing of estrogen receptor-alpha expression specifically in medial preoptic area neurons abolishes maternal care in female mice. Proc. Natl. Acad. Sci. U.S.A. 109, 16324–16329. 10.1073/pnas.1214094109 22988120 PMC3479618

[B404] Rieck S. Kaestner K. H. (2010). Expansion of beta-cell mass in response to pregnancy. Trends Endocrinol. Metab. 21, 151–158. 10.1016/j.tem.2009.11.001 20015659 PMC3627215

[B405] Robinson D. P. Klein S. L. (2012). Pregnancy and pregnancy-associated hormones alter immune responses and disease pathogenesis. Horm. Behav. 62, 263–271. 10.1016/j.yhbeh.2012.02.023 22406114 PMC3376705

[B406] Robson J. M. Sullivan F. M. (1966). Analysis of actions of 5-hydroxytryptamine in pregnancy. J. Physiol. (Lond). 184, 717–732. 10.1113/jphysiol.1966.sp007943 5963741 PMC1357611

[B407] Rodger M. Sheppard D. Gándara E. Tinmouth A. (2015). Haematological problems in obstetrics. Best Pract. Res. Clin. Obstet. Gynaecol. 29, 671–684. 10.1016/j.bpobgyn.2015.02.004 25819750

[B408] Romero M. Ortega A. Izquierdo A. López-Luna P. Bosch R. J. (2010). Parathyroid hormone-related protein induces hypertrophy in podocytes via TGF-beta(1) and p27(Kip1): implications for diabetic nephropathy. Nephrol. Dial. Transplant 25, 2447–2457. 10.1093/ndt/gfq104 20200004

[B409] Roos A. Robertson F. Lochner C. Vythilingum B. Stein D. J. (2011). Altered prefrontal cortical function during processing of fear-relevant stimuli in pregnancy. Behav. Brain Res. 222, 200–205. 10.1016/j.bbr.2011.03.055 21458497

[B410] Roti E. Gnudi A. Braverman L. E. Robuschi G. Emanuele R. Bandini P.. (1981). Human cord blood concentrations of thyrotropin, thyroglobulin, and iodothyronines after maternal administration of thyrotropin-releasing hormone. J. Clin. Endocrinol. Metab. 53, 813–817. 10.1210/jcem-53-4-813 6793611

[B411] Rozenblit-Susan S. Chapnik N. Froy O. (2017). Serotonin prevents differentiation into brown adipocytes and induces transdifferentiation into white adipocytes. Int. J. Obes (Lond). 42, 704–710. 10.1038/ijo.2017.261 29081505

[B412] Ryan E. A. O'sullivan M. J. Skyler J. S. (1985). Insulin action during pregnancy. Studies with the euglycemic clamp technique. Diabetes 34, 380–389. 10.2337/diab.34.4.380 3882502

[B413] Rybakowski C. Niemax K. Goepel E. Schröder H. J. (2000). The effect of oxytocin, prostaglandin E2 and acetylsalicylic acid on flow distribution and on the transfer of alanine, glucose and water in isolated perfused guinea pig placentae. Placenta 21, 126–131. 10.1053/plac.1999.0459 10692261

[B414] Rygaard K. Revol A. Esquivel-Escobedo D. Beck B. L. Barrera-Saldana H. A. (1998). Absence of human placental lactogen and placental growth hormone (HGH-V) during pregnancy: PCR analysis of the deletion. Hum. Genet. 102, 87–92. 10.1007/s004390050658 9490304

[B415] Sagawa N. Yura S. Itoh H. Mise H. Kakui K. Korita D.. (2002). Role of leptin in pregnancy–a review. Placenta 23(Suppl. A), S80–S86. 10.1053/plac.2002.0814 11978063

[B416] Sairenji T. J. Ikezawa J. Kaneko R. Masuda S. Uchida K. Takanashi Y.. (2017). Maternal prolactin during late pregnancy is important in generating nurturing behavior in the offspring. Proc. Natl. Acad. Sci. U.S.A. 114, 13042–13047. 10.1073/pnas.1621196114 29158391 PMC5724246

[B417] Saito S. Nakashima A. Shima T. Ito M. (2010). Th1/Th2/Th17 and regulatory T-cell paradigm in pregnancy. Am. J. Reprod Immunol. 63, 601–610. 10.1111/j.1600-0897.2010.00852.x 20455873

[B418] Salles J. P. (2016). Bone metabolism during pregnancy. Ann. Endocrinol. (Paris). 77, 163–168. 10.1016/j.ando.2016.04.004 27157104

[B419] Samuel C. S. Zhao C. Bathgate R. A. Bond C. P. Burton M. D. Parry L. J.. (2003). Relaxin deficiency in mice is associated with an age-related progression of pulmonary fibrosis. FASEB J. 17, 121–123. 10.1096/fj.02-0449fje 12424226

[B420] Sandoval-Guzmán T. Gongrich C. Moliner A. Guo T. Wu H. Broberger C.. (2012). Neuroendocrine control of female reproductive function by the activin receptor ALK7. FASEB J. 26, 4966–4976. 10.1096/fj.11-199059 22954591

[B421] Sasaki K. Matsumura G. Ito T. (1981). Effects of pregnancy on erythropoiesis in the splenic red pulp of the mouse: a quantitative electron microscopic study. Arch. Histol. Jpn. 44, 429–438. 10.1679/aohc1950.44.429 7325783

[B422] Sasaki Y. Morimoto T. Saito H. Suzuki M. Ichizuka K. Yanaihara T. (2000). The role of parathyroid hormone-related protein in intra-tracheal fluid. Endocr. J. 47, 169–175. 10.1507/endocrj.47.169 10943741

[B423] Scarpace P. J. Matheny M. Pollock B. H. Tümer N. (1997). Leptin increases uncoupling protein expression and energy expenditure. Am. J. Physiol. 273, E226–230. 10.1152/ajpendo.1997.273.1.E226 9252501

[B424] Schanton M. Maymó J. L. Pérez-Pérez A. Sánchez-Margalet V. Varone C. L. (2018). Involvement of leptin in the molecular physiology of the placenta. Reproduction 155, R1–R12. 10.1530/REP-17-0512 29018059

[B425] Schipani E. Lanske B. Hunzelman J. Luz A. Kovacs C. S. Lee K.. (1997). Targeted expression of constitutively active receptors for parathyroid hormone and parathyroid hormone-related peptide delays endochondral bone formation and rescues mice that lack parathyroid hormone-related peptide. Proc. Natl. Acad. Sci. U.S.A. 94, 13689–13694. 10.1073/pnas.94.25.13689 9391087 PMC28367

[B426] Schulz L. C. Widmaier E. P. (2004). The effect of leptin on mouse trophoblast cell invasion. Biol. Reprod. 71, 1963–1967. 10.1095/biolreprod.104.032722 15306556

[B427] Schumacher A. Heinze K. Witte J. Poloski E. Linzke N. Woidacki K.. (2013). Human chorionic gonadotropin as a central regulator of pregnancy immune tolerance. J. Immunol. 190, 2650–2658. 10.4049/jimmunol.1202698 23396945

[B428] Sclafani A. Rinaman L. Vollmer R. R. Amico J. A. (2007). Oxytocin knockout mice demonstrate enhanced intake of sweet and nonsweet carbohydrate solutions. Am. J. Physiol. Regul. Integr. Comp. Physiol. 292, R1828–1833. 10.1152/ajpregu.00826.2006 17272659 PMC2360481

[B429] Scott P. R. Sargison N. D. Macrae A. I. Gough M. R. (2009). Melatonin treatment prior to the normal breeding season increases fetal number in United Kingdom sheep flocks. Vet. J. 182, 198–202. 10.1016/j.tvjl.2008.07.010 18783969

[B430] Seki K. Uesato T. Tabei T. Kato K. (1985). The secretory patterns of relaxin and human chorionic gonadotropin in human pregnancy. Endocrinol. Jpn. 32, 741–744. 10.1507/endocrj1954.32.741 4092674

[B431] Seufert J. Kieffer T. J. Leech C. A. Holz G. G. Moritz W. Ricordi C.. (1999). Leptin suppression of insulin secretion and gene expression in human pancreatic islets: implications for the development of adipogenic diabetes mellitus. J. Clin. Endocrinol. Metab. 84, 670–676. 10.1210/jc.84.2.670 10022436 PMC2927866

[B432] Sferruzzi-Perri A. N. Owens J. A. Pringle K. G. Robinson J. S. Roberts C. T. (2006). Maternal insulin-like growth factors-I and -II act via different pathways to promote fetal growth. Endocrinology 147, 3344–3355. 10.1210/en.2005-1328 16556757

[B433] Sferruzzi-Perri A. N. Owens J. A. Standen P. Taylor R. L. Heinemann G. K. Robinson J. S.. (2007). Early treatment of the pregnant guinea pig with IGFs promotes placental transport and nutrient partitioning near term. Am. J. Physiol. Endocrinol. Metab. 292, E668–676. 10.1152/ajpendo.00320.2006 17062842

[B434] Sferruzzi-Perri A. N. Vaughan O. R. Coan P. M. Suciu M. C. Darbyshire R. Constancia M.. (2011). Placental-specific Igf2 deficiency alters developmental adaptations to undernutrition in mice. Endocrinology 152, 3202–3212. 10.1210/en.2011-0240 21673101

[B435] Shahtaheri S. M. Aaron J. E. Johnson D. R. Purdie D. W. (1999). Changes in trabecular bone architecture in women during pregnancy. Br. J. Obstet. Gynaecol. 106, 432–438. 10.1111/j.1471-0528.1999.tb08296.x 10430193

[B436] Shakhmatova E. I. Osipova N. A. Natochin Y. V. (2000). Changes in osmolality and blood serum ion concentrations in pregnancy. Hum. Physiol. 26, 92–95. 10.1007/BF02760724

[B437] Sharkey J. T. Cable C. Olcese J. (2010). Melatonin sensitizes human myometrial cells to oxytocin in a protein kinase C alpha/extracellular-signal regulated kinase-dependent manner. J. Clin. Endocrinol. Metab. 95, 2902–2908. 10.1210/jc.2009-2137 20382690 PMC2902072

[B438] Sharkey J. T. Puttaramu R. Word R. A. Olcese J. (2009). Melatonin synergizes with oxytocin to enhance contractility of human myometrial smooth muscle cells. J. Clin. Endocrinol. Metab. 94, 421–427. 10.1210/jc.2008-1723 19001515 PMC2730229

[B439] Shaw L. Taggart M. Austin C. (2001). Effects of the oestrous cycle and gender on acute vasodilatory responses of isolated pressurized rat mesenteric arteries to 17 beta-oestradiol. Br. J. Pharmacol. 132, 1055–1062. 10.1038/sj.bjp.0703908 11226136 PMC1572647

[B440] Shek E. W. Brands M. W. Hall J. E. (1998). Chronic leptin infusion increases arterial pressure. Hypertension 31, 409–414. 10.1161/01.HYP.31.1.409 9453337

[B441] Shingo T. Gregg C. Enwere E. Fujikawa H. Hassam R. Geary C.. (2003). Pregnancy-stimulated neurogenesis in the adult female forebrain mediated by prolactin. Science 299, 117–120. 10.1126/science.1076647 12511652

[B442] Shi Q. J. Lei Z. M. Rao C. V. Lin J. (1993). Novel role of human chorionic gonadotropin in differentiation of human cytotrophoblasts. Endocrinology 132, 1387–1395. 10.1210/endo.132.3.7679981 7679981

[B443] Sierra-Honigmann M. R. Nath A. K. Murakami C. García-Cardeña G G. Papapetropoulos A. Sessa W. C.. (1998). Biological action of leptin as an angiogenic factor. Science 281, 1683–1686. 10.1126/science.281.5383.1683 9733517

[B444] Simmons D. G. Rawn S. Davies A. Hughes M. Cross J. C. (2008). Spatial and temporal expression of the 23 murine Prolactin/Placental Lactogen-related genes is not associated with their position in the locus. BMC Genomics 9:352 10.1186/1471-2164-9-352 18662396 PMC2527339

[B445] Simoncini T. Mannella P. Fornari L. Caruso A. Willis M. Y. Garibaldi S.. (2004). Differential signal transduction of progesterone and medroxyprogesterone acetate in human endothelial cells. Endocrinology 145, 5745–5756. 10.1210/en.2004-0510 15358673

[B446] Singh H. J. Saleh H. I. Gupalo S. Omar E. (2013). Effect of melatonin supplementation on pregnancy outcome in Wistar-Kyoto and Sprague-Dawley rats. Sheng Li Xue Bao 65, 149–157. 23598870

[B447] Slattery M. M. O'leary M. J. Morrison J. J. (2001). Effect of parathyroid hormone-related peptide on human and rat myometrial contractility *in vitro*. Am. J. Obstet. Gynecol. 184, 625–629. 10.1067/mob.2001.110695 11262463

[B448] Soares M. J. (2004). The prolactin and growth hormone families: pregnancy-specific hormones/cytokines at the maternal-fetal interface. Reprod. Biol. Endocrinol. 2:51. 10.1186/1477-7827-2-51 15236651 PMC471570

[B449] Soares M. J. Konno T. Alam S. M. (2007). The prolactin family: effectors of pregnancy-dependent adaptations. Trends Endocrinol. Metab. 18, 114–121. 10.1016/j.tem.2007.02.005 17324580

[B450] Soliman A. Lacasse A. A. Lanoix D. Sagrillo-Fagundes L. Boulard V. Vaillancourt C. (2015). Placental melatonin system is present throughout pregnancy and regulates villous trophoblast differentiation. J. Pineal Res. 59, 38–46. 10.1111/jpi.12236 25833399

[B451] Soloff M. S. Jeng Y. J. Izban M. G. Sinha M. Luxon B. A. Stamnes S. J.. (2011). Effects of progesterone treatment on expression of genes involved in uterine quiescence. Reprod. Sci. 18, 781–797. 10.1177/1933719111398150 21795739 PMC4051400

[B452] Soma-Pillay P. Nelson-Piercy C. Tolppanen H. Mebazaa A. (2016). Physiological changes in pregnancy. Cardiovasc. J. Afr. 27, 89–94. 10.5830/CVJA-2016-021 27213856 PMC4928162

[B453] Song G. J. Fiaschi-Taesch N. Bisello A. (2009). Endogenous parathyroid hormone-related protein regulates the expression of PTH type 1 receptor and proliferation of vascular smooth muscle cells. Mol. Endocrinol. 23, 1681–1690. 10.1210/me.2009-0098 19574446 PMC2754893

[B454] Song W. J. Mondal P. Wolfe A. Alonso L. C. Stamateris R. Ong B. W.. (2014). Glucagon regulates hepatic kisspeptin to impair insulin secretion. Cell Metab. 19, 667–681. 10.1016/j.cmet.2014.03.005 24703698 PMC4058888

[B455] Song Y. Keelan J. France J. T. (1996). Activin-A stimulates, while transforming growth factor beta 1 inhibits, chorionic gonadotrophin production and aromatase activity in cultured human placental trophoblasts. Placenta 17, 603–610. 10.1016/S0143-4004(96)80078-6 8916209

[B456] Sonier B. Lavigne C. Arseneault M. Ouellette R. Vaillancourt C. (2005). Expression of the 5-HT2A serotoninergic receptor in human placenta and choriocarcinoma cells: mitogenic implications of serotonin. Placenta 26, 484–490. 10.1016/j.placenta.2004.08.003 15950062

[B457] Sorenson R. L. Brelje T. C. (1997). Adaptation of islets of Langerhans to pregnancy: beta-cell growth, enhanced insulin secretion and the role of lactogenic hormones. Horm. Metab. Res. 29, 301–307. 10.1055/s-2007-979040 9230352

[B458] Sorenson R. L. Brelje T. C. Roth C. (1993). Effects of steroid and lactogenic hormones on islets of Langerhans: a new hypothesis for the role of pregnancy steroids in the adaptation of islets to pregnancy. Endocrinology 133, 2227–2234. 10.1210/endo.133.5.8404674 8404674

[B459] Sorenson R. L. Johnson M. G. Parsons J. A. Sheridan J. D. (1987). Decreased glucose stimulation threshold, enhanced insulin secretion, and increased beta cell coupling in islets of prolactin-treated rats. Pancreas 2, 283–288. 10.1097/00006676-198705000-00006 3306662

[B460] Spicer L. J. Aad P. Y. (2007). Insulin-like growth factor (IGF) 2 stimulates steroidogenesis and mitosis of bovine granulosa cells through the IGF1 receptor: role of follicle-stimulating hormone and IGF2 receptor. Biol. Reprod. 77, 18–27. 10.1095/biolreprod.106.058230 17360960

[B461] Steele G. L. Currie W. D. Yuen B. H. Jia X. C. Perlas E. Leung P. C. (1993). Acute stimulation of human chorionic gonadotropin secretion by recombinant human activin-A in first trimester human trophoblast. Endocrinology 133, 297–303. 10.1210/endo.133.1.8319577 8319577

[B462] Stelmanska E. Sucajtys-Szulc E. (2014). Enhanced food intake by progesterone-treated female rats is related to changes in neuropeptide genes expression in hypothalamus. Endokrynol. Pol. 65, 46–56. 10.5603/EP.2014.0007 24549602

[B463] Sternlicht M. D. (2006). Key stages in mammary gland development: the cues that regulate ductal branching morphogenesis. Breast Cancer Res. 8:201. 10.1186/bcr1368 16524451 PMC1413974

[B464] Stokkan K. A. Aarseth J. J. (2004). Melatonin reduces noradrenaline-induced vasoconstriction in the uterine artery of pregnant hooded seals (*Cystophora cristata*). Pflugers Arch. 447, 405–407. 10.1007/s00424-003-1198-5 14634822

[B465] Storment J. M. Meyer M. Osol G. (2000). Estrogen augments the vasodilatory effects of vascular endothelial growth factor in the uterine circulation of the rat. Am. J. Obstet. Gynecol. 183, 449–453. 10.1067/mob.2000.105910 10942485

[B466] Sun Y. Zupan B. Raaka B. M. Toth M. Gershengorn M. C. (2009). TRH-receptor-type-2-deficient mice are euthyroid and exhibit increased depression and reduced anxiety phenotypes. Neuropsychopharmacology 34, 1601–1608. 10.1038/npp.2008.217 19078951 PMC2669701

[B467] Takahashi K. Ohmichi M. Yoshida M. Hisamoto K. Mabuchi S. Arimoto-Ishida E.. (2003). Both estrogen and raloxifene cause G1 arrest of vascular smooth muscle cells. J. Endocrinol. 178, 319–329. 10.1677/joe.0.1780319 12904179

[B468] Takayanagi Y. Kasahara Y. Onaka T. Takahashi N. Kawada T. Nishimori K. (2008). Oxytocin receptor-deficient mice developed late-onset obesity. Neuroreport 19, 951–955. 10.1097/WNR.0b013e3283021ca9 18520999

[B469] Takayanagi Y. Yoshida M. Bielsky I. F. Ross H. E. Kawamata M. Onaka T.. (2005). Pervasive social deficits, but normal parturition, in oxytocin receptor-deficient mice. Proc. Natl. Acad. Sci. U.S.A. 102, 16096–16101. 10.1073/pnas.0505312102 16249339 PMC1276060

[B470] Takeda K. Toda K. Saibara T. Nakagawa M. Saika K. Onishi T.. (2003). Progressive development of insulin resistance phenotype in male mice with complete aromatase (CYP19) deficiency. J. Endocrinol. 176, 237–246. 10.1677/joe.0.1760237 12553872

[B471] Tamma R. Colaianni G. Zhu L. L. Dibenedetto A. Greco G. Montemurro G.. (2009). Oxytocin is an anabolic bone hormone. Proc. Natl. Acad. Sci. U.S.A. 106, 7149–7154. 10.1073/pnas.0901890106 19369205 PMC2678458

[B472] Tamura H. Takayama H. Nakamura Y. Reiter R. J. Sugino N. (2008). Fetal/placental regulation of maternal melatonin in rats. J. Pineal Res. 44, 335–340. 10.1111/j.1600-079X.2007.00537.x 18339129

[B473] Tessier C. Prigent-Tessier A. Bao L. Telleria C. M. Ferguson-Gottschall S. Gibori G. B.. (2003). Decidual activin: its role in the apoptotic process and its regulation by prolactin. Biol. Reprod. 68, 1687–1694. 10.1095/biolreprod.102.011684 12606360

[B474] Thomas A. L. Jack P. M. Manns J. G. Nathanielsz P. W. (1975). Effect of synthetic thyrotrophin releasing hormone on thyrotrophin and prolactin concentractions in the peripheral plasma of the pregnant ewe, lamb fetus and neonatal lamb. Biol. Neonate 26, 109–116. 10.1159/000240722 807265

[B475] Tiano J. P. Mauvais-Jarvis F. (2012). Importance of oestrogen receptors to preserve functional beta-cell mass in diabetes. Nat. Rev. Endocrinol. 8, 342–351. 10.1038/nrendo.2011.242 22330739

[B476] Tkachenko O. Shchekochikhin D. Schrier R. W. (2014). Hormones and hemodynamics in pregnancy. Int J Endocrinol Metab 12:e14098. 10.5812/ijem.14098 24803942 PMC4005978

[B477] Tomogane H. Mistry A. M. Voogt J. L. (1992). Late pregnancy and rat choriocarcinoma cells inhibit nocturnal prolactin surges and serotonin-induced prolactin release. Endocrinology 130, 23–28. 10.1210/endo.130.1.1727699 1727699

[B478] Toro A. R. Maymó J. L. Ibarbalz F. M. Pérez-Pérez A. Maskin B. Faletti A. G.. (2014). Leptin is an anti-apoptotic effector in placental cells involving p53 downregulation. PLoS ONE 9:e99187. 10.1371/journal.pone.0099187 24922063 PMC4055782

[B479] Trott J. F. Vonderhaar B. K. Hovey R. C. (2008). Historical perspectives of prolactin and growth hormone as mammogens, lactogens and galactagogues—agog for the future!. J. Mammary Gland Biol. Neoplasia 13, 3–11. 10.1007/s10911-008-9064-x 18204889

[B480] Ulrich U. Miller P. B. Eyre D. R. Chesnut C. H. III. Schlebusch H. Soules M. R. (2003). Bone remodeling and bone mineral density during pregnancy. Arch. Gynecol. Obstet. 268, 309–316. 10.1007/s00404-002-0410-8 14504876

[B481] Unemori E. N. Erikson M. E. Rocco S. E. Sutherland K. M. Parsell D. A. Mak J.. (1999). Relaxin stimulates expression of vascular endothelial growth factor in normal human endometrial cells *in vitro* and is associated with menometrorrhagia in women. Hum. Reprod. 14, 800–806. 10.1093/humrep/14.3.800 10221717

[B482] Urbanek M. O. Nawrocka A. U. Krzyzosiak W. J. (2015). Small RNA Detection by in Situ Hybridization Methods. Int. J. Mol. Sci. 16, 13259–13286. 10.3390/ijms160613259 26068454 PMC4490494

[B483] Vaccarello M. A. Diamond F. B. Jr. Guevara-Aguirre J. Rosenbloom A. L. Fielder P. J. Gargosky S.. (1993). Hormonal and metabolic effects and pharmacokinetics of recombinant insulin-like growth factor-I in growth hormone receptor deficiency/Laron syndrome. J. Clin. Endocrinol. Metab. 77, 273–280. 7686916 10.1210/jcem.77.1.7686916

[B484] Vale W. Blackwell R. Grant G. Guillemin R. (1973). TRF and thyroid hormones on prolactin secretion by rat anterior pituitary cells *in vitro*. Endocrinology 93, 26–33. 10.1210/endo-93-1-26 4351315

[B485] Valsamakis G. Kumar S. Creatsas G. Mastorakos G. (2010). The effects of adipose tissue and adipocytokines in human pregnancy. Ann. N. Y. Acad. Sci. 1205, 76–81. 10.1111/j.1749-6632.2010.05667.x 20840256

[B486] Van Bodegraven A. A. Böhmer C. J. Manoliu R. A. Paalman E. Van Der Klis A. H. Roex A. J.. (1998). Gallbladder contents and fasting gallbladder volumes during and after pregnancy. Scand. J. Gastroenterol. 33, 993–997. 10.1080/003655298750027047 9759958

[B487] Vanhouten J. N. Dann P. Stewart A. F. Watson C. J. Pollak M. Karaplis A. C.. (2003). Mammary-specific deletion of parathyroid hormone-related protein preserves bone mass during lactation. J. Clin. Invest. 112, 1429–1436. 10.1172/JCI200319504 14597768 PMC228471

[B488] Van Leengoed E. Kerker E. Swanson H. H. (1987). Inhibition of post-partum maternal behaviour in the rat by injecting an oxytocin antagonist into the cerebral ventricles. J. Endocrinol. 112, 275–282. 10.1677/joe.0.1120275 3819639

[B489] Vannuccini S. Bocchi C. Severi F. M. Challis J. R. Petraglia F. (2016). Endocrinology of human parturition. Ann. Endocrinol. (Paris). 77, 105–113. 10.1016/j.ando.2016.04.025 27155774

[B490] Vasavada R. C. Cavaliere C. D'ercole A. J. Dann P. Burtis W. J. Madlener A. L.. (1996). Overexpression of parathyroid hormone-related protein in the pancreatic islets of transgenic mice causes islet hyperplasia, hyperinsulinemia, and hypoglycemia. J. Biol. Chem. 271, 1200–1208. 10.1074/jbc.271.2.1200 8557651

[B491] Vasavada R. C. Garcia-Ocaña A. Zawalich W. S. Sorenson R. L. Dann P. Syed M.. (2000). Targeted expression of placental lactogen in the beta cells of transgenic mice results in beta cell proliferation, islet mass augmentation, and hypoglycemia. J. Biol. Chem. 275, 15399–15406. 10.1074/jbc.275.20.15399 10809775

[B492] Veenstra Van Nieuwenhoven A. L. Bouman A. Moes H. Heineman M. J. De Leij L. F. Santema J.. (2002). Cytokine production in natural killer cells and lymphocytes in pregnant women compared with women in the follicular phase of the ovarian cycle. Fertil. Steril. 77, 1032–1037. 10.1016/S0015-0282(02)02976-X 12009363

[B493] Villar J. Cogswell M. Kestler E. Castillo P. Menendez R. Repke J. T. (1992). Effect of fat and fat-free mass deposition during pregnancy on birth weight. Am. J. Obstet. Gynecol. 167, 1344–1352. 10.1016/S0002-9378(11)91714-1 1442988

[B494] Vodstrcil L. A. Tare M. Novak J. Dragomir N. Ramirez R. J. Wlodek M. E.. (2012). Relaxin mediates uterine artery compliance during pregnancy and increases uterine blood flow. FASEB J. 26, 4035–4044. 10.1096/fj.12-210567 22744867 PMC3448779

[B495] Voltolini C. Petraglia F. (2014). Neuroendocrinology of pregnancy and parturition. Handb. Clin. Neurol. 124, 17–36. 10.1016/B978-0-444-59602-4.00002-2 25248577

[B496] Wagner K. U. Young W. S. III. Liu X. Ginns E. I. Li M. Furth P. A.. (1997). Oxytocin and milk removal are required for post-partum mammary-gland development. Genes Funct. 1, 233–244. 10.1046/j.1365-4624.1997.00024.x 9678900

[B497] Wallace J. M. Robinson J. J. Wigzell S. Aitken R. P. (1988). Effect of melatonin on the peripheral concentrations of LH and progesterone after oestrus, and on conception rate in ewes. J. Endocrinol. 119, 523–530. 10.1677/joe.0.1190523 3221157

[B498] Wang J. W. Jiang Y. N. Huang C. Y. Huang P. Y. Huang M. C. Cheng W. T.. (2006). Proliferin enhances microvilli formation and cell growth of neuroblastoma cells. Neurosci. Res. 56, 80–90. 10.1016/j.neures.2006.05.011 16876275

[B499] Wang S. J. Liu W. J. Wang L. K. Pang X. S. Yang L. G. (2017). The role of Melatonin receptor MTNR1A in the action of Melatonin on bovine granulosa cells. Mol. Reprod. Dev. 84, 1140–1154. 10.1002/mrd.22877 28805353

[B500] Weaver S. R. Prichard A. P. Endres E. L. Newhouse S. A. Peters T. L. Crump P. M.. (2016). Elevation of circulating serotonin improves calcium dynamics in the peripartum dairy cow. J. Endocrinol. 230, 105–123. 10.1530/JOE-16-0038 27390301

[B501] Weaver S. R. Prichard A. S. Maerz N. L. Prichard A. P. Endres E. L. Hernández-Castellano L. E.. (2017). Elevating serotonin pre-partum alters the Holstein dairy cow hepatic adaptation to lactation. PLoS ONE 12:e0184939. 10.1371/journal.pone.0184939 28922379 PMC5602632

[B502] Weil Z. M. Hotchkiss A. K. Gatien M. L. Pieke-Dahl S. Nelson R. J. (2006). Melatonin receptor (MT1) knockout mice display depression-like behaviors and deficits in sensorimotor gating. Brain Res. Bull. 68, 425–429. 10.1016/j.brainresbull.2005.09.016 16459197

[B503] Weinberger S. E. Weiss S. T. Cohen W. R. Weiss J. W. Johnson T. S. (1980). Pregnancy and the lung. Am. Rev. Respir. Dis. 121, 559–581. 10.1164/arrd.1980.121.3.559 6998334

[B504] Weiner C. P. Lizasoain I. Baylis S. A. Knowles R. G. Charles I. G. Moncada S. (1994). Induction of calcium-dependent nitric oxide synthases by sex hormones. Proc. Natl. Acad. Sci. USA. 91, 5212–5216. 10.1073/pnas.91.11.5212 7515189 PMC43962

[B505] Weinhaus A. J. Stout L. E. Sorenson R. L. (1996). Glucokinase, hexokinase, glucose transporter 2, and glucose metabolism in islets during pregnancy and prolactin-treated islets *in vitro*: mechanisms for long term up-regulation of islets. Endocrinology 137, 1640–1649. 10.1210/endo.137.5.8612496 8612496

[B506] Weir E. C. Philbrick W. M. Amling M. Neff L. A. Baron R. Broadus A. E. (1996). Targeted overexpression of parathyroid hormone-related peptide in chondrocytes causes chondrodysplasia and delayed endochondral bone formation. Proc. Natl. Acad. Sci. U.S.A. 93, 10240–10245. 10.1073/pnas.93.19.10240 8816783 PMC38368

[B507] Weisinger R. S. Burns P. Eddie L. W. Wintour E. M. (1993). Relaxin alters the plasma osmolality-arginine vasopressin relationship in the rat. J. Endocrinol. 137, 505–510. 10.1677/joe.0.1370505 8371080

[B508] Wennbo H. Kindblom J. Isaksson O. G. Törnell J. (1997). Transgenic mice overexpressing the prolactin gene develop dramatic enlargement of the prostate gland. Endocrinology 138, 4410–4415. 10.1210/endo.138.10.5461 9322957

[B509] Whitehead C. L. Walker S. P. Ye L. Mendis S. Kaitu'u-Lino T. J. Lappas M.. (2013). Placental specific mRNA in the maternal circulation are globally dysregulated in pregnancies complicated by fetal growth restriction. J. Clin. Endocrinol. Metab. 98, E429–436. 10.1210/jc.2012-2468 23337725

[B510] White V. González E. Capobianco E. Pustovrh C. Martínez N. Higa R.. (2006). Leptin modulates nitric oxide production and lipid metabolism in human placenta. Reprod. Fertil. Dev. 18, 425–432. 10.1071/RD05105 16737635

[B511] Wiemers D. O. Shao L.-J. Ain R. Dai G. Soares M. J. (2003). The mouse prolactin gene family locus. Endocrinology 144, 313–325. 10.1210/en.2002-220724 12488360

[B512] Williams E. D. Leaver D. D. Danks J. A. Moseley J. M. Martin T. J. (1994). Effect of parathyroid hormone-related protein (PTHrP) on the contractility of the myometrium and localization of PTHrP in the uterus of pregnant rats. J. Reprod. Fertil. 102, 209–214. 10.1530/jrf.0.1020209 7799315

[B513] Williams E. D. Major B. J. Martin T. J. Moseley J. M. Leaver D. D. (1998). Effect of antagonism of the parathyroid hormone (PTH)/PTH-related protein receptor on decidualization in rat uterus. J. Reprod. Fertil. 112, 59–67. 10.1530/jrf.0.1120059 9538330

[B514] Wilson T. Liggins G. C. Whittaker D. J. (1988). Oxytocin stimulates the release of arachidonic acid and prostaglandin F2 alpha from human decidual cells. Prostaglandins 35, 771–780. 10.1016/0090-6980(88)90149-9 2840690

[B515] Winter E. M. Appelman-Dijkstra N. M. (2017). Parathyroid hormone-related protein-induced hypercalcemia of pregnancy successfully reversed by a dopamine agonist. J. Clin. Endocrinol. Metab. 102, 4417–4420. 10.1210/jc.2017-01617 29053801

[B516] Wu H. H. Choi S. Levitt P. (2016). Differential patterning of genes involved in serotonin metabolism and transport in extra-embryonic tissues of the mouse. Placenta 42, 74–83. 10.1016/j.placenta.2016.03.013 27238716 PMC4886340

[B517] Wysolmerski J. J. Mccaughern-Carucci J. F. Daifotis A. G. Broadus A. E. Philbrick W. M. (1995). Overexpression of parathyroid hormone-related protein or parathyroid hormone in transgenic mice impairs branching morphogenesis during mammary gland development. Development 121, 3539–3547. 8582268 10.1242/dev.121.11.3539

[B518] Xiang S. Mao L. Yuan L. Duplessis T. Jones F. Hoyle G. W.. (2012). Impaired mouse mammary gland growth and development is mediated by melatonin and its MT1G protein-coupled receptor via repression of ERalpha, Akt1, and Stat5. J. Pineal Res. 53, 307–318. 10.1111/j.1600-079X.2012.01000.x 22582905 PMC3422609

[B519] Yamada M. Saga Y. Shibusawa N. Hirato J. Murakami M. Iwasaki T.. (1997). Tertiary hypothyroidism and hyperglycemia in mice with targeted disruption of the thyrotropin-releasing hormone gene. Proc. Natl. Acad. Sci. U.S.A. 94, 10862–10867. 10.1073/pnas.94.20.10862 9380725 PMC23510

[B520] Yamada M. Shibusawa N. Ishii S. Horiguchi K. Umezawa R. Hashimoto K.. (2006). Prolactin secretion in mice with thyrotropin-releasing hormone deficiency. Endocrinology 147, 2591–2596. 10.1210/en.2005-1326 16484326

[B521] Yamaguchi M. Endo H. Tasaka K. Miyake A. (1995). Mouse growth hormone-releasing factor secretion is activated by inhibin and inhibited by activin in placenta. Biol. Reprod. 53, 368–372. 10.1095/biolreprod53.2.368 7492689

[B522] Yamashita H. Shao J. Ishizuka T. Klepcyk P. J. Muhlenkamp P. Qiao L.. (2001). Leptin administration prevents spontaneous gestational diabetes in heterozygous Lepr(db/+) mice: effects on placental leptin and fetal growth. Endocrinology 142, 2888–2897. 10.1210/endo.142.7.8227 11416008

[B523] Yao L. Agoulnik A. I. Cooke P. S. Meling D. D. Sherwood O. D. (2008). Relaxin acts on stromal cells to promote epithelial and stromal proliferation and inhibit apoptosis in the mouse cervix and vagina. Endocrinology 149, 2072–2079. 10.1210/en.2007-1176 18218691 PMC2329284

[B524] Yeh S. Tsai M. Y. Xu Q. Mu X. M. Lardy H. Huang K. E.. (2002). Generation and characterization of androgen receptor knockout (ARKO) mice: an *in vivo* model for the study of androgen functions in selective tissues. Proc. Natl. Acad. Sci. U.S.A. 99, 13498–13503. 10.1073/pnas.212474399 12370412 PMC129702

[B525] Yellon S. M. Longo L. D. (1988). Effect of maternal pinealectomy and reverse photoperiod on the circadian melatonin rhythm in the sheep and fetus during the last trimester of pregnancy. Biol. Reprod. 39, 1093–1099. 10.1095/biolreprod39.5.1093 3219382

[B526] Yogosawa S. Mizutani S. Ogawa Y. Izumi T. (2013). Activin receptor-like kinase 7 suppresses lipolysis to accumulate fat in obesity through downregulation of peroxisome proliferator-activated receptor gamma and C/EBPalpha. Diabetes 62, 115–123. 10.2337/db12-0295 22933117 PMC3526038

[B527] Yong H. E. J. Murthi P. Kalionis B. Keogh R. J. Brennecke S. P. (2017). Decidual ACVR2A regulates extravillous trophoblast functions of adhesion, proliferation, migration and invasion i*n vitro*. Pregnancy Hypertens. 12, 189–193. 10.1016/j.preghy.2017.11.002 29203340

[B528] Yong H. E. Murthi P. Wong M. H. Kalionis B. Cartwright J. E. Brennecke S. P.. (2015). Effects of normal and high circulating concentrations of activin A on vascular endothelial cell functions and vasoactive factor production. Pregnancy Hypertens. 5, 346–353. 10.1016/j.preghy.2015.09.006 26597752

[B529] Young W. S. III. Shepard E. Amico J. Hennighausen L. Wagner K. U. Lamarca M. E. (1996). Deficiency in mouse oxytocin prevents milk ejection, but not fertility or parturition. J. Neuroendocrinol. 8, 847–853. 10.1046/j.1365-2826.1996.05266.x 8933362

[B530] Youssef R. E. Ledingham M. A. Bollapragada S. S. O'gorman N. Jordan F. Young A.. (2009). The role of toll-like receptors (TLR-2 and−4) and triggering receptor expressed on myeloid cells 1 (TREM-1) in human term and preterm labor. Reprod. Sci. 16, 843–856. 10.1177/1933719109336621 19564644

[B531] Yu L. Li D. Liao Q. P. Yang H. X. Cao B. Fu G.. (2012). High levels of activin A detected in preeclamptic placenta induce trophoblast cell apoptosis by promoting nodal signaling. J. Clin. Endocrinol. Metab. 97, E1370–E1379. 10.1210/jc.2011-2729 22685232

[B532] Yura S. Ogawa Y. Sagawa N. Masuzaki H. Itoh H. Ebihara K.. (2000). Accelerated puberty and late-onset hypothalamic hypogonadism in female transgenic skinny mice overexpressing leptin. J. Clin. Invest. 105, 749–755. 10.1172/JCI8353 10727443 PMC377463

[B533] Zeng H. Schimpf B. A. Rohde A. D. Pavlova M. N. Gragerov A. Bergmann J. E. (2007). Thyrotropin-releasing hormone receptor 1-deficient mice display increased depression and anxiety-like behavior. Mol. Endocrinol. 21, 2795–2804. 10.1210/me.2007-0048 17666589

[B534] Zhang L. Fishman M. C. Huang P. L. (1999). Estrogen mediates the protective effects of pregnancy and chorionic gonadotropin in a mouse model of vascular injury. Arterioscler. Thromb. Vasc. Biol. 19, 2059–2065. 10.1161/01.ATV.19.9.2059 10479646

[B535] Zhang Y. Hou Y. Wang X. Ping J. Ma Z. Suo C.. (2017). The effects of kisspeptin-10 on serum metabolism and myocardium in rats. PLoS ONE 12:e0179164. 10.1371/journal.pone.0179164 28692647 PMC5503227

[B536] Zhao L. Roche P. J. Gunnersen J. M. Hammond V. E. Tregear G. W. Wintour E. M.. (1999). Mice without a functional relaxin gene are unable to deliver milk to their pups. Endocrinology 140, 445–453. 10.1210/endo.140.1.6404 9886856

[B537] Zhao L. Samuel C. S. Tregear G. W. Beck F. Wintour E. M. (2000). Collagen studies in late pregnant relaxin null mice. Biol. Reprod. 63, 697–703. 10.1095/biolreprod63.3.697 10952910

[B538] Zha W. Ho H. T. B. Hu T. Hebert M. F. Wang J. (2017). Serotonin transporter deficiency drives estrogen-dependent obesity and glucose intolerance. Sci. Rep. 7:1137. 10.1038/s41598-017-01291-5 28442777 PMC5430688

[B539] Zhou B. Kong X. Linzer D. I. (2005). Enhanced recovery from thrombocytopenia and neutropenia in mice constitutively expressing a placental hematopoietic cytokine. Endocrinology 146, 64–70. 10.1210/en.2004-1011 15375031

[B540] Zhou B. Lum H. E. Lin J. Linzer D. I. (2002). Two placental hormones are agonists in stimulating megakaryocyte growth and differentiation. Endocrinology 143, 4281–4286. 10.1210/en.2002-220447 12399423

[B541] Zhou Y. Xu B. C. Maheshwari H. G. He L. Reed M. Lozykowski M.. (1997). A mammalian model for Laron syndrome produced by targeted disruption of the mouse growth hormone receptor/binding protein gene (the Laron mouse). Proc. Natl. Acad. Sci. U.S.A. 94, 13215–13220. 10.1073/pnas.94.24.13215 9371826 PMC24289

[B542] Zhu Y. Bian Z. Lu P. Karas R. H. Bao L. Cox D.. (2002). Abnormal vascular function and hypertension in mice deficient in estrogen receptor beta. Science 295, 505–508. 10.1126/science.1065250 11799247

[B543] Ziegler B. Lucke S. Besch W. Hahn H. J. (1985). Pregnancy-associated changes in the endocrine pancreas of normoglycaemic streptozotocin-treated Wistar rats. Diabetologia 28, 172–175. 3888760 10.1007/BF00273867

[B544] Zöllner J. Howe L. G. Edey L. F. O'dea K. P. Takata M. Gordon F.. (2017). The response of the innate immune and cardiovascular systems to LPS in pregnant and nonpregnant mice. Biol. Reprod. 97, 258–272. 10.1093/biolre/iox076 29044422

[B545] Zygmunt M. Herr F. Keller-Schoenwetter S. Kunzi-Rapp K. Münstedt K. Rao C. V.. (2002). Characterization of human chorionic gonadotropin as a novel angiogenic factor. J. Clin. Endocrinol. Metab. 87, 5290–5296. 10.1210/jc.2002-020642 12414904

